# Application Value of Radiomics-Based Machine Learning for Preoperative Risk Stratification of Bladder Cancer: Systematic Review and Meta-Analysis

**DOI:** 10.2196/81084

**Published:** 2026-06-12

**Authors:** Zirong He, Yinghua Liu, Qin Jiang, Fang Hou, Bing Xu

**Affiliations:** 1 Department of Pediatric Surgery, Sichuan Provincial People's Hospital, School of Medicine, University of Electronic Science and Technology of China, No. 32 West Second Section, First Ring Road, Qingyang District, Chengdu, Sichuan, 610072, China, 86 18981838032; 2 Sichuan Provincial People's Hospital East Sichuan Hospital & Dazhou First People's Hospital, Dazhou, China

**Keywords:** radiomics, bladder cancer, muscle invasion, pathologic grading, risk classification

## Abstract

**Background:**

Some researchers have explored the application of radiomics-based machine learning to detect preoperative muscle invasion, high-grade tumors, human epidermal growth factor receptor 2 expression, and other risk factors for bladder cancer. However, systematic evidence proving its effectiveness remains lacking.

**Objective:**

This study aimed to evaluate the performance of radiomics-based machine learning in preoperative risk stratification for patients with bladder cancer. These findings could contribute to advancing the development or updating of intelligent risk assessment tools for bladder cancer.

**Methods:**

The Embase, Cochrane Library, PubMed, and Web of Science databases were systematically retrieved for publicly available studies on the effectiveness of radiomics-based machine learning (ML) in the preoperative risk stratification of bladder cancer up to October 17, 2025. The risk of bias in the included studies was evaluated using the Prediction Model Risk of Bias Assessment Tool for Artificial Intelligence. The overall quality of the studies was quantified using the Radiomics Quality Scoring tool. The certainty of the evidence was graded using the Grading of Recommendations, Assessment, Development, and Evaluation (GRADE) framework. Subgroup analyses were conducted according to the type of imaging source and modeling method.

**Results:**

This meta-analysis ultimately incorporated 57 studies with a total of 11,933 participants. These studies primarily used radiomics-based ML to identify muscle invasion (n=34) and high-grade tumors (n=16). Additionally, the methodology was used to evaluate human epidermal growth factor receptor 2 positive expression (n=3), Ki-67 expression (n=2), and lymph node staging (n=2) preoperatively in bladder cancer. In the validation sets, the pooled area under the receiver operating characteristic curve (AUROC) for identifying muscle invasion was 0.893 (95% CI 0.840-0.948), 0.916 (95% CI 0.891-0.942), and 0.840 (95% CI 0.737-0.958) for computed tomography (CT)–, magnetic resonance imaging (MRI)–, and ultrasound-based radiomics, respectively. The AUROC was 0.874 (95% CI 0.852-0.896) and 0.921 (95% CI 0.867-0.979) for models integrating clinical features with CT- or MRI-based radiomics, respectively. The pooled AUROC for diagnosing high-grade tumors was 0.874 (95% CI 0.775-0.985), 0.846 (95% CI 0.663-1.000), and 0.750 (95% CI 0.636-0.884) for CT-, MRI-, and ultrasound-based radiomics, respectively. Furthermore, the AUROC was 0.919 (95% CI 0.774-1.000) for MRI-based radiomics combined with clinical features.

**Conclusions:**

This is the first systematic review to comprehensively evaluate the role of radiomics in preoperative risk stratification for bladder cancer. It provides evidence to inform the development and refinement of future ML-based tools for image analysis in this setting. However, this evidence faces significant challenges, including methodological shortcomings and a high risk of bias and low GRADE level, which preclude its readiness for clinical translation. Future studies should standardize the methodological workflows in radiomics, conduct multicenter research, and thoroughly evaluate and discuss the validity of external validation.

## Introduction

Bladder cancer is the tenth most common cancer globally. It ranks fourth in new cancer cases in men and is the eighth leading cause of cancer death [1,2]. In the United States, an estimated 83,190 new cases of bladder cancer occurred in 2024 (63,070 in men and 20,120 in women), resulting in 16,840 deaths (12,290 in men and 4550 in women) [2]. Thus, bladder cancer has become a serious social burden.

Transurethral resection of bladder tumors (TURBT), followed by pathological analysis, provides the basis for diagnosing, staging, and treating bladder cancer [3]. Nonetheless, this procedure is imprecise in assessing muscle invasion. The absence of detrusor muscle in the specimen is linked to a significantly elevated risk of residual lesions, early recurrence, and understaging of tumor [3,4]. TURBT also carries a few operational risks. A recent study of the learning curve for TURBT indicates that urologists should perform at least 100 TURBT procedures before achieving acceptable oncological outcomes and meeting the minimum requirements for surgical success. Furthermore, the study found that the first 45 TURBT procedures resulted in the worst outcomes [5]. Repeat transurethral resection provides a second chance in such cases. While repeat transurethral resection holds potential for improving tumor prognosis, evidence demonstrating its capacity to confer a survival benefit remains inconclusive [6,7]. Like TURBT, cystoscopy is an invasive procedure with risks of insufficient sampling and understaging. This creates a need for noninvasive, accurate diagnostic alternatives [8,9].

Magnetic resonance imaging (MRI) and computed tomography (CT) have been used to provide additional staging information. Nevertheless, neither technique can accurately evaluate microscopic infiltrates. Both of them aim to confirm or exclude locally advanced disease (stage ≥T3b) [10]. Therefore, exploring new preoperative techniques that can effectively identify the risk of muscle invasion and pathological grading of bladder cancer is clinically important.

In recent years, radiomics has received significant attention from researchers. Like therapies inspired by molecular biology, radiomics shows great promise in advancing precision medicine. This technique can predict outcomes individually or in combination with genomic, comorbidity, clinical, or demographic data. The process involves acquiring images, identifying volumes of interest (ie, those with possible prognostic value), segmenting the volume (ie, depicting the boundaries of the volume with computer-assisted contouring), and extracting and qualifying descriptive features from the volume. These features are then used to populate searchable databases, which are subsequently mined to develop classifier models. Radiomics shows promise as a quantitative imaging biomarker for both characterizing bladder cancer and predicting its prognosis [11-14]. At the time of this writing, high-throughput computing enables the rapid extraction of numerous quantitative features from tomographic images (eg, CT, magnetic resonance, or positron emission tomography). Radiomics transforms medical images into quantifiable data for analysis. The motivation stems from the wealth of pathophysiological information embedded in medical images, which quantitative analysis can unlock to enhance decision-making [11,13,15]. This algorithmic assistance may provide more accurate histopathological diagnoses, save time, and increase clinician confidence, contributing to improved prognosis [12,16-18]. Recently, as radiomics has developed in oncological diagnosis and treatment, researchers have examined the application of radiomics-based machine learning (ML) in identifying muscle invasion [18-21] and high-grade tumors [15], as well as in determining human epidermal growth factor receptor 2 (HER2) and Ki-67 expression and lymph node (LN) staging for bladder cancer. Compared with genomic biomarkers, artificial intelligence (AI) systems leveraging digitized images offer a more cost-effective and readily scalable solution. These tools can continuously learn from new data, thereby enhancing their predictive performance and, consequently, their value to health care over time [22]. However, comprehensive and systematic evidence of its effectiveness remains lacking.

To address this gap, we conducted a meta-analysis aimed at systematically evaluating the application of radiomics-based ML to preoperative risk stratification in bladder cancer and quantitatively summarizing its diagnostic accuracy. The findings may establish an evidence base for developing and refining future image-based, AI-assisted tools of risk stratification.

## Methods

### Study Registration

This study followed the PRISMA (Preferred Reporting Items for Systematic Reviews and Meta-Analyses) 2020 guidelines (Checklist 1). The review was prospectively registered with PROSPERO (International Prospective Register of Systematic Reviews) under the title “Assessment of Preoperative Risk Stratification for Bladder Cancer Using Machine Learning Based on Radiomics: A Systematic Review and Meta-Analysis” (CRD42024561649).

### Eligibility Criteria

#### Inclusion Criteria

The inclusion criteria were as follows:

1. Patients with bladder cancer diagnosed by biopsy;
2. A complete ML model covering radiomics features was constructed to identify muscle invasion, high-grade tumors, HER2, LN staging, and Ki-67 expression;
3. Studies reported in English.

#### Exclusion Criteria

The exclusion criteria were as follows:

1. Unpublished conference abstracts;
2. Studies with image segmentation only and without complete ML models for identifying muscle invasion, high-grade tumors, HER2, LN staging, and Ki-67 expression;
3. Studies that only performed texture analysis, with no ML classifiers constructed based on extracted textures;
4. Lack of metrics (eg, sensitivity or recall, area under the receiver operating characteristic curve [AUROC], specificity, calibration curves, accuracy, confusion matrix, precision, and *F*_1_-score) for predicting the accuracy of ML models.

### Search Strategy and Data Source

The Cochrane Library (CENTRAL), Embase, PubMed, and Web of Science were retrieved up to June 17, 2024. This investigation did not perform simultaneous searches across multiple databases on a single platform. Searches were not extended to dedicated conference abstract databases or web sources beyond the primary databases. The authors of unpublished conference abstracts were not contacted to obtain full study details. To mitigate the risk of omitting relevant studies, the reference lists of eligible articles and pertinent review papers were manually scrutinized. No search filters were applied. The search strategy integrated both controlled vocabulary, such as Medical Subject Headings and Emtree terms, and free-text keywords to optimize sensitivity. Subject headings included “Urinary Bladder Neoplasms” and “machine learning.” Search strategies were tailored to the specific syntax of each database and combined using Boolean operators. No restrictions were applied concerning publication date or geographical location. Before the final data analysis, an updated search was performed in all specified databases on October 17, 2025 (Multimedia Appendix 1).

### Selection of Studies and Data Extraction

EndNote was used to import the retrieved articles. After eliminating duplicate records, the remaining articles were reviewed by titles and abstracts. Exclusions were categorized as follows: meta-analyses or reviews, replies or letters, case reports, animal experiments, registry or clinical trial protocols, non-English articles, and preprints. Initially relevant articles were screened by full text to determine eligible studies.

Before data extraction, a spreadsheet was created. The collected information encompassed DOI, title, publication year, first author, country, study type, purpose of the task, patient source, image source, recording of a complete image acquisition protocol, number of researchers involved, whether preliminary experiments tested different imaging parameters, radiomic region of interest (ROI) segmentation software, total number of outcome events, whether test-retest experiments were performed, total number of cases, training set size, number of outcome events in the training set, validation set generation method, validation set size, number of outcome events in the validation set, variable selection method, model types used, overfitting assessment method, public availability of data and code, mean age, sex, specimen source, AUROC (95% CI), number of true negatives, number of true positives, specificity, sensitivity, precision, accuracy, and *F*_1_-score.

Results from each dataset within a study were included only once. When multiple studies published by the same author over different years were suspected of having overlapping datasets, only the study with the larger sample size was included. When multiple models were present, the model demonstrating optimal performance in the validation set was selected for inclusion. If a single dataset contained multiple validation sets, all were incorporated into the analysis.

Two researchers (ZH and YL) screened the articles and extracted the data separately. Their results were cross-checked. Any discrepancies were addressed with the help of a third researcher (BX).

### Assessment of Risk of Bias and Quality

The Prediction Model Risk of Bias Assessment Tool for Artificial Intelligence (PROBAST-AI) provides a framework to critically appraise the risk of bias (ROB) and applicability of ML-based multivariable prediction models [23,24]. The assessment for ROB comprises four domains: participants and data sources, predictors, outcome, and analysis (1=strongly disagree, 2=somewhat disagree, 3=I don’t know, 4=somewhat agree, 5=strongly agree). A domain was judged as high risk if any signaling question was rated “no/probably no,” and as low risk only if all questions were answered “yes/probably yes.” An “I don’t know” response yielded an unclear risk designation.

Furthermore, the quality of the eligible investigations was appraised using the Radiomics Quality Score (RQS), which ranges from –8 to 36 [25]. This RQS primarily considers image protocol quality, multiple image segmentation methods, study of modalities, image acquisition time, feature reduction, and model construction using radiomic and nonradiomic features (prognosis and molecular subtyping). It also considers the detection and discussion of radiomic and biological correlations, cutoff value analysis, calibration statistics, discrimination statistics, validation, prospective studies registered in trial databases, comparison with the “gold standard,” cost-effectiveness analysis, potential clinical utility, and open science and data.

Two investigators independently evaluated the ROB and methodological quality and then performed a cross-check of their assessments. Any discrepancies were adjudicated with the help of a third researcher (BX).

### Synthesis Methods

A meta-analysis was performed on AUROC, a metric for the accuracy of ML models. The analysis required the SE or 95% CI of the AUROC. However, some of the included studies lacked a 95% CI and SE. In this scenario, SE was estimated by referring to the study by Debray et al [26]. A random-effects model was used for the meta-analysis to account for potential heterogeneity among the primary studies due to variations in model parameter tuning and predictor selection. Furthermore, the Hartung-Knapp-Sidik-Jonkman method was applied [27].

Furthermore, a meta-analysis was performed using a bivariate mixed effects model to pool sensitivity and specificity. This analysis was based on diagnostic contingency tables. Nevertheless, these tables were not directly provided in some studies. Then, the required values were calculated via reported sensitivity, specificity, precision, and sample sizes. Subsequently, subgroup analyses were conducted based on the dataset (training and validation sets), image source, and model type. A meta-analysis of AUROC values was conducted using packages “*meta”* and “*metafor”* in R (v4.5.2; R Foundation for Statistical Computing). A bivariate mixed effects model was implemented using the “*midas”* package in Stata (v15.0; StataCorp LLC).


$$SE(c)\approx \sqrt{\frac{c(1-c)\left[1+\frac{n*(1-c)}{2-c}+\frac{m*c}{1+c}\right]}{mn}}$$

Parameter definitions: c denotes the c-statistic; n represents the number of observed events; and m corresponds to the total sample size $n^{*}=m^{*}=\frac{m+n}{2}-1$.

Finally, subgroup analyses were conducted within each task category based on case number, ML type (logistic regression [LR] vs other ML), and modeling variables (radiomics features alone vs radiomics features+clinical characteristics).

### Certainty of Evidence

The certainty of evidence for the AUROC estimates derived from various subgroup analyses was evaluated using the Grading of Recommendations, Assessment, Development, and Evaluation (GRADE) framework. Two investigators independently performed the evidence grading and a cross-verification of their assessments. Any discrepancies were adjudicated by a third investigator (BX).

## Results

### Study Selection

**Figure 1.**
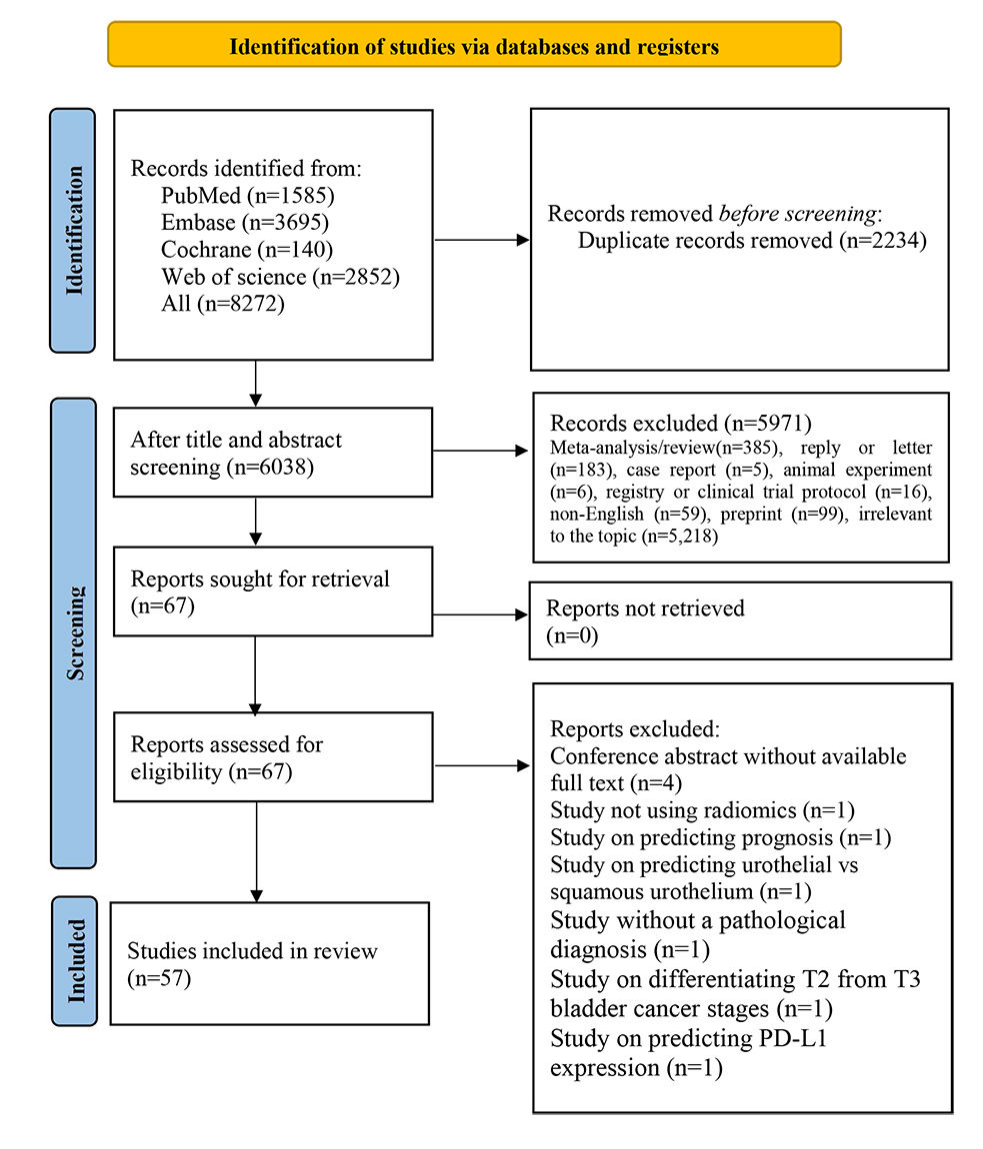
PRISMA (Preferred Reporting Items for Systematic Reviews and Meta-Analyses) of the literature selection process.

The database search yielded 8272 articles. After removing 2234 duplicates, 6038 articles remained and were reviewed based on their titles and abstracts. This process resulted in the exclusion of 5971 ineligible records. Overall, 67 articles remained and were reviewed by full texts, leading to the removal of 10 for irrelevance, including 4 non–peer-reviewed conference papers, one study lacking radiomics analysis, one focused on differentiating pure urothelial carcinoma from urothelial carcinoma with squamous differentiation, one without radiomics, one enrolling participants without a pathological diagnosis, one differentiating T2 from T3 bladder cancer stages, and one predicting programmed death-ligand 1 expression. Ultimately, the meta-analysis incorporated 57 articles [28-84] (Figure 1).

### Characteristics of Studies

Fifty-seven [28-84] eligible studies, published between 2017 and 2025, encompassed data on 11,933 individuals with bladder cancer. Most were case-control studies, including 40 [28,33-36,38-41,43-46,48,49,51,53-57,61-76,79,82,84] single-center and 17 [29-32,37,42,47,50,52,58-60,77,78,80,81,83] multicenter studies. Thirty-four studies primarily investigated muscle invasion; 20 [30,32,33,41-43,46,51,52,54,57-60,63,64,69,71,78,83], 13[28,29,31,34,45,48,50,62,75,79-82], and one [65] used MRI-, CT-, and ultrasound-based radiomics, respectively. Sixteen studies [36,38-40,44,47,49,53,56,61,66,67,70,72,73,84] focused on discriminating high-grade tumors. Three studies [55,74,77] examined HER2-positive expression, 2 [35,76] examined Ki-67 expression, and 2 [37,68] examined LN staging in bladder cancer. ITK-SNAP (University of Pennsylvania) was the most frequently used software for ROI segmentation. Other software platforms included MATLAB (R2012b; Matrix Laboratory; MathWorks), the open-source Medical Imaging Interaction Toolkit, and a computer-assisted visualization and analysis software system. Fifty-three studies explicitly described the generation of a validation; among these, 37 [28,34-37,39-41,43-46,48,49,51,53-57,61-66,68-72,74-76,79,82,84] used internal validation techniques such as random sampling or cross-validation and 16 [29-32,42,47,50,52,58-60,77,78,80,81,83] studies used external validation. A total of 10 distinct model types were evaluated (Table 1).

**Table 1.** Basic information.

Studies	Country	Study type	Source of the patient	Age (years)	Gender (n)	Task	Radiomics source	Specimens source	Number of image researcher	ROI^a^ segmentation software	The total number of cases (N)	Number of cases in the training set (n)	Generation method of validation set	Number of cases in validation set	Model type
Zhang et al (2024) [28]	China	Case-control	Single center	Total, mean (SD): 67.821 (10.468) Training set, mean (SD): 68.066 (11.184) Validation set, mean (SD): 67.254 (8.64)	Total: F^b^: 36; M^c^: 160; Training set: F: 20; M: 117; Validation set: F: 16; M: 43	Muscle invasion status	CT^d^	Pathology (TURBT^e^ or a radical surgical specimen)+CT	2	ITK-SNAP program (version 4.0.1; University of Pennsylvania)	196	137	Random sampling	59	LR^f^, LASSO^g^
Zhang et al (2021) [29]	China	Case-control	Multicenter	median（）Training set, median (IQR): 65 (56-72); Internal validation, median (IQR): 68 (61-74); External validation, median (IQR): 65 (59-77)	Training set: F: 75; M: 218; Internal validation: F: 13; M: 60; External validation: F: 13; M: 62	Muscle invasion status	CT	Pathology (TURBT or a radical surgical specimen)+CT	2	Deepwise Research Platform (Deepwise Inc)	Center 1: 366 Center 2: 75	293 (Development group+adjustment Group)	External validation	73+75 (Internal+external)	DL^h^
Ye et al (2023) [30]	China	Case-control	Multicenter	Training set, median (IQR): 67 (59-75); Internal validation, median (IQR): 69 (62-71); External validation, median (IQR): 64 (55-73)	Training set: F: 20; M: 109; Internal validation: F: 0; M: 30; External validation: F: 8; M: 47	Muscle invasion status	MRI^i^	Pathology (TURBT or a radical surgical specimen)+MRI	1	ITK-SNAP	Center 1: 160; Center 2: 55	—^j^	External validation	Internal validation: 25 (31 tumor lesions; MIBC^k^: 4) External validation: 54 (55 tumor lesions; MIBC: 32)	SVM^l^
Ren et al (2023) [31]	China	Case-control	Multicenter	Training set, median (IQR): 70.5 (37-90) Validation set, median (IQR): 72 (36-88)	Training set: F: 57; M: 131; Validation set: F: 35; M: 46	Muscle invasion status	CT	Pathology+within 30 days CT	1	ITK-SNAP	269	188	External validation	81	LR
Qureshi et al (2024) [32]	United States	Case-control	Multicenter	—	—	Muscle invasion status	MRI	Pathology+MRI	0	MATLAB (MATrix LABoratory; MathWorks)	—	—	External validation	—	NB^m^, SVM, DT^n^, KNN^o^, LR
Özdemir et al (2023) [33]	Türkiye	Case-control	Single center	—	—	Muscle invasion status	MRI	Pathology+MRI	1	—	60	—	—		LR
Chen et al (2022) [34]	China	Case-control	Single center	Training set, mean (SD): MIBC: 68.1250 (10.7065); NMIBC^p^: 66.7216 (12.9605) Validation set: MIBC: 62.2727 (8.7646); NMIBC: 64.4545 (12.5202)	Training set: MIBC: F: 26; M: 6; NMIBC: F: 78; M: 19 Validation set: MIBC: F: 11; M: 0; NMIBC: F: 26; M: 7	Muscle invasion status	CT	Pathology (TURBT or a radical surgical specimen) within+4 weeks MRI	2	ITK-SNAP	173	129	Random sampling	44	DL
Zheng et al (2021) [35]	China	Case-control	Single center	Training set, mean (SD): <65 years: 42 (33.6); ≥65 years: 83 (66.4); Validation set, mean (SD): <65 years: 24 (44.4); ≥65 years: 30 (55.6)	Training set: F: 19; M: 106 Validation set: F: 13; M: 41	Ki-67 expression	MRI	Pathology (TURBT or a radical surgical specimen)+MRI	2	ITK-SNAP	179	125	Random sampling	54	SMOTE-LASSO^q^
Ye et al (2023) [36]	China	Cohort study	Single center	Training set, mean (SD): 66.1 (11.2); Validation set: 66.1 (11.2)	Training set: F: 10; M: 54; Validation set: F: 5; M: 23	Histological grade	MRI	Pathology+MRI	2	ITK-SNAP	92	64	Random sampling	28	LR
Starmans et al (2022) [37]	Netherlands	Case-control	Multicenter	—	—	Lymph node staging	CT	Pathology+CT	1	WORC toolbox (Erasmus MC)	209	—	Cross validation	—	DL
Sarkar et al (2023) [38]	United States	Case-control	Single center	—	—	Histological grade	CT	—	2	—	100	—	—	—	NB
Li et al (2023) [39]	China	Case-control	Single center	—	—	Histological grade	MRI	—	2	ITK-SNAP	169	118	Random sampling	51	LR
Deng et al (2023) [40]	China	Case-control	Single center	Training set, mean (SD): low grade: 61.47 (11.34); high grade: 68.79 (9.87) Validation set, mean (SD): 72.09 (9.035)	Training set: low grade: F: 28; M: 1.75; high grade: F: 7; M: 36; Validation set: F: 7; M: 25	Histological grade	CT	Pathology+CT	2	Darwin Research Platform (Yizhun Medical AI)	105	73	Random sampling	32	SVM, KNN, GBDT^r^, LR, RF^s^, XGBoost^t^
Chen et al (2023) [41]	China	Case-control	Single center	—	—	Muscle invasion status	MRI, pathology	Pathology+MRI	2	Darwin Research Platform	445	312	Random sampling	133	SVM, KNN, Decision tree, RF, XGBoost, GBDT
Yu et al (2024) [42]	China	Case-control	Multicenter	—	—	Muscle invasion status	MRI, pathology	Pathology (TURBT or a radical surgical specimen)+MRI	2	—	436	404	External validation	32	DL
Xu et al (2019) [43]	China	Case-control	Single center	Total, mean (range): 66.1 (37-93); Training set, mean (range): 65.8 (38-86); Validation set, mean (range): 66.5 (37-93)	Total F: 49; M: 169 Training set: F: 27; M: 104 Validation set: F: 22; M: 65	Muscle invasion status	MRI	Pathology (TURBT or a radical surgical specimen)+MRI	2	ITK-SNAP	218	136	Random sampling	87	RF
Zhou et al (2019) [44]	China	Case-control	Single center	Mean (SD): 64.75 (4.74)	F: 42; M: 66	Histological grade	MRI	Pathology (TURBT)	2	ITK-SNAP	108	72	Random sampling	36	LR
Yang et al (2021) [45]	China	Case-control	Single center	Median (IQR): 68 (12)	F: 52; M: 317	Muscle invasion status	CT	Pathology (a radical surgical specimen)+CT	2	—	369	—	Random sampling	—	DL-CNN^u^
Zheng et al (2021) [46]	China	Case-control	Single center	Training set, n (%): <65 years: 48 (37.2); ≥65 years: 81 (62.8); Validation set, n (%): <65 years: 20 (35.7); ≥65 years: 36 (64.3)	Training set: F: 20; M: 109; Validation set: F: 12; M: 44	Muscle invasion status	MRI	Pathology+MRI	2	ITK-SNAP	185	129	Random sampling	56	LASSO, RF, SVM
Song et al (2023) [47]	China	Case-control	Multicenter	Training set, median (IQR): 66 (59-74) Validation set, median (IQR): 65 (58-72)	Training set: F: 469; M: 0 Validation set: F: 131; M: 44	Histological grade	CT	—	2	ITK-SNAP	688	469	External validation	219	LR, NB, SVM, KNN, RF, DT, XGBoost, LightGBM, GBDT, AdaBoost^v^, ANN^w^, DL
Cui et al (2022) [48]	China	Case-control	Single center	Training set, mean (SD): NMIBC: 66.2 (11.9); MIBC: 66.2 (11.7) Validation set, mean (SD): NMIBC: 65.4 (10.8); MIBC: 68.8 (9.3)	Training set: NMBIC: F: 16; M: 44; MBIC: F: 8: M: 52 Validation set: NMBIC: F: 7; M: 27; MBIC: F: 5; M: 29	Muscle invasion status	CT	Pathology+CT	2	ITK (Insight Software Consortium)	327	120	Random sampling	68	LR
Zhang et al (2020) [49]	China	Case-control	Single center	Training set, n (%): ≤60 years: 42 (38.9); >60 years: 66 (61.1) Validation set, n (%): ≤60 years: 14 (37.8); >60 years: 23 (62.2)	Training set: F: 28; M: 80 Validation set: F: 8; M: 29	Histological grade	CT	Pathology+CT	2	Deepwise Research Platform (Deepwise Healthcare)	145	108	Random sampling	37	LR
Wei et al (2023) [50]	China	Case-control	Multicenter	Training set, mean (SD): 65.86 (10.08) Validation set, mean (SD): 66.04 (9.56)	Training set: F: 17; M: 209 Validation set: F: 11; M: 86	Muscle invasion status	CT	Pathology+CT	2	ITK-SNAP	375	226	Random sampling	internal validation: 97; external validation: 52	DL
Zheng et al (2019) [51]	China	Case-control	Single center	Training set, median (IQR): 64 (57-69) Validation set, median (IQR): 61 (54-70)	Training set: F: 17; M: 113 Validation set: F: 9; M: 60	Muscle invasion status	MRI	Pathology+MRI	2	3D Slicer version 4.9.0.	169	130	Grouping by different periods	69	LR
Wang et al (2019) [52]	China	Case-control	Multicenter	Training set, mean (SD): 64.8 (10.6) Validation set, mean (SD): 62.9 (11.0)	Training set: F: 8; M: 56 Validation set: F: 7; M: 35	Muscle invasion status	MRI	Pathology+MRI	2	MATLAB 2016 (MathWorks)	106	64	External validation	42	LR
Zheng et al (2021) [53]	China	Case-control	Single center	Training set, n (%): <65 years: 48 (37.2); ≥65 years: 81 (62.8) Validation set, n (%): <65 years: 22 (39.3); ≥65 years: 34 (60.7)	Training set: F: 21; M: 108 Validation set: F: 11; M: 45	Histological grade	MRI	Pathology+MRI	2	ITK-SNAP	298	206	Random sampling	88	LASSO, RF, SVM
Wang et al (2022) [54]	China	Case-control	Single center	Total, median (IQR): 70 (62-76); Training set, median (IQR): 69 (62-76); Validation set, median (IQR): 70 (62-77); Test set, median (IQR): 69 (61-74)	Total: F: 45; M: 146; Training set: F: 22; M: 63; Validation set: F: 8; M: 28; Test set: F: 15; M: 55	Muscle invasion status	MRI	Pathology+MRI	2	ITK-SNAP	191	85	Random sampling	validation: 36; test: 70	LR
Yu et al (2023) [55]	China	Case-control	Single center	Training set (n): >65 years: 99; ≤65: 57; Validation set (n): >65 years: 19; ≤65 years: 20; Test set (n): >65 years: 25; ≤65 years: 28	training set: F: 19; M: 137 validation set: F: 9; M: 30; test set: F: 6; M: 37	HER 2 status	MRI	Pathology+MRI	2	ITK-SNAP	195	156	Random sampling	39	SVM, RF, LR, NB, KNN, AdaBoost
Wang et al (2023) [56]	China	Case-control	Single center	Total, median (IQR): 70 (62-77) Training set, median (IQR): 70 (64-77); Validation set, median (IQR): 69 (61-79)	Total: F: 55; M: 172 Training set: F: 35; M: 96 Validation set: F: 20; M: 76	Histological grade	MRI	Pathology+MRI	2	ITK-SNAP	227	131	Random sampling	96	LR
Zhang et al (2022) [57]	China	Case-control	Single center	Training set, mean (SD): 66 (11) Validation set, mean (SD): 67 (10) Test set, mean (SD): 66 (11)	Training set: F: 24; M: 215 Validation set: F: 9; M: 59; Test set: F: 5; M: 35	Muscle invasion status	MRI	Pathology+MRI	2	ITK-SNAP	342	239	Random sampling	Validation: 68; test: 35	LR
Zou et al (2022) [58]	China	Cohort study	Multicenter	Training set, median (range): 66 (26-95) Validation set, median (range): 68 (11-91) Retrospective, median (range): 64.5 (32-89) Prospective, median (range): 65 (47-91) Multicenter, median (range): 68 (42-89)	Training set: F: 37; M: 253 Validation set: F: 6; M: 60 Retrospective: F: 5; M: 29 Prospective: F: 7; M: 32 Multicenter: F: 7; M: 32	Muscle invasion status	MRI	Pathology+MRI	2	MBMIP model	468	290	Random sampling	178	DL
Zhou et al (2022) [59]	China	Case-control	Multicenter	—	—	Muscle invasion status	MRI	—	—	EvidentialNet	—	—	Random sampling	—	DL
Li et al (2023) [60]	China	Case-control	Multicenter	Training set, median (IQR): 67 (57-70) Test set, median (IQR): 63 (55-70)	Training set: F: 13; M: 80 Test set: F: 1; M: 27	Muscle invasion status	MRI	Pathology+MRI	1	ITK-SNAP	121	93	External validation	28	DL
Li et al (2024) [61]	China	Case-control	Single center	Training set, median (range): 66 (27-90) Validation set: 67 (27-90)	Training set: F: 33; M: 145; Validation set: F: 14; M: 63	Histological grade	MRI	Pathology+MRI	2	ITK-SNAP	255	178	Random sampling	77	LR
Chen et al (2022) [62]	China	Case-control	Single center	Training set, mean (SD): 69.85 (11.57) Validation set, mean (SD): 68.41 (11.17)	Training set: F: 36; M: 81 Validation set: F: 10; M: 41	Muscle invasion status	CT	Pathology+CT	4	ITK-SNAP	168	117	Random sampling	51	NB
Xu et al (2017) [63]	China	Case-control	Single center	—	—	Muscle invasion status	MRI	Pathology+MRI	2	MATLAB R2012b	68	—	Cross validation	—	SVM
Xu et al (2018) [64]	China	Case-control	Single center	—	—	Muscle invasion status	MRI	Pathology+MRI	2	MATLAB R2015b	54	—	Cross validation	—	SVM
Gao et al (2021) [65]	China	Case-control	Single center	—	—	Muscle invasion status	Ultrasound	Pathology+ultrasound	2	ITK-SNAP	157	110	Random sampling	47	NB
Wang et al (2019) [66]	China	Case-control	Single center	Total, mean (SD): 63.4 (10.4) Training set, mean (SD): 62.6 (11.0) Validation set, mean (SD): 65.2 (8.1)	Total: F: 14; M: 86 Training set: F: 11; M: 59 Validation set: F: 3; M: 27	Histological grade	MRI	Pathology+MRI	2		100	70	The time of undergoing surgery varies.	30	LR
Zhang et al (2017) [67]	China	Case-control	Single center	—	—	Histological grade	MRI	Pathology+MRI	2	MATLAB R2012b	—	61	—	—	SVM
Gresser et al (2022) [68]	Germany	Case-control	Single center	Training set, mean (SD): PN1-2: 68 (11); PN0: 68 (10) Validation set, mean (SD): PN1-2: 70 (9); PN0: 69 (11)	Training set: PN1-2: F: 24, M: 42; PN0: F: 57; M: 151 Validation set: F: 9, M: 19; PN0: F: 16; M: 73	Lymph node staging	CT	Pathology (a radical surgical specimen)+MRI	2	Medical Imaging Interaction Toolkit (MITK, DKFZ, Heidelberg; version 2018.04.2)	391	274	Random sampling	117	ANN
Tong et al (2018) [69]	United States	Case-control	Single center	Mean (SD): 65.6 (10.5)	F: 18; M: 47	Muscle invasion status	MRI	Pathology+MRI	2	—	65	—	Leave-one-out method	—	SVM
Tao et al (2024) [70]	China	Case-control	Single center	Median (IQR): 65 (30-86)	F: 33; M: 105	Histological grade	MRI	Pathology+MRI	—	3D Slicer (version 4.11.20210226)	138	110	Random sampling	28	DL
Liu et al (2022) [71]	China	Case-control	Single center	Training set: 62.78 (11.65); Validation set: 64.41 (10.10)	Training set: F: 19; M: 146 Validation set: F: 7; M: 34	Muscle invasion status	MRI	Pathology+MRI	2	Manually depict	206	165	Cross validation	28	LASSO
Liu et al (2022) [72]	China	Case-control	Single center	—	—	Histological grade	CT	Pathology+CT	2	MSRN network (Multi-Scale Residual Network)	75	51	Random sampling	24	DL
Razik et al (2021) [73]	India	Case-control	Single center	Mean (SD): 57.6 (11.8)	F: 5; M: 35	Histological grade	MRI	Pathology+MRI	2	TexRAD (Feedback Medical)	40	—	—	—	LR
Peng et al (2024) [74]	China	Case-control	Single center	Training set, mean (SD): HER2^x^ positive: 64.64 (11.56); HER2 negative: 64.24 (12.78) Validation set, mean (SD): HER2 positive: 59.71 (13.60); HER2 negative: 64.50 (7.89)	—	HER2 status	CT	Pathology+CT	2	ITK-SNAP	124	100	Random sampling	24	SVM, RF, LR, MLP^y^, ExtraTrees, LightGBM^z^, XGBoost, KNN
Xiong et al (2024) [75]	China	Case-control	Single center	Training set, median (IQR): 69.00 (63.00-75.00) Validation set, median (IQR): 68.50 (57.25-76.00)	Training set: F: 8; M: 65 Validation set: F: 8; M: 24	Muscle invasion status	CT	Pathology+CT	2	Darwin Research Platform	105	73	Random sampling	32	GBDT, K-NN, LR, RF, SVM, (XGBoost
Feng et al (2024) [76]	China	Case-control	Single center	Mean (SD): 66.16 (12.67)	F: 27; M: 108	Ki-67 expression	CT	Pathology+CT	2	3D Slicer (version: 4.10.2)	135	94	Random sampling	41	LR
Wei et al (2024) [77]	China	Case-control	Multicenter	Total, mean (SD): 67.52 (10.32); Training set, mean (SD): 67.41 (9.80) Validation set, mean (SD): 67.85 (11.80)	Total: F: 22; M: 185 Training set: F: 16; M: 138 Validation set: F: 6; M: 47	HER2 status	CT	Pathology+CT	2	ITK-SNAP	207	154	External validation	53	LR, SVM, KNN, RF, XGBoost
Cai et al (2025) [78]	China	Case-control	Multicenter	Training set, mean (SD): 66 (12) Validation set, mean (SD): 67 (13) Internal test set, mean (SD): 67 (11); external test set: 69 (10)	Training set: F: 37; M: 254 Validation set: F: 6; M: 60 Internal test set: F: 25; M: 139 External test set: F: 6; M: 32	Muscle invasion status	MRI	Pathology+MRI	2	DL	559	291	External validation	Validation set: 66; internal test set: 164, external test set: 38	DL, VI-RADS
Du et al (2025) [79]	China	Case-control	Single center	Training set, mean (SD): NMBIC: 66.47 (9.22); MBIC: 66.47 (9.22) Validation set, mean (SD): NMBIC: 67.27 (10.75); MBIC: 68.62 (8.45)	Training set: NMBIC: F: 27; M: 70; MBIC: F: 12; M: 31 Validation set: NMBIC: F: 10; M: 34; MBIC: F: 6; M: 10	Muscle invasion status	CT	Pathology+CT	2	ITK-SNAP (v.3.8.0)	200	140	Random sampling	60	DL
Du et al (2025) [80]	China	Case-control	Multicenter	Training set: total: 69.17 (9.92); NMBIC: 69.12 (9.51); MBIC: 69.42 (11.95) Validation set: total: 68.03 (12.03); NMBIC: 67.90 (12.35); MBIC: 68.69 (10.66)	Training set: total: F: 51; M: 180; NMBIC: F: 47; M: 146; MBIC: F: 4; M: 34 Validation set: total: F: 22; M: 72; NMBIC: F: 20; M: 58; MBIC: F: 2; M: 14	Muscle invasion status	CT	Pathology+CT	2	ITK-SNAP	325	231	External validation	94	Extra tree
He et al (2025) [81]	China	Case-control	Multicenter	Training set, (n): ≤60 years: 48; >60 years: 70 Test set (n): ≤60 years: 11; >60 years: 19 External test set: ≤60 years: 24; >60 years: 39	Training set: F: 29; M: 89 Test set: F: 8; M: 22 External test set: F: 21; M: 42	Muscle invasion status	CT	Pathology+CT	1	Manual	211	118	External validation	Test set: 30; external validation: 63	LR, SVC (linear), SVC (poly), SVC (rbf)
Hu et al (2025) [82]	China	Case-control	Single center	Training set, mean (SD): NMBIC: 65.92 (9.83); MBIC: 68.55 (9.18) Validation set, mean (SD): NMBIC: 67.40 (8.37); MBIC: 70.53 (7.67)	Training set: NMBIC: F: 16; M: 83; MBIC: F: 5; M: 37 Validation set: NMBIC: F: 4; M: 38; MBIC: F: 7; M: 12	Muscle invasion status	CT	Pathology+CT	2	ITK-SNAP (version 3.8.0)	202	141	Random sampling	61	DECT^aa^, Radiomacs, Nomogram
Yu et al (2025) [83]	China	Case-control	Multicenter	—	—	Muscle invasion status	MRI	Pathology+MRI	4	Manual	401	313	External validation	Validation set: 26; internal test set: 34; external test set: 28	DL
Zhou et al (2025) [84]	China	Case-control	Single center	LGUC^ab^, mean (SD): 61.98 (12.29); HGUC^ac^, mean (SD): 68.85 (11.91)	L: F: 41; M: 141 H: F: 30; M: 160	Histological grade	CT	Pathology+CT	2	3D Slicer image	372	259	Random sampling	LR, DT, SVM, AdaBoost	LR, DT, SVM, AdaBoost

a ROI: region of interest.

b F: female.

c M: male.

d CT: computed tomography.

e TUBRT: transurethral resection of bladder tumor.

f LR: logistic regression.

g LASSO: least absolute shrinkage and selection operator.

h DL: deep learning.

i MRI: magnetic resonance imaging.

j Not available.

k MIBC: muscle-invasive bladder cancer.

l SVM: support vector machine.

m NB: naive Bayes.

n DT: decision tree.

o KNN: k-nearest neighbors.

p NMIBC: non–muscle-invasive bladder cancer.

q SMOTE-LASSO: Synthetic Minority Over-sampling Technique–Least Absolute Shrinkage and Selection Operator.

r GBDT: gradient boosting decision tree.

s RF: random forest.

t XGBoost: extreme gradient boosting.

u DL-CNN: deep learning–convolutional neural network.

v AdaBoost: adaptive boosting.

w ANN: artificial neural network.

x HER-2: human epidermal growth factor receptor 2.

y MLP: multilayer perceptron.

z LightGBM: light gradient boosting machine.

aa DECT: dual-energy computed tomography.

ab LGUC：low-grade urothelial carcinoma

ac HGUC: high-grade urothelial carcinoma

### Evaluation of ROB and Quality

#### PROBAST-AI

**Figure 2.**
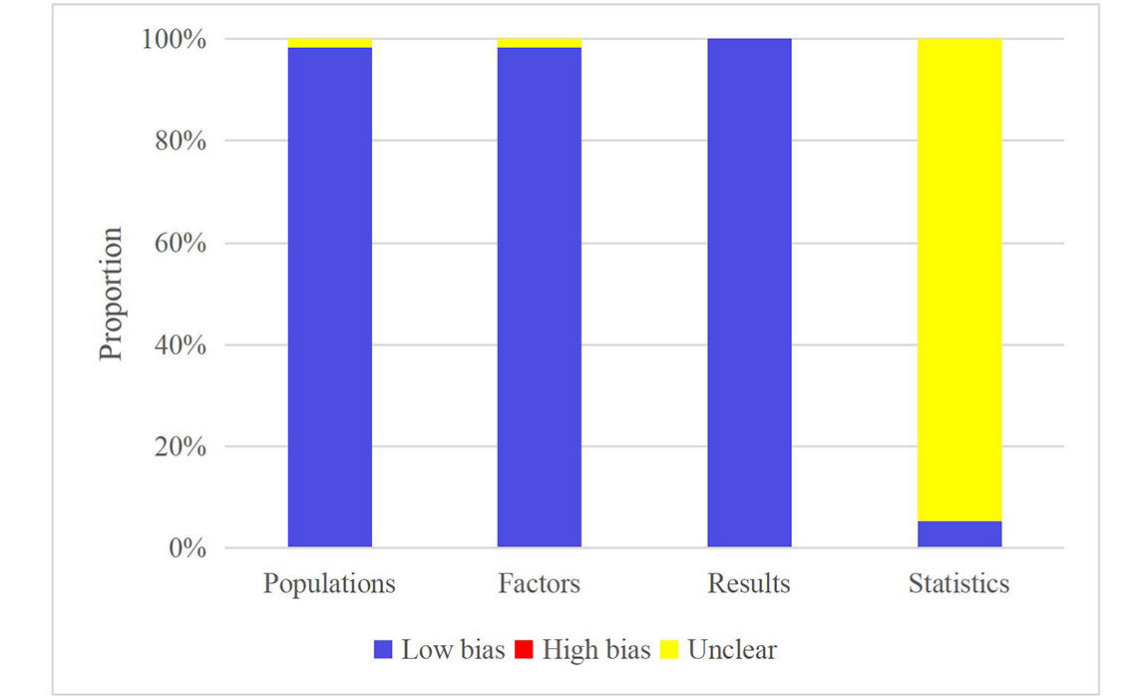
Risk of bias assessment results for model development.

Regarding the model development, all studies exhibited low ROB across all signaling questions in the “Participants” domain, with the sole exception of the question “Did the in- and exclusions of study participants result in a representative data set?” for which all studies were rated as “unclear.” For “Predictor” assessments, all included investigations indicated an uncertain risk pertaining to the question “Were predictor assessments made without knowledge of outcome data?” In the “Outcomes” domain, all studies demonstrated low ROB. Within the “Analysis” segment, uncertainty was noted in 40 [29,30,32-34,36-38,40-45,47-50,55-57,59,60,62-65,67,69-73,77-79,81-84] investigations concerning “Was there evidence that the sample size was reasonable?” Two publications [32,35] also flagged uncertainty regarding “Were participants with missing or censored data handled appropriately in the analysis?” Furthermore, 21 [29,30,32,34,35,39,45,53,57-60,67,68,71-73,79-81,83] studies presented uncertainty on the item “If methods to address class imbalance were used, was the model or the model predictions recalibrated?” Forty-one [28-32,34-43,45-55,58-62,65-70,72,73,78,79] investigations indicated uncertainty for “Were methods used to address potential model overfitting?” (Figure 2).

**Figure 3.**
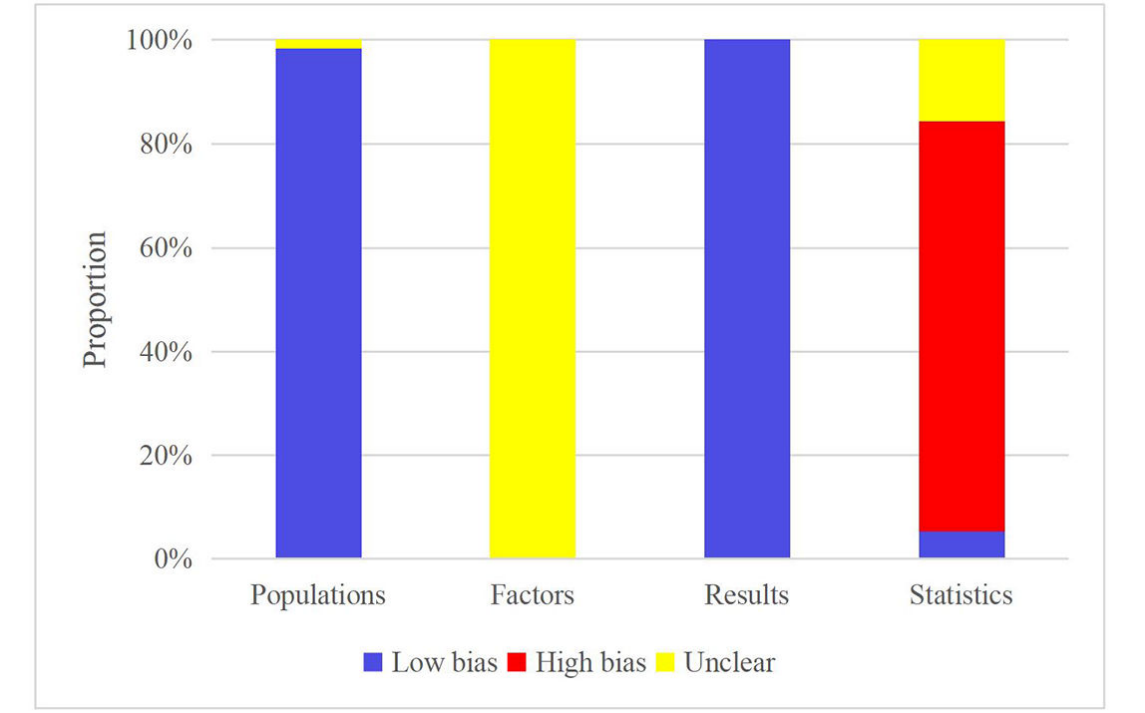
Risk of bias assessment results for model evaluation.

During model evaluation, all but one [33] study demonstrated low ROB in the “Participants” domain. The exception was an unclear rating for the question “Did the in- and exclusions of study participants result in a representative data set?” For the “Predictors” domain, all studies were uniformly rated as unclear regarding “Were predictor assessments made without knowledge of outcome data?” The “Outcome” domain was consistently judged as low ROB across all studies. In the “Analysis” domain, 4 [33,38,67,73] studies were identified with a high ROB for “Was model evaluation based on only apparent performance avoided?” precluding further analysis. Forty-one [28,30-32,34-36,39,40,42-46,48-53,55,57,59-66,69,71,72,74-77,79,80,82,83] publications indicated a high ROB regarding “Was there evidence that the sample size was reasonable?” Additionally, 37 [28-32,34-36,39-43,45-55,58-62,65,66,68-70,72,78,79] studies reported an unclear risk for “If resampling methods were used to evaluate model performance, were all model development steps replicated in the resampling process?” (Figure 3).

In summary, while the model development generally demonstrated a low ROB across participants and data sources, predictors, outcome, and analysis, the model evaluation phase presented a higher ROB specifically within the analysis dimension, particularly concerning the criteria “Was model evaluation based on only apparent performance avoided?” and “Was there evidence that the sample size was reasonable?”

#### RQS

Two [32,38] studies did not provide complete imaging protocols. Multiple image segmentation was not performed in 7 [30-33,37,60,81] studies. All 57 studies failed to address scanner variability and vendor-dependent features, as well as imaging across multiple timepoints (ie, collecting individual images at different times). Three [32,37,69] studies did not use multiple testing correction or feature reduction to mitigate overfitting risk, nor did they use different data reduction methods (eg, principal component analysis and least absolute shrinkage and selection operator) for cross-analysis to reduce overfitting. Forty-two [29-31,33-38,40-50,55,56,58,60,62-65,67,69-73,75,78-84] studies did not perform multivariate analysis incorporating nonradiomic features. In all 57 [28-84] studies, biological correlations were neither detected nor discussed. The demonstration of phenotypic differences (potentially related to underlying gene-protein expression patterns) enhances the understanding of biology and radiomics. A cutoff value for disease presence or prediction risk was not provided in 51 [29,30,32-34,36-38,40-51,53-61,63-84] studies. Discrimination statistics were absent in 2 [32,69] studies. The reporting of these statistics (eg, ROC curve, C-statistic, and area under the curve [AUC]) and related statistical significance (eg, *P* values and CIs), or the application of resampling methods (eg, cross-validation and bootstrapping) was not performed. Calibration statistics were not reported in 47 [29-38,42-45,48-50,52-78,80,82,83] studies. Neither calibration statistics (eg, calibration plots, calibration in the large or slope) nor related statistical significance (eg, *P* values and CIs), or the application of resampling methods (eg, cross-validation and bootstrapping) were reported. Fifty-six [28-36,38-84] studies were not prospective studies registered in trial databases. Validation was lacking in 4 [33,38,67,73] studies. Comparisons with a “gold standard”—assessing the model’s agreement with the “gold standard” methods—were not performed in all 57 studies. Potential clinical utility—reporting potential and the applications of the model in a clinical setting (eg, decision curve analysis)—was not addressed in 37 [30,32,33,36-39,42-45,48,49,54-73,78,80,82,83] studies. Cost-effectiveness analysis—reporting the cost-effectiveness of clinical application (eg, clinical impact curve)—was not performed in 56 [28-38,40-84] studies. Only 3 [28,32,35] studies did not adhere to open science and data principles by failing to release code and data. Regarding the RQS, a penalty of 3 points was applied for the absence of measures to control overfitting, and a deduction of 5 points was incurred for the lack of a validation set. Conversely, a bonus of 7 points was awarded to studies that were prospectively registered in a trial database. Among the 57 eligible investigations, 4 lacked any validation set. External validation was implemented in 16 [29-32,42,47,50,52,58-60,77,78,80,81,83] studies. Prospective registration was documented in only a single [37] study. The average quality score was 11.60 (range 1-17, IQR 3.5), representing an average percentage of 32.21% (Table S1 in Multimedia Appendix 2).

### Meta-Analysis

#### Meta-Analysis for AUROC for Identifying Muscle Invasion

**Figure 4.**
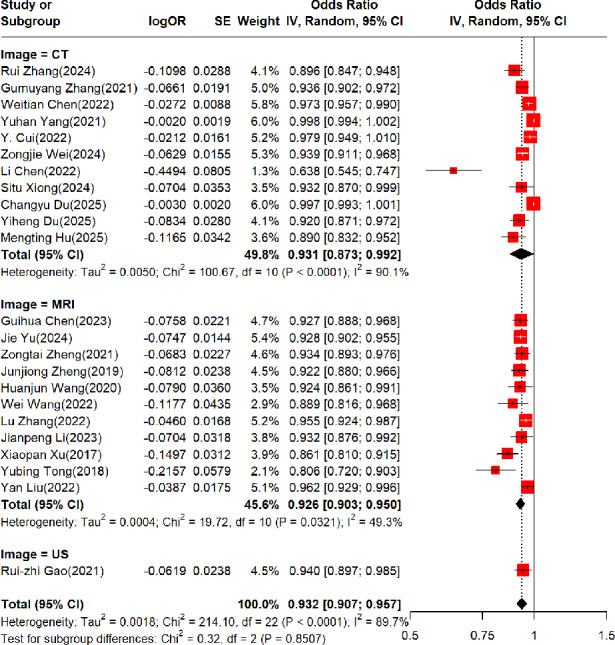
Summary plot of radiomics for detecting muscle invasion in the training set [28,29,34,41,42,45,46,48,50-52,54,57,60,62,63,65,69,71,75,79,80,82]. For the 3 studies that included both radiomics-based and combined radiomics-clinical models, only the best-performing model in the validation set was retained to ensure independence across studies.

The meta-analysis of AUROC was conducted using a random-effects model. In the training sets, the pooled AUROC for radiomics-based ML was 0.932 (95% CI 0.907-0.957; grade: moderate Figure 4 [28,29,34,41,42,45,46,48,50-52,54,57,60,62,63,65,69,71,75,79,80,82]).

The pooled AUROC was 0.932 (95% CI 0.853-1.000; grade: weak), 0.907 (95% CI 0.863-0.954; grade: weak), and 0.940 (95% CI 0.897-0.985; grade: weak) for CT-, MRI-, and ultrasound-based radiomics, respectively (Table 2; Figures S1 and S2 in Multimedia Appendix 3). The pooled AUROC was 0.914 (95% CI 0.871-0.959; grade: weak) and 0.934 (95% CI 0.908-0.960; grade: moderate) for models integrating clinical features with CT- or MRI-based radiomics (Table 2; Figures S3 and S4 in Multimedia Appendix 3).

**Table 2.** Meta-analysis results for the identification of area under the receiver operating characteristic curve (AUROC) in muscle invasion.

Subgroup	Training set						Validation set					
	Number of models	AUROC^a^ (95% CI)	tau^2^	tau	*I* ^2^	Grade^b^	Number of models	AUROC (95% CI)	tau^2^	tau	*I* ^2^	Grade
Radiomics												
CT^c^												
Extra trees							1	0.825 (0.712-0.956)				⊕⊕ѲѲ
ANN^d^							1	0.950 (0.896-1.000)				⊕⊕ѲѲ
DL^e^	6	0.970 (0.940-1.000)	0.0007	0.0269	86	⊕⊕⊕Ѳ	5	0.903 (0.826-0.988)	0.0047	0.0687	88.1	⊕⊕ѲѲ
LR^f^	2	0.938 (0.513-1.000)	0.0038	0.0619	84.3	⊕ѲѲѲ	2	0.891 (0.842-0.942)	0	0	0	⊕⊕ѲѲ
NB^g^	1	0.638 (0.545-0.747)				⊕ѲѲѲ	1	0.665 (0.522-0.847)	——			⊕ѲѲѲ
Overall	9	0.932 (0.853-1.000)	0.0083	0.0914	89.8	⊕⊕ѲѲ	10	0.893 (0.840-0.948)	0.004	0.0634	85.4	⊕⊕ѲѲ
MRI^h^												
DL	2	0.929 (0.910-0.948)	0	0	0	⊕⊕⊕Ѳ	5	0.908 (0.892-0.924)	0	0	0	⊕⊕ѲѲ
LASSO^i^	2	0.951 (0.793-1.000)	<0.0001	0.005	5.8	⊕⊕ѲѲ	2	0.906 (0.900-0.913)	0	0	0	⊕⊕⊕Ѳ
LR	1	0.933 (0.895-0.972)				⊕⊕ѲѲ	1	0.931 (0.847-1.000)				⊕⊕ѲѲ
RF^j^							1	0.907 (0.856-0.961)				⊕⊕ѲѲ
SVM^k^	3	0.840 (0.772~0.914)	0	0	0	⊕⊕ѲѲ	2	0.913 (0.299-1.000)	0.0132	0.1151	85.4	⊕ѲѲѲ
Overall	8	0.907 (0.863-0.954)	0.0022	0.047	71	⊕⊕ѲѲ	11	0.916 (0.891-0.942)	0.0001	0.01	0	⊕⊕ѲѲ
Ultrasonography	1	0.940 (0.897--0.985)				⊕⊕ѲѲ	1	0.840 (0.737-0.958)				⊕⊕ѲѲ
Radiomics+clinical features												
CT	3	0.914 (0.871-0.959)	0	0	0	⊕⊕ѲѲ	4	0.874 (0.852-0.896)	0	0	0	⊕⊕ѲѲ
MRI	5	0.934 (0.908-0.960)	<0.0001	0.0077	0	⊕⊕⊕Ѳ	5	0.921 (0.867-0.979)	0.0005	0.0228	19.8	⊕⊕ѲѲ

a AUROC: area under the receiver operating characteristic curve.

b In the GRADE assessment section, ⊕⊕⊕⊕ represents high quality of evidence, ⊕⊕⊕Ѳ represents moderate quality of evidence, ⊕⊕ѲѲ represents low quality of evidence, and ⊕ѲѲѲ represents very low quality of evidence.

c CT: computed tomography.

d ANN: artificial neural network.

e DL: deep learning.

f LR: logistic regression.

g NB: naive Bayes.

h MRI: magnetic resonance imaging.

i LASSO: least absolute shrinkage and selection operator.

j RF: random forest.

k SVM: support vector machine.

**Figure 5.**
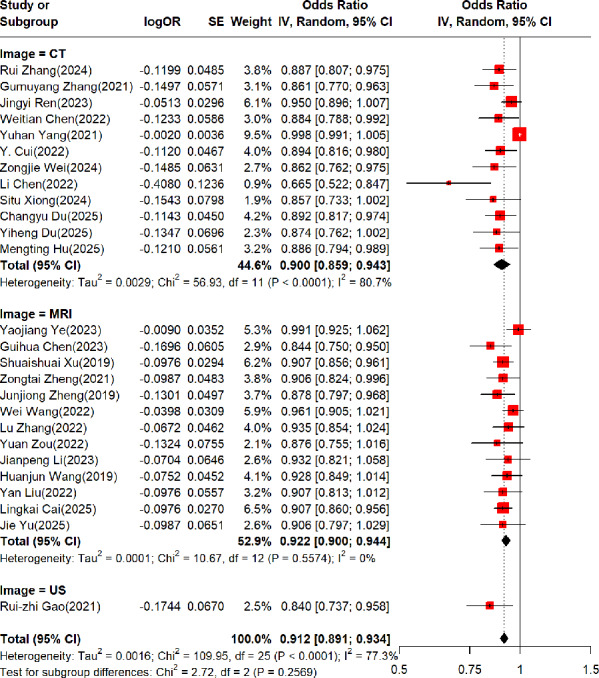
Summary plot of radiomics for detecting muscle invasion in the validation set [28-31,34,41,43,45,46,48,50,51,54,57,58,60,62,65,66,69,75,78-80,82,83].

In the validation sets, the pooled AUROC for radiomics-based ML was 0.912 (95% CI 0.891-0.934; grade: weak Figure 5 [28-31,34,41,43,45,46,48,50,51,54,57,58,60,62,65,66,69,75,78-80,82,83]).

The pooled AUROC was 0.893 (95% CI 0.840-0.948; grade: weak), 0.916 (95% CI 0.891-0.942; grade: weak), and 0.840 (95% CI 0.737-0.958; grade: weak) for CT-, MRI-, and ultrasound-based radiomics. The pooled AUROC was 0.874 (95% CI 0.852-0.896; grade: weak) and 0.921 (95% CI 0.867-0.979; grade: weak) for models integrating clinical features with CT- or MRI-based radiomics (Table 2 and Figures S5-S8 in Multimedia Appendix 3).

#### Meta-Analysis for Sensitivity and Specificity in Muscle Invasion Identification

The meta-analysis of 2x2 diagnostic contingency tables was conducted via a bivariate mixed effects model. In the training sets, the pooled sensitivity and specificity were 0.92 (95% CI 0.84-0.96) and 0.88 (95% CI 0.83-0.92), 0.80 (95% CI 0.76-0.84) and 0.90 (95% CI 0.86-0.92), and 0.81 and 0.91 for CT-, MRI-, and ultrasound-based radiomics. The pooled sensitivity and specificity were 0.79‐0.93, 0.58-0.92, and 0.86 (95% CI 0.79-0.91) and 0.90 (95% CI 0.86-0.92) for models integrating clinical features with CT- or MRI-based radiomics (Table 3).

In the validation sets, the pooled sensitivity and specificity were 0.87 (95% CI 0.80-0.91) and 0.83 (95% CI 0.76-0.88), and 0.87 (95% CI 0.79-0.92) and 0.89 (95% CI 0.84-0.92) for CT- and MRI-based radiomics. The pooled sensitivity and specificity were 0.86 (95% CI 0.77-0.92) and 0.75 (95% CI 0.64-0.84) for models integrating clinical features with CT-based radiomics, and 0.88 (95% CI 0.81-0.93) and 0.88 (95% CI 0.76-0.94) for models integrating clinical features with MRI-based radiomics (Table 3).

**Table 3.** Meta-analysis results for sensitivity and specificity in the identification of muscle invasion.

Subgroup	Training set			Validation set		
	Number of models	Sensitivity (95% CI)	Specificity (95% CI)	Number of models	Sensitivity (95% CI)	Specificity (95% CI)
Radiomics						
CT^a^						
Extra trees	1	0.82	0.81	1	0.81	0.8
ANN^b^				1	0.9	0.93
DL^c^	4	0.95 (0.85-0.99)	0.89 (0.82-0.93)	5	0.88 (0.77-0.94)	0.83 (0.71-0.91)
LR^d^	2	0.76‐0.95	0.86‐0.95	3	0.74‐0.95	0.72‐0.88
Overall	7	0.92 (0.84-0.96)	0.88 (0.83-0.92)	10	0.87 (0.80-0.91)	0.83 (0.76-0.88)
MRI^e^						
DL	2	0.75‐0.90	0.87‐0.93	4	0.87 (0.78-0.92)	0.93 (0.88-0.96)
LASSO^f^	2	0.80‐0.92	0.93‐0.90	2	0.92‐0.94	0.78‐0.87
LR	2	0.82‐0.83	0.84‐0.90	1	0.81	0.83
RF^g^				1	0.87	0.78
SVM^h^	2	0.74‐0.80	0.75‐0.82	3	0.67‐1	0.88‐0.96
Overall	8	0.80 (0.76-0.84)	0.90 (0.86-0.92)	11	0.87 (0.79-0.92)	0.89 (0.84-0.92)
Ultrasound	1	0.81	0.91	—^i^	—	—
Radiomics+ Clinical features						
CT						
Overall	3	0.79‐0.93	0.58‐0.92	4	0.86 (0.77-0.92)	0.75 (0.64-0.84)
MRI						
LR	4	0.86 (0.79-0.91)	0.90 (0.86-0.92)	5	0.88 (0.78-0.94)	0.91 (0.79-0.96)
Overall	4	0.86 (0.79-0.91)	0.90 (0.86-0.92)	6	0.88 (0.81-0.93)	0.88 (0.76-0.94)

a CT: computed tomography.

b ANN: artificial neural network.

c DL: deep learning.

d LR: logistic regression.

e MRI: magnetic resonance imaging.

f LASSO: least absolute shrinkage and selection operator.

g RF: random forest.

h SVM: support vector machine.

i Not available.

### Subgroup Analysis

Subgroup analyses of AUROC for the diagnosis of muscle invasion were performed based on different imaging modalities and model types. Among CT-based radiomics models, deep learning (DL) was the predominant approach. In the validation sets, the pooled AUROC of DL was 0.903 (95% CI 0.826-0.988; grade: weak), with a sensitivity of 0.88 (95% CI 0.77-0.94) and a specificity of 0.83 (95% CI 0.71-0.91). Among MRI-based radiomics models, DL was the most common modeling approach. In the validation sets, the pooled AUROC of DL was 0.908 (95% CI 0.892-0.924; grade: weak), with a sensitivity of 0.87 (95% CI 0.78-0.92) and specificity of 0.93 (95% CI 0.88-0.96). In the validation sets, the model integrating CT-based radiomics with clinical characteristics yielded an AUROC of 0.874 (95% CI 0.852-0.896; grade: weak). The corresponding sensitivity was 0.86 (95% CI 0.77-0.92), and the specificity was 0.75 (95% CI 0.64-0.84). Conversely, for MRI-based models combined with clinical variables, the pooled AUROC was 0.921 (95% CI 0.867-0.979; grade: weak). The corresponding sensitivity and specificity were 0.88 (95% CI 0.81-0.93) and 0.88 (95% CI 0.76-0.94), respectively (Tables 2-3).

### Diagnosis of High-Grade Tumors

#### Pooled Results

**Figure 6.**
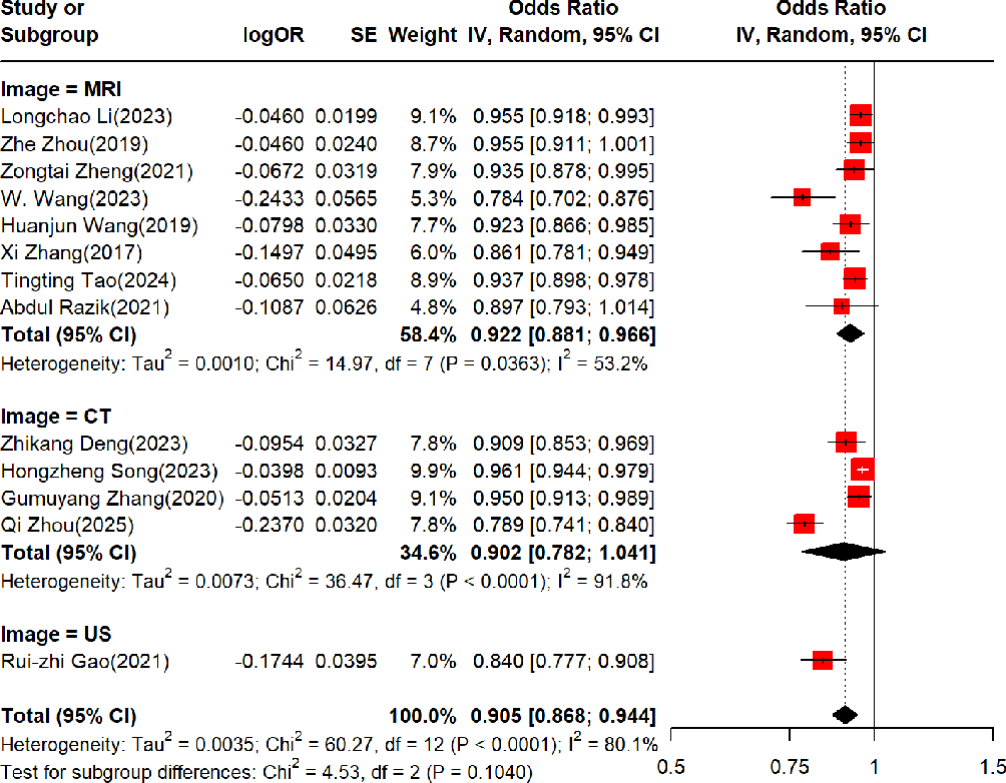
Summary plot of radiomics for identifying high-grade tumors in the training set [39,40,44,47,49,53,56,65-67,70,73,84].

A meta-analysis of AUROC was conducted using a random-effects model. In the training sets, the pooled AUROC for radiomics-based ML was 0.905 (95% CI 0.868-0.944; grade: weak; Figure 6 [39,40,44,47,49,53,56,65-67,70,73,84]).

The pooled AUROC was 0.902 (95% CI 0.782-1.000; grade: weak) for CT-based radiomics, 0.895 (95% CI 0.814-0.985; grade: weak) for MRI-based radiomics, and 0.840 (95% CI 0.777-0.908; grade: weak) for ultrasound-based radiomics (Figures S9 and S10 in Multimedia Appendix 3 and Table 3). The AUROC for MRI-based radiomics combined with clinical features was 0.944 (95% CI 0.904-0.985; grade: moderate; Figure S11 in Multimedia Appendix 3).

**Figure 7.**
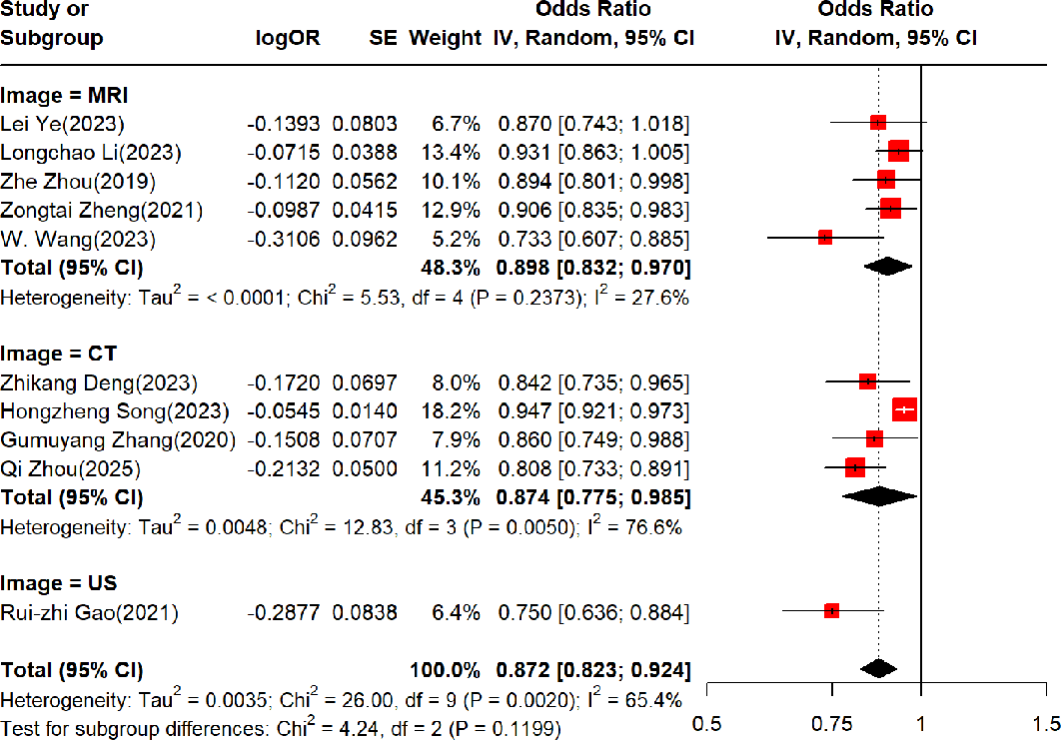
Summary plot of radiomics for identifying high-grade tumors in the validation set [36,39,40,44,47,49,53,56,65,84].

In the validation sets, the pooled AUROC for radiomics-based ML was 0.872 (95% CI 0.823-0.924; grade: weak; Figure 7 [36,39,40,44,47,49,53,56,65,84]).

The pooled AUROC was 0.874 (95% CI 0.775-0.985; grade: weak) for CT-based radiomics, 0.846 (95% CI 0.663-1.000; grade: very low) for MRI-based radiomics, and 0.750 (95% CI 0.636-0.884; grade: very low) for ultrasound-based radiomics. (Figures S12-S13 in Multimedia Appendix 3). The AUROC for MRI-based radiomics combined with clinical features was 0.919 (95% CI 0.774-1.000; grade: weak; Table 4 and Figure S14 in Multimedia Appendix 3).

**Table 4.** Meta-analysis results for area under the receiver operating characteristic curve (AUROC) in the identification of high-grade tumors.

Subgroup	Training set						Validation set					
	No. of models	AUROC (95% CI)	tau^2^	tau	*I* ^2^	Grade^a^	No. of models	AUROC (95% CI)	tau^2^	tau	*I* ^2^	Grade
Radiomics												
CT^b^												
DL^c^	1	0.961 (0.944-0.979)				⊕⊕⊕Ѳ	1	0.947 (0.921-0.973)				⊕⊕⊕Ѳ
LR^d^	1	0.950 (0.913-0.989)				⊕⊕⊕Ѳ	1	0.860 (0.749-0.988)				⊕⊕ѲѲ
SVM^e^	1	0.909 (0.853-0.969)				⊕⊕ѲѲ	2	0.819 (0.639-1.000)	0	0	0	⊕ѲѲѲ
AdaBoost	1	0.789 (0.7410.840)				⊕⊕ѲѲ						
Overall	4	0.902 (0.782-1.000)	0.0073	0.0854	91.8	⊕⊕ѲѲ	4	0.874 (0.7750.985)	0.0048	0.0689	76.6	⊕⊕ѲѲ
MRI^f^												
DL	1	0.937 (0.898-0.978)				⊕⊕ѲѲ						
LR	3	0.882 (0.685-1.000)	0.0084	0.0915	81.1	⊕ѲѲѲ	3	0.846 (0.663-1.000)	0.0031	0.0555	38.3	⊕ѲѲѲ
SVM	1	0.861 (0.781-0.949)				⊕⊕ѲѲ						
Overall	5	0.895 (0.814-0.985)	0.004	0.0633	69.3	⊕⊕ѲѲ	3	0.846 (0.663-1.000)	0.0031	0.0555	38.3	⊕ѲѲѲ
Ultrasound	1	0.840 (0.777-0.908)				⊕⊕ѲѲ	1	0.750 (0.636-0.884)			NA	⊕ѲѲѲ
Radiomics+clinical features												
CT												
MRI	3	0.944 (0.904-0.985)	0	0	0	⊕⊕⊕Ѳ	2	0.919 (0.774-1.000)	0	0	0	⊕⊕ѲѲ

a In the GRADE assessment section, ⊕⊕⊕⊕ represents high quality of evidence, ⊕⊕⊕Ѳ represents moderate quality of evidence, ⊕⊕ѲѲ represents low quality of evidence, and ⊕ѲѲѲ represents very low quality of evidence.

b CT: computed tomography.

c DL: deep learning.

d LR: logistic regression.

e SVM: support vector machine.

f MRI: magnetic resonance imaging.

A meta-analysis of 2×2 diagnostic contingency tables was performed via a bivariate mixed-effects model. In the training sets, the pooled sensitivity and specificity were 0.91 (95% CI 0.84-0.95) and 0.74 (95% CI 0.67-0.79) for CT-based radiomics, and 0.86 (95% CI 0.57-0.96) and 0.89 (95% CI 0.83-0.93) for MRI-based radiomics. The sensitivity and specificity were 0.78 (95% CI 0.65-0.87) and 0.94 (95% CI 0.79-0.99) for MRI-based radiomics combined with clinical features.

In the validation sets, the pooled sensitivity and specificity were 0.65‐0.95 and 0.69‐0.73 for CT-based radiomics, and 0.81~0.89 and 0.70~0.91 for MRI-based radiomics. The sensitivity and specificity were 0.54‐0.77 and 0.88‐0.98 for MRI-based radiomics combined with clinical features (Table 5).

**Table 5.** Meta-analysis results for sensitivity and specificity in identification of high-grade tumors.

Subgroup	Training set			Validation set		
	Number of models	Sensitivity (95% CI)	Specificity (95% CI)	Number of models	Sensitivity (95% CI)	Specificity (95% CI)
Radiomics						
CT^a^						
LR^b^	1	0.96	0.71	1	0.89	0.73
NB^c^	1	0.89	0.77			
SVM^d^	1	0.93	0.77	2	0.65‐0.95	0.69‐0.72
AdaBoost	1	0.85	0.73			
Overall	4	0.91 (0.84-0.95)	0.74 (0.67-0.79)	3	0.65‐0.95	0.69‐0.73
MRI^e^						
DL^f^	1	0.99	0.9			
LR	2	0.60‐0.74	0.82‐1	3	0.81‐0.89	0.70‐0.91
SVM	1	0.78	0.87			
XGBoost						
Overall	4	0.86 (0.57-0.96)	0.89 (0.83-0.93)	3	0.81‐0.89	0.70‐0.91
Ultrasound	1	0.8	0.76			
Radiomics+ Clinical features						
MRI						
Overall	4	0.78 (0.65-0.87)	0.94 (0.79-0.99)	2	0.54‐0.77	0.88‐0.98

a CT: computed tomography.

b LR: logistic regression.

c NB: naive Bayes.

d SVM: support vector machine.

e MRI: magnetic resonance imaging.

f DL: deep learning.

#### Subgroup Analysis

Subgroup analyses of AUROC for the diagnosis of high-grade tumors were performed based on different imaging modalities and model types. In CT-based radiomics, the support vector machine (SVM) model was the predominant type. The pooled AUROC of SVM was 0.819 (95% CI 0.639-1.000; grade: very low) in the validation sets, with corresponding sensitivity of 0.65‐0.95 and specificity of 0.69‐0.72. Likewise, the LR model was the predominant type in MRI-based radiomics. The pooled AUROC of LR was 0.846 (95% CI 0.663-1.000; grade: very low) in the validation sets, with corresponding sensitivity of 0.81-0.89 and specificity of 0.70-0.91. In the validation sets, the model integrating MRI-based radiomics with clinical characteristics achieved an AUROC of 0.919 (95% CI 0.774-1.000; grade: weak). The corresponding sensitivity was 0.54‐0.77, and the specificity was 0.88‐0.98. Given the paucity of available studies using CT-based radiomics combined with clinical feature models, subgroup analyses could not be performed (Tables4  5).

### Other Risk Stratification Outcomes

HER2 is encoded by the ERBB2 gene. It plays a critical role in the development of various malignancies, including breast, gastric, bladder, ovarian, and lung cancers. HER2-targeted therapies have become first-line treatments for patients with advanced cancers exhibiting HER2 overexpression. Three studies reported on radiomics-based approaches to assess HER2 expression in bladder cancer. Yu et al [55] used MRI radiomics-based ML for noninvasive assessment of HER2. In their test set, an SVM model demonstrated an AUC of 0.712 (95% CI 0.535-0.889), with a sensitivity of 0.857 and a specificity of 0.533. They highlighted the potential value of this approach in cases where patients cannot undergo invasive procedures such as biopsies or diagnostic TUR. Based on contrast-enhanced computed tomography (CE-CT), Peng et al [74] assessed a clinical-radiomics model in evaluating HER2 status in urothelial bladder cancer. This CE-CT-based model exhibited the highest effectiveness in forecasting HER2 status, with an AUC of 0.814 (95% CI 0.642-0.986) in the test set. Wei et al [77] conducted a multicenter study using explainable ML based on CT radiomics to preoperatively diagnose HER2 status in bladder cancer. In their test set, AUCs were 0.803, 0.709, 0.679, 0.794, and 0.815 for LR, SVM, KNN, XGBoost, and RF models, respectively. These studies suggest that radiomics-based ML has promising potential for detecting HER2 expression in bladder cancer.

Ki-67 expression is associated with a poor prognosis and advanced clinicopathological features in cancer. Two single-center studies [35,76] explored radiomics-based prediction of Ki-67 expression in bladder cancer, with the potential to improve prognostic assessment and clinical decision-making. Zheng et al [35] reported on an MRI-based radiomics study. Their SMOTE-LASSO model achieved an AUC of 0.819 (0.658-0.980) in the validation set, with a sensitivity of 0.795 and a specificity of 0.867%. Feng et al [76] developed a radiomics nomogram based on CE-CT, which demonstrated AUCs of 0.836 and 0.887 in their validation set. These findings indicate that radiomics-based ML holds promise for detecting Ki-67 expression in bladder cancer.

Additionally, 2 [37,68] studies reported on the use of radiomics to predict LN staging. Gresser et al [68] used CT radiomics to evaluate LN staging in bladder cancer. Their combined model incorporated manually segmented radiomic features and radiologist assessment. The model achieved an AUC of 0.81 (0.71-0.92), a sensitivity of 0.73 (0.55-0.88), and a specificity of 0.84 (0.76-0.92), respectively, in the test set. However, Starmans et al [37] conducted a multicenter study using preoperative CT radiomics. They found that in patients with cT2-T4aN0-N1M0 muscle-invasive bladder cancer (MIBC), radiomics was not helpful in differentiating pN+ and pN0 disease. This suggests that further research is needed to explore the effectiveness of radiomics-based ML in detecting LN staging in bladder cancer.

### Small-Study Effect Analysis

Funnel plots were generated to evaluate small-study effects in radiomics-based ML models for detecting muscle invasion and high-grade tumors. Significant small-study effects were observed in both the training and validation sets for assessing muscle invasion (*P*<.05; Figures S15-S16 in Multimedia Appendix 3), as well as for identifying high-grade tumors (*P*<.05; Figures S17–S18 in Multimedia Appendix 3).

### Meta-Regression Analysis

For the tasks of detecting muscle invasion, meta-regression was performed. Independent variables included case number, model type (LR [Reference] vs other ML), and modeling variables (Radiomics [Reference] vs. Radiomics +clinical). Separate analyses were conducted for models developed on CT or MRI in the training set and validated on CT or MRI in the validation set. The results indicated a significant association between case number and the AUC only in the validation set of MRI-based models for detecting muscle invasion (*P*<.05). No other variables demonstrated a significant influence (*P*>.05; Table S2 in Multimedia Appendix 4 and Table S3 in Multimedia Appendix 5). Meta-regression was not conducted for pathological grade due to an insufficient number of studies.

## Discussion

### Summary of the Main Findings

This systematic review incorporated 57 studies [28-84] to evaluate radiomics-based ML for preoperative risk stratification in bladder cancer. The findings indicated a high AUROC for detecting both muscle invasion and high-grade tumors. Additionally, radiomics showed potential for identifying HER2 and Ki-67 expression. However, evaluation via the RQS revealed an overall low methodological quality among the eligible studies. PROBAST-AI assessment revealed that the primary source of bias was in the model evaluation phase, primarily due to small validation set sizes or an absence of external validation. Consequently, the findings of this review should be interpreted with caution. Based on the present findings, radiomics is considered to have application potential. Nevertheless, the current evidence faces significant challenges, including methodological shortcomings and a high ROB, which currently preclude its readiness for clinical translation.

### Comparison With Previous Reviews

Previous studies have examined the application of radiomics in bladder cancer. Kozikowski et al [10] reviewed the prediction ability of radiomics in muscle-invasive cancer, ultimately including eight articles. The pooled estimated sensitivity and specificity were 82% (95% CI 77%‐86%) and 81% (95% CI 76%‐85%). However, the study did not classify or analyze CT and MRI image sources separately nor attempt to combine different ML approaches with clinical radiomics models. Boca et al [14] reviewed MRI-based radiomics studies in bladder cancer, ultimately including 26 articles with 2991 participants. Radiomics in these studies was primarily used for preoperative prediction of tumor stage or molecular correlations (n=9), preoperative tumor grading (n=13), and prediction of prognosis or response to neoadjuvant therapy (n=4). Most radiomics models incorporated second-order features from filtered images, with quality scores ranging from 8.33% to 52.77% [14]. However, the study only discussed MRI-based radiomics and failed to consider other imaging modalities. The study also did not analyze sensitivity, specificity, or dataset differences, nor did it incorporate a discussion of radiomics combined with clinical features. Building on these prior efforts, the current study conducted a more systematic and comprehensive review of the current state of radiomics-based ML in bladder cancer. The current study also considered different image features, accounted for various image sources, analyzed different modeling approaches, and examined the detection performance across diverse datasets. This provides more comprehensive evidence for future developments.

In the clinical management of bladder cancer, CT often struggles to accurately diagnose flat lesions and prostate-adjacent bladder base lesions, especially in patients with benign prostatic hyperplasia. This difficulty arises from the challenge of distinguishing tumor recurrence from inflammatory wall thickening following intravesical chemotherapy, as well as from scar tissue after TURBT [85]. MRI assessment is a laborious, slice-by-slice process, with its effectiveness depending on the radiologist’s experience [86]. The integration of radiomics and ML with bladder MRI holds promise for improving staging and treatment response assessment. Bladder cancer management guidelines would be enhanced by the integration of MRI into their staging strategies [87]. However, radiomics may experience similar slow progress as molecular biology-based diagnostic and therapeutic techniques. This can be attributed to several factors, such as technical complexities, a lack of validation standards, and inadequate study design (particularly conflating hypothesis generation with hypothesis testing) [11]. Other contributing factors include data overfitting, incomplete reporting of results, and unidentified confounding variables in the datasets used (especially in retrospective datasets). Therefore, as with all biomarker studies, retrospective radiomics studies require validation on independent datasets, ideally from another institution [11].

In clinical practice, MRI has become the most accurate imaging modality for evaluating local invasiveness in bladder cancer. It can be used to assess regional LN involvement and tumor spread to pelvic bones and the upper urinary tract [21,88]. While bladder cancer appears as a soft tissue lesion on CT scans, it is more easily identified as a filling defect during CT urography. Ultrasound is a dynamic imaging modality. It can distinguish bladder cancer from other conditions that appear similar. Its ability to detect blood flow aids in distinguishing the solid tissue of the tumor from blood clots or debris [89]. This meta-analysis reveals that in studies examining muscle invasion, the primary image sources are CT, MRI, and, to a lesser extent, ultrasound. Radiomics combined with clinical features has not consistently demonstrated superior detection performance, potentially due to limited available data. The diagnostic effectiveness of radiomics warrants further investigation. In studies investigating histological grading, the primary image sources are CT and MRI. Only a few studies have explored combining radiomics with clinical features, resulting in limited current evidence.

Traditional ML-based radiomics requires substantial upfront effort, including texture extraction, manual image segmentation, and model construction. This process carries the risk of image data loss and imposes a significant workload. Manual segmentation, in particular, can be influenced by clinical experience, habits, and prior research [86,90]. Consequently, some researchers have explored DL, leveraging its capabilities for staging, grading, automated tumor detection, intelligent segmentation, bladder wall segmentation, and prediction of recurrence, response to chemotherapy, and overall survival. The goal is to improve disease management [58,91-94]. While external validation of intelligent segmentation accuracy is lacking, the potential of DL to reduce workload and automatically interpret images in an intelligent manner is encouraging the development of better, smarter tools. Of the studies included in this review, only a few DL models have demonstrated good performance. Thus, future research should explore DL in addition to traditional methods.

While the models in this study demonstrated relatively high AUROC values, the widespread absence of calibration metrics represents a major limitation. This gap substantially undermines the reliability of these models for practical application [95,96]. Dependence on discriminatory metrics such as AUC, sensitivity, and specificity only reflects the ability of a model to differentiate between positive and negative events. These metrics do not, however, inform on the accuracy of the predicted probabilities—a critical shortfall that poses challenges for safe integration into clinical decision support [97,98]. Consequently, future studies must report comprehensive calibration metrics, including calibration curves, calibration slope, intercept, and the Brier score. Furthermore, decision curve analysis should be performed to evaluate the clinical net benefit across different decision thresholds, a crucial step for assessing clinical utility.

Before initiating this meta-analysis, a prospective registration was completed. However, several adjustments were made during the actual research process. First, to mitigate the risk of missing newly published literature and ensure the completeness of evidence, supplementary database searches were conducted on October 17, 2025. Second, to better reflect the ROB in the included studies, PROBAST-AI was adopted in addition to RQS for the assessment of bias. Third, the GRADE approach was applied to evaluate the certainty of evidence. Fourth, subgroup analyses were carried out based on the type of dataset (training vs validation), imaging source, and type of model. Finally, analyses of small-study effects and meta-regression were also performed. The registered protocol will be updated accordingly.

### Strengths and Limitations

This meta-analysis is the first comprehensive and systematic review of preoperative risk stratification for bladder cancer, summarizing the available evidence. However, it is subject to the following limitations. First, the present analysis incorporated only 16 studies [29-32,42,47,50,52,58-60,77,78,80,81,83] that used external validation. Among these, 13 investigations focused on muscle invasion, one examined histological grade, one assessed HER2 expression, and one addressed preoperative LN staging. The analytical strategy required subgrouping based on the imaging modality used for model development and the integration of clinical features. Incorporating the data source as a subgroup variable was precluded due to the limited number of multicenter datasets, which would have yielded statistically unreliable comparisons. Furthermore, the validation sets in this study were predominantly created via random sampling. The lack of independent external validation limits the interpretation of the results to some extent. Second, different mathematical models exhibit varying performance in processing images. Although subgroup analyses were conducted to account for these differences, the small number of models within each subgroup may limit the interpretation of our findings. Therefore, the comparative performance of models within these subgroups should be interpreted with caution. Third, evidence for the preoperative identification of LN metastasis and Ki-67 expression is extremely limited. Fourth, while subgroup analyses were performed based on different model types, definitive conclusions cannot be drawn. However, given the paucity of available studies and the small number of articles included in specific model categories, definitive conclusions cannot be drawn. Fifth, the RQS and PROBAST-AI instruments incorporate elements requiring subjective judgment, which may introduce variability into the overall interpretation of the quality assessments. Sixth, a notable limitation of the current evidence base is the infrequent reporting of calibration metrics, such as the calibration-in-the-large, calibration slope, and Brier score. This omission considerably diminishes the clinical applicability of the model performance estimates. Seventh, while all eligible studies confirmed diagnoses pathologically, the transparency regarding the origin of pathological specimens was often insufficient. This lack of detail concerning specimen sourcing is a potential source of bias. Together, these limitations collectively curtail the generalizability of the study findings. Eighth, a notable small-study effect was observed across the included studies. Thus, the findings should be interpreted with caution.

### Challenges and Future Directions

In summary, significant challenges remain prior to the clinical deployment of these models. First, current investigations have not rigorously examined the impact of imaging modality. Variations in acquisition protocols can influence image quality; however, the potential effect of such heterogeneity on modeling outcomes has not been adequately addressed in the existing literature. Second, independent external validation is essential for establishing the generalizability of models. The predominant reliance on internal validation in the eligible studies implies that these findings propose a potential methodological approach rather than one ready for clinical application. Substantial additional evidence is required to confirm clinical utility and generalizability. Third, sample size presents a major constraint. Only a few studies provided a sufficient number of cases, and most encompassed fewer than 200 subjects. Consequently, discussions regarding model robustness necessitate larger, more powerful datasets. Fourth, the image segmentation process depends heavily on manual delineation. This approach is time-consuming and susceptible to variability introduced by the operator’s prior knowledge. Future work should prioritize developing and implementing DL-based automated or semiautomated segmentation techniques.

To facilitate successful clinical translation, future investigations should prioritize the following areas: conducting robust pilot studies, performing reproducibility analyses on different imaging protocols and segmentation methods, ensuring adequate sample sizes, implementing multicenter external validation, comparing diverse ML architectures, and establishing protocols for continuous model updating and refinement.

### Conclusions

This is the first systematic review to comprehensively assess radiomics for preoperative risk stratification in bladder cancer. The findings provide evidence to support the development and refinement of future ML-based tools for image analysis. However, due to limitations in the current evidence, such as methodological flaws and a high ROB, low GRADE level, clinical translation is not yet warranted. Future research should standardize radiomics workflows, incorporate multicenter images from diverse geographical regions, and minimize the impact of imaging protocols and pre-processing steps. These measures are essential to advance radiomics toward successful clinical implementation. Such efforts are essential for fully elucidating and validating the potential of radiomics in the noninvasive diagnosis of bladder cancer.

## Supplementary material

10.2196/81084 Multimedia Appendix 1 Literature search strategy.

10.2196/81084 Multimedia Appendix 2 Quality assessment of included studies using the radiomics quality score (RQS) tool.

10.2196/81084 Multimedia Appendix 3 Forest and funnel plots for preoperative risk stratification using radiomics-based machine learning.

10.2196/81084 Multimedia Appendix 4 Meta-regression of area under the curve (AUC) for machine learning models based on computed tomography (CT) and magnetic resonance imaging (MRI) radiomics in detecting muscle invasion (training set).

10.2196/81084 Multimedia Appendix 5 Meta-regression of area under the curve (AUC) for machine learning models based on computed tomography (CT) and magnetic resonance imaging (MRI) radiomics in detecting muscle invasion (validation set).

10.2196/81084 Checklist 1 PRISMA 2020 checklist.

## Abbreviations

AI: artificial intelligence
AUC: area under the curve
AUROC: area under the receiver operating characteristic curve
CE-CT: contrast-enhanced computed tomography
CT: computed tomography
DL: deep learning
GRADE: Grading of Recommendations, Assessment, Development, and Evaluation
HER2: human epidermal growth factor receptor 2
LN: lymph node
LR: logistic regression
MIBC: muscle-invasive bladder cancer
ML: machine learning
MRI: magnetic resonance imaging
PRISMA: Preferred Reporting Items for Systematic Reviews and Meta-Analyses
PROBAST-AI: Prediction Model Risk of Bias Assessment Tool for artificial intelligence
PROSPERO: International Prospective Register of Systematic Reviews
ROB: risk of bias
ROI: region of interest
RQS: Radiomics Quality Scoring
SVM: support vector machine
TURBT: Transurethral resection of bladder tumor
