# Supplementary material for: Application Value of Radiomics-Based Machine Learning for Preoperative Risk Stratification of Bladder Cancer: Systematic Review and Meta-Analysis
Source: J Med Internet Res. 2026 Jun 12;28:e81084. doi: 10.2196/81084 (PMC13263024; doi:10.2196/81084)
Supplement: Multimedia Appendix 1 [file jmir-v28-e81084-s001.docx]

# Supplementary Material 1 Literature search strategy

**1.Pubmed**

| Search number | Query |
| --- | --- |
| #1 | "Urinary Bladder Neoplasms"[Mesh] |
| #2 | (((((((((((((((((((Urinary Bladder Neoplasms[Title/Abstract]) OR (Bladder Neoplasms[Title/Abstract])) OR (Bladder Neoplasm[Title/Abstract])) OR (Bladder Tumors[Title/Abstract])) OR (Bladder Tumor[Title/Abstract])) OR (Bladder Cancer[Title/Abstract])) OR (Bladder Cancers[Title/Abstract])) OR (Cancer of Bladder[Title/Abstract])) OR (Cancer of the Bladder[Title/Abstract])) OR (Tumor of Urinary Bladder[Title/Abstract])) OR (bladder malignancies[Title/Abstract])) OR (bladder malignancy[Title/Abstract])) OR (carcinomatous bladder[Title/Abstract])) OR (bladder tumours[Title/Abstract])) OR (bladder tumour[Title/Abstract])) OR (neoplasm of the bladder[Title/Abstract])) OR (neoplasms of the bladder[Title/Abstract])) OR (tumor of the bladder[Title/Abstract])) OR (tumors of the bladder[Title/Abstract])) OR (bladder carcinoma[Title/Abstract]) |
| #3 | ("Urinary Bladder Neoplasms"[Mesh]) OR ((((((((((((((((((((Urinary Bladder Neoplasms[Title/Abstract]) OR (Bladder Neoplasms[Title/Abstract])) OR (Bladder Neoplasm[Title/Abstract])) OR (Bladder Tumors[Title/Abstract])) OR (Bladder Tumor[Title/Abstract])) OR (Bladder Cancer[Title/Abstract])) OR (Bladder Cancers[Title/Abstract])) OR (Cancer of Bladder[Title/Abstract])) OR (Cancer of the Bladder[Title/Abstract])) OR (Tumor of Urinary Bladder[Title/Abstract])) OR (bladder malignancies[Title/Abstract])) OR (bladder malignancy[Title/Abstract])) OR (carcinomatous bladder[Title/Abstract])) OR (bladder tumours[Title/Abstract])) OR (bladder tumour[Title/Abstract])) OR (neoplasm of the bladder[Title/Abstract])) OR (neoplasms of the bladder[Title/Abstract])) OR (tumor of the bladder[Title/Abstract])) OR (tumors of the bladder[Title/Abstract])) OR (bladder carcinoma[Title/Abstract])) |
| #4 | machine learning[MeSH Terms] |
| #5 | (((((((((((((((((((((((((((((machine learning[Title/Abstract]) OR (Transfer Learning[Title/Abstract])) OR (Deep learning[Title/Abstract])) OR (Ensemble Learning[Title/Abstract])) OR (artificial intelligence[Title/Abstract])) OR (random forest[Title/Abstract])) OR (neural network[Title/Abstract])) OR (neural networks[Title/Abstract])) OR (K-Nearest Neighbor[Title/Abstract])) OR (CNN[Title/Abstract])) OR (Support vector machine[Title/Abstract])) OR (SVM[Title/Abstract])) OR (Gradient Boosting Machine[Title/Abstract])) OR (Nomogram[Title/Abstract])) OR (XGBoost[Title/Abstract])) OR (Adaboost[Title/Abstract])) OR (Decision tree[Title/Abstract])) OR (ResNet-50[Title/Abstract])) OR (ResNet[Title/Abstract])) OR (AlexNet[Title/Abstract])) OR (VGGNet[Title/Abstract])) OR (GoogLeNet[Title/Abstract])) OR (Naive Bayesian[Title/Abstract])) OR (Multilayer perceptron[Title/Abstract])) OR (Bayesian network[Title/Abstract])) OR (Radiomics[Title/Abstract])) OR (Radiomic[Title/Abstract])) OR (radiomics-based[Title/Abstract])) OR (radiomic signature[Title/Abstract])) OR (Texture[Title/Abstract]) |
| #6 | (machine learning[MeSH Terms]) OR ((((((((((((((((((((((((((((((machine learning[Title/Abstract]) OR (Transfer Learning[Title/Abstract])) OR (Deep learning[Title/Abstract])) OR (Ensemble Learning[Title/Abstract])) OR (artificial intelligence[Title/Abstract])) OR (random forest[Title/Abstract])) OR (neural network[Title/Abstract])) OR (neural networks[Title/Abstract])) OR (K-Nearest Neighbor[Title/Abstract])) OR (CNN[Title/Abstract])) OR (Support vector machine[Title/Abstract])) OR (SVM[Title/Abstract])) OR (Gradient Boosting Machine[Title/Abstract])) OR (Nomogram[Title/Abstract])) OR (XGBoost[Title/Abstract])) OR (Adaboost[Title/Abstract])) OR (Decision tree[Title/Abstract])) OR (ResNet-50[Title/Abstract])) OR (ResNet[Title/Abstract])) OR (AlexNet[Title/Abstract])) OR (VGGNet[Title/Abstract])) OR (GoogLeNet[Title/Abstract])) OR (Naive Bayesian[Title/Abstract])) OR (Multilayer perceptron[Title/Abstract])) OR (Bayesian network[Title/Abstract])) OR (Radiomics[Title/Abstract])) OR (Radiomic[Title/Abstract])) OR (radiomics-based[Title/Abstract])) OR (radiomic signature[Title/Abstract])) OR (Texture[Title/Abstract])) |
| #7 | (("Urinary Bladder Neoplasms"[Mesh]) OR ((((((((((((((((((((Urinary Bladder Neoplasms[Title/Abstract]) OR (Bladder Neoplasms[Title/Abstract])) OR (Bladder Neoplasm[Title/Abstract])) OR (Bladder Tumors[Title/Abstract])) OR (Bladder Tumor[Title/Abstract])) OR (Bladder Cancer[Title/Abstract])) OR (Bladder Cancers[Title/Abstract])) OR (Cancer of Bladder[Title/Abstract])) OR (Cancer of the Bladder[Title/Abstract])) OR (Tumor of Urinary Bladder[Title/Abstract])) OR (bladder malignancies[Title/Abstract])) OR (bladder malignancy[Title/Abstract])) OR (carcinomatous bladder[Title/Abstract])) OR (bladder tumours[Title/Abstract])) OR (bladder tumour[Title/Abstract])) OR (neoplasm of the bladder[Title/Abstract])) OR (neoplasms of the bladder[Title/Abstract])) OR (tumor of the bladder[Title/Abstract])) OR (tumors of the bladder[Title/Abstract])) OR (bladder carcinoma[Title/Abstract]))) AND ((machine learning[MeSH Terms]) OR ((((((((((((((((((((((((((((((machine learning[Title/Abstract]) OR (Transfer Learning[Title/Abstract])) OR (Deep learning[Title/Abstract])) OR (Ensemble Learning[Title/Abstract])) OR (artificial intelligence[Title/Abstract])) OR (random forest[Title/Abstract])) OR (neural network[Title/Abstract])) OR (neural networks[Title/Abstract])) OR (K-Nearest Neighbor[Title/Abstract])) OR (CNN[Title/Abstract])) OR (Support vector machine[Title/Abstract])) OR (SVM[Title/Abstract])) OR (Gradient Boosting Machine[Title/Abstract])) OR (Nomogram[Title/Abstract])) OR (XGBoost[Title/Abstract])) OR (Adaboost[Title/Abstract])) OR (Decision tree[Title/Abstract])) OR (ResNet-50[Title/Abstract])) OR (ResNet[Title/Abstract])) OR (AlexNet[Title/Abstract])) OR (VGGNet[Title/Abstract])) OR (GoogLeNet[Title/Abstract])) OR (Naive Bayesian[Title/Abstract])) OR (Multilayer perceptron[Title/Abstract])) OR (Bayesian network[Title/Abstract])) OR (Radiomics[Title/Abstract])) OR (Radiomic[Title/Abstract])) OR (radiomics-based[Title/Abstract])) OR (radiomic signature[Title/Abstract])) OR (Texture[Title/Abstract]))) |

**2.Cochrane**

| Search number | Query |
| --- | --- |
| #1 | MeSH descriptor: [Urinary Bladder Neoplasms] explode all trees |
| #2 | (Urinary Bladder Neoplasm):ti,ab,kw OR (Bladder Neoplasms):ti,ab,kw OR (Bladder Neoplasm):ti,ab,kw OR (Bladder Tumors):ti,ab,kw OR (Bladder Tumor):ti,ab,kw |
| #3 | (Bladder Cancer):ti,ab,kw OR (Bladder Cancers):ti,ab,kw OR (Cancer of Bladder):ti,ab,kw OR (Cancer of the Bladder):ti,ab,kw OR (Tumor of Urinary Bladder):ti,ab,kw |
| #4 | (bladder malignancies):ti,ab,kw OR (bladder malignancy):ti,ab,kw OR (carcinomatous bladder):ti,ab,kw OR (bladder tumours):ti,ab,kw OR (bladder tumour):ti,ab,kw |
| #5 | (neoplasm of the bladder):ti,ab,kw OR (neoplasms of the bladder):ti,ab,kw OR (tumor of the bladder):ti,ab,kw OR (tumors of the bladder):ti,ab,kw OR (bladder carcinoma):ti,ab,kw |
| #6 | #1 or #2 or #3 or #4 or #5 |
| #7 | MeSH descriptor: [Machine Learning] explode all trees |
| #8 | (machine learning):ti,ab,kw OR (Transfer Learning):ti,ab,kw OR (Deep learning):ti,ab,kw OR (Ensemble Learning):ti,ab,kw OR (artificial intelligence):ti,ab,kw |
| #9 | (random forest):ti,ab,kw OR (neural network):ti,ab,kw OR (neural networks):ti,ab,kw OR (K-Nearest Neighbor):ti,ab,kw OR (CNN):ti,ab,kw |
| #10 | (Support vector machine):ti,ab,kw OR (SVM):ti,ab,kw OR (Gradient Boosting Machine):ti,ab,kw OR (Nomogram):ti,ab,kw OR (XGBoost):ti,ab,kw |
| #11 | (Adaboost):ti,ab,kw OR (Decision tree):ti,ab,kw OR (ResNet-50):ti,ab,kw OR (ResNet):ti,ab,kw OR (AlexNet):ti,ab,kw |
| #12 | (VGGNet):ti,ab,kw OR (GoogLeNet):ti,ab,kw OR (Naive Bayesian):ti,ab,kw OR (Multilayer perceptron):ti,ab,kw OR (Bayesian network):ti,ab,kw |
| #13 | (Radiomics):ti,ab,kw OR (Radiomic):ti,ab,kw OR (radiomics-based):ti,ab,kw OR (radiomic signature):ti,ab,kw OR (Texture):ti,ab,kw |
| #14 | #7 or #8 or #9 or #10 or #11 or #12 or #13 |
| #15 | #6 and #14 |

**3.Embase**

| Search number | Query |
| --- | --- |
| #1 | 'bladder tumor'/exp |
| #2 | 'urinary bladder neoplasms':ab,ti OR 'bladder neoplasms':ab,ti OR 'bladder neoplasm':ab,ti OR 'bladder tumors':ab,ti OR 'bladder tumor':ab,ti OR 'bladder cancer':ab,ti OR 'bladder cancers':ab,ti OR 'cancer of bladder':ab,ti OR 'cancer of the bladder':ab,ti OR 'tumor of urinary bladder':ab,ti OR 'bladder malignancies':ab,ti OR 'bladder malignancy':ab,ti OR 'carcinomatous bladder':ab,ti OR 'bladder tumours':ab,ti OR 'bladder tumour':ab,ti OR 'neoplasm of the bladder':ab,ti OR 'neoplasms of the bladder':ab,ti OR 'tumor of the bladder':ab,ti OR 'tumors of the bladder':ab,ti OR 'bladder carcinoma':ab,ti |
| #3 | #1 OR #2 |
| #4 | 'machine learning'/exp |
| #5 | 'machine learning':ab,ti OR 'transfer learning':ab,ti OR 'deep learning':ab,ti OR 'ensemble learning':ab,ti OR 'artificial intelligence':ab,ti OR 'random forest':ab,ti OR 'neural network':ab,ti OR 'neural networks':ab,ti OR 'k-nearest neighbor':ab,ti OR cnn:ab,ti OR 'support vector machine':ab,ti OR svm:ab,ti OR 'gradient boosting machine':ab,ti OR nomogram:ab,ti OR xgboost:ab,ti OR adaboost:ab,ti OR 'decision tree':ab,ti OR 'resnet 50':ab,ti OR resnet:ab,ti OR alexnet:ab,ti OR vggnet:ab,ti OR googlenet:ab,ti OR 'naive bayesian':ab,ti OR 'multilayer perceptron':ab,ti OR 'bayesian network':ab,ti OR radiomics:ab,ti OR radiomic:ab,ti OR 'radiomics based':ab,ti OR 'radiomic signature':ab,ti OR texture:ab,ti |
| #6 | #4 OR #5 |
| #7 | #3 AND #6 |

**4.Web of science**

| Search number | Query |
| --- | --- |
| #1 | Urinary Bladder Neoplasms (Topic) OR Bladder Neoplasms (Topic) OR Bladder Neoplasm (Topic) OR Bladder Tumors (Topic) OR Bladder Tumor (Topic) OR Bladder Cancer (Topic) OR Bladder Cancers (Topic) OR Cancer of Bladder (Topic) OR Cancer of the Bladder (Topic) OR Tumor of Urinary Bladder (Topic) OR bladder malignancies (Topic) OR bladder malignancy (Topic) OR carcinomatous bladder (Topic) OR bladder tumours (Topic) OR bladder tumour (Topic) OR neoplasm of the bladder (Topic) OR neoplasms of the bladder (Topic) OR tumor of the bladder (Topic) OR tumors of the bladder (Topic) OR bladder carcinoma (Topic) |
| #2 | machine learning (Topic) OR Transfer Learning (Topic) OR Deep learning (Topic) OR Ensemble Learning (Topic) OR artificial intelligence (Topic) OR random forest (Topic) OR neural network (Topic) OR neural networks (Topic) OR K-Nearest Neighbor (Topic) OR CNN (Topic) OR Support vector machine (Topic) OR SVM (Topic) OR Gradient Boosting Machine (Topic) OR Nomogram (Topic) OR XGBoost (Topic) OR Adaboost (Topic) OR Decision tree (Topic) OR ResNet-50 (Topic) OR ResNet (Topic) OR AlexNet (Topic) OR VGGNet (Topic) OR GoogLeNet (Topic) OR Naive Bayesian (Topic) OR Multilayer perceptron (Topic) OR Bayesian network (Topic) OR Radiomics (Topic) OR Radiomic (Topic) OR radiomics-based (Topic) OR radiomic signature (Topic) OR Texture (Topic) |
| #3 | #1 AND #2 |
