# Supplementary material for: Application Value of Radiomics-Based Machine Learning for Preoperative Risk Stratification of Bladder Cancer: Systematic Review and Meta-Analysis
Source: J Med Internet Res. 2026 Jun 12;28:e81084. doi: 10.2196/81084 (PMC13263024; doi:10.2196/81084)
Supplement: Multimedia Appendix 2 [file jmir-v28-e81084-s002.docx]

**Table S1** Quality evaluation

| No | **Author** | **Year** | **v1** | **v2** | **v3** | **v4** | **v5** | **v6** | **v7** | **v8** | **v9** | **v10** | **v11** | **v12** | **v13** | **v14** | **v15** | **v16** | **Total** | **Proportion** |
| --- | --- | --- | --- | --- | --- | --- | --- | --- | --- | --- | --- | --- | --- | --- | --- | --- | --- | --- | --- | --- |
| 1 | Rui Zhang [28] | 2024 | 1 | 1 | 0 | 0 | 3 | 1 | 0 | 1 | 2 | 2 | 0 | 2 | 0 | 2 | 0 | 0 | 15 | 41.67 |
| 2 | Gumuyang Zhang [29] | 2021 | 1 | 1 | 0 | 0 | 3 | 0 | 0 | 0 | 1 | 0 | 0 | 3 | 0 | 1 | 0 | 2 | 12 | 33.33 |
| 3 | Yaojiang Ye [30] | 2023 | 1 | 0 | 0 | 0 | 3 | 0 | 0 | 0 | 1 | 0 | 0 | 3 | 0 | 0 | 0 | 1 | 9 | 25.00 |
| 4 | Jingyi Ren [31] | 2023 | 1 | 0 | 0 | 0 | 3 | 0 | 0 | 1 | 2 | 0 | 0 | 3 | 0 | 2 | 0 | 2 | 14 | 38.89 |
| 5 | Touseef Ahmad Qureshi [32] | 2024 | 0 | 0 | 0 | 0 | -3 | 1 | 0 | 0 | 0 | 0 | 0 | 3 | 0 | 0 | 0 | 0 | 1 | 2.78 |
| 6 | Harun Özdemir [33] | 2023 | 1 | 0 | 0 | 0 | 3 | 0 | 0 | 0 | 2 | 0 | 0 | -5 | 0 | 0 | 0 | 2 | 3 | 8.33 |
| 7 | Weitian Chen [34] | 2022 | 1 | 1 | 0 | 0 | 3 | 0 | 0 | 0 | 1 | 0 | 0 | 2 | 0 | 2 | 0 | 2 | 12 | 33.33 |
| 8 | Zongtai Zheng [35] | 2021 | 1 | 1 | 0 | 0 | 3 | 0 | 0 | 1 | 2 | 0 | 0 | 2 | 0 | 2 | 0 | 0 | 12 | 33.33 |
| 9 | Lei Ye [36] | 2023 | 1 | 1 | 0 | 0 | 3 | 0 | 0 | 0 | 2 | 0 | 0 | 3 | 0 | 0 | 0 | 3 | 13 | 36.11 |
| 10 | Martijn P. A. Starmans [37] | 2022 | 1 | 0 | 0 | 0 | -3 | 0 | 0 | 0 | 2 | 0 | 7 | 2 | 0 | 0 | 0 | 3 | 12 | 33.33 |
| 11 | Suryadipto Sarkar [38] | 2023 | 0 | 1 | 0 | 0 | 3 | 0 | 0 | 0 | 1 | 0 | 0 | -5 | 0 | 0 | 0 | 2 | 2 | 5.56 |
| 12 | Longchao Li [39] | 2023 | 1 | 1 | 0 | 0 | 3 | 1 | 0 | 1 | 2 | 2 | 0 | 2 | 0 | 0 | 1 | 3 | 17 | 47.22 |
| 13 | Zhikang Deng [40] | 2023 | 1 | 1 | 0 | 0 | 3 | 0 | 0 | 0 | 2 | 2 | 0 | 2 | 0 | 2 | 0 | 2 | 15 | 41.67 |
| 14 | Guihua Chen [41] | 2023 | 1 | 1 | 0 | 0 | 3 | 0 | 0 | 0 | 2 | 2 | 0 | 2 | 0 | 2 | 0 | 3 | 16 | 44.44 |
| 15 | Jie Yu [42] | 2024 | 1 | 1 | 0 | 0 | 3 | 0 | 0 | 0 | 1 | 0 | 0 | 3 | 0 | 0 | 0 | 3 | 12 | 33.33 |
| 16 | Shuaishuai Xu [43] | 2019 | 1 | 1 | 0 | 0 | 3 | 0 | 0 | 0 | 1 | 0 | 0 | 2 | 0 | 0 | 0 | 2 | 10 | 27.78 |
| 17 | Zhe Zhou [44] | 2019 | 1 | 1 | 0 | 0 | 3 | 0 | 0 | 0 | 1 | 0 | 0 | 2 | 0 | 0 | 0 | 2 | 10 | 27.78 |
| 18 | Yuhan Yang [45] | 2021 | 1 | 1 | 0 | 0 | 3 | 0 | 0 | 0 | 1 | 0 | 0 | 2 | 0 | 0 | 0 | 3 | 11 | 30.56 |
| 20 | Zongtai Zheng [46] | 2021 | 1 | 1 | 0 | 0 | 3 | 0 | 0 | 0 | 2 | 2 | 0 | 2 | 0 | 2 | 0 | 2 | 15 | 41.67 |
| 21 | Hongzheng Song [47] | 2023 | 1 | 1 | 0 | 0 | 3 | 0 | 0 | 0 | 2 | 2 | 0 | 3 | 0 | 2 | 0 | 2 | 16 | 44.44 |
| 22 | Y. Cui [48] | 2022 | 1 | 1 | 0 | 0 | 3 | 0 | 0 | 0 | 1 | 0 | 0 | 2 | 0 | 0 | 0 | 3 | 11 | 30.56 |
| 23 | Gumuyang Zhang [49] | 2020 | 1 | 1 | 0 | 0 | 3 | 0 | 0 | 0 | 1 | 0 | 0 | 2 | 0 | 0 | 0 | 2 | 10 | 27.78 |
| 24 | Zongjie Wei [50] | 2023 | 1 | 1 | 0 | 0 | 3 | 0 | 0 | 0 | 1 | 0 | 0 | 3 | 0 | 2 | 0 | 2 | 13 | 36.11 |
| 25 | Junjiong Zheng [51] | 2019 | 1 | 1 | 0 | 0 | 3 | 1 | 0 | 0 | 2 | 2 | 0 | 2 | 0 | 2 | 0 | 2 | 16 | 44.44 |
| 26 | Huanjun Wang [52] | 2019 | 1 | 1 | 0 | 0 | 3 | 1 | 0 | 1 | 1 | 0 | 0 | 3 | 0 | 2 | 0 | 3 | 16 | 44.44 |
| 27 | Zongtai Zheng [53] | 2021 | 1 | 1 | 0 | 0 | 3 | 1 | 0 | 0 | 1 | 0 | 0 | 2 | 0 | 2 | 0 | 3 | 14 | 38.89 |
| 28 | Wei Wang [54] | 2022 | 1 | 1 | 0 | 0 | 3 | 1 | 0 | 0 | 1 | 0 | 0 | 2 | 0 | 0 | 0 | 3 | 12 | 33.33 |
| 30 | Ruixi Yu [55] | 2023 | 1 | 1 | 0 | 0 | 3 | 0 | 0 | 0 | 1 | 0 | 0 | 2 | 0 | 0 | 0 | 3 | 11 | 30.56 |
| 31 | W. Wang [56] | 2023 | 1 | 1 | 0 | 0 | 3 | 0 | 0 | 0 | 2 | 0 | 0 | 2 | 0 | 0 | 0 | 2 | 11 | 30.56 |
| 32 | Lu Zhang [57] | 2022 | 1 | 1 | 0 | 0 | 3 | 1 | 0 | 0 | 2 | 0 | 0 | 2 | 0 | 0 | 0 | 3 | 13 | 36.11 |
| 34 | Yuan Zou [58] | 2022 | 1 | 1 | 0 | 0 | 3 | 0 | 0 | 0 | 1 | 0 | 0 | 4 | 0 | 0 | 0 | 3 | 13 | 36.11 |
| 35 | Xiaoqian Zhou [59] | 2022 | 1 | 1 | 0 | 0 | 3 | 1 | 0 | 0 | 1 | 0 | 0 | 3 | 0 | 0 | 0 | 2 | 12 | 33.33 |
| 36 | Jianpeng Li [60] | 2023 | 1 | 0 | 0 | 0 | 3 | 0 | 0 | 0 | 1 | 0 | 0 | 3 | 0 | 0 | 0 | 2 | 10 | 27.78 |
| 37 | Longchao Li [61] | 2024 | 1 | 1 | 0 | 0 | 3 | 1 | 0 | 0 | 1 | 0 | 0 | 2 | 0 | 0 | 0 | 1 | 10 | 27.78 |
| 38 | Li Chen [62] | 2022 | 1 | 1 | 0 | 0 | 3 | 0 | 0 | 1 | 1 | 0 | 0 | 2 | 0 | 0 | 0 | 1 | 10 | 27.78 |
| 39 | Xiaopan Xu [63] | 2017 | 1 | 1 | 0 | 0 | 3 | 0 | 0 | 0 | 1 | 0 | 0 | 2 | 0 | 0 | 0 | 3 | 11 | 30.56 |
| 40 | Xiaopan Xu [64] | 2018 | 1 | 1 | 0 | 0 | 3 | 0 | 0 | 0 | 1 | 0 | 0 | 2 | 0 | 0 | 0 | 3 | 11 | 30.56 |
| 41 | Rui-zhi Gao [65] | 2021 | 1 | 1 | 0 | 0 | 3 | 0 | 0 | 0 | 1 | 0 | 0 | 2 | 0 | 0 | 0 | 2 | 10 | 27.78 |
| 42 | Huanjun Wang [66] | 2019 | 1 | 1 | 0 | 0 | 3 | 1 | 0 | 0 | 1 | 0 | 0 | 2 | 0 | 0 | 0 | 2 | 11 | 30.56 |
| 43 | Xi Zhang [67] | 2017 | 1 | 1 | 0 | 0 | 3 | 0 | 0 | 0 | 1 | 0 | 0 | -5 | 0 | 0 | 0 | 2 | 3 | 8.33 |
| 44 | Eva Gresser [68] | 2022 | 1 | 1 | 0 | 0 | 3 | 1 | 0 | 0 | 1 | 0 | 0 | 2 | 0 | 0 | 0 | 2 | 11 | 30.56 |
| 45 | Yubing Tong [69] | 2018 | 1 | 1 | 0 | 0 | -3 | 0 | 0 | 0 | 0 | 0 | 0 | 2 | 0 | 0 | 0 | 2 | 3 | 8.33 |
| 46 | Tingting Tao [70] | 2024 | 1 | 1 | 0 | 0 | 3 | 0 | 0 | 0 | 2 | 0 | 0 | 2 | 0 | 0 | 0 | 3 | 12 | 33.33 |
| 47 | Yan Liu [71] | 2022 | 1 | 1 | 0 | 0 | 3 | 0 | 0 | 0 | 2 | 0 | 0 | 2 | 0 | 0 | 0 | 2 | 11 | 30.56 |
| 48 | Dongmei Liu [72] | 2022 | 1 | 1 | 0 | 0 | 3 | 0 | 0 | 0 | 2 | 0 | 0 | 2 | 0 | 0 | 0 | 2 | 11 | 30.56 |
| 49 | ABDUL RAZIK [73] | 2021 | 1 | 1 | 0 | 0 | 3 | 0 | 0 | 0 | 1 | 0 | 0 | -5 | 0 | 0 | 0 | 1 | 2 | 5.56 |
| 47 | Jiao Peng [74] | 2024 | 1 | 1 | 0 | 0 | 3 | 1 | 0 | 0 | 2 | 0 | 0 | 2 | 0 | 2 | 0 | 2 | 14 | 38.89 |
| 48 | Situ Xiong [75] | 2024 | 1 | 1 | 0 | 0 | 3 | 0 | 0 | 0 | 2 | 0 | 0 | 2 | 0 | 2 | 0 | 1 | 12 | 33.33 |
| 50 | Shengxing Feng [76] | 2024 | 1 | 1 | 0 | 0 | 3 | 1 | 0 | 0 | 2 | 0 | 0 | 2 | 0 | 2 | 0 | 2 | 14 | 38.89 |
| 51 | Zongjie Wei [77] | 2024 | 1 | 1 | 0 | 0 | 3 | 1 | 0 | 0 | 2 | 0 | 0 | 5 | 0 | 2 | 0 | 2 | 17 | 47.22 |
| 51 | Lingkai Cai [78] | 2025 | 1 | 1 | 0 | 0 | 3 | 0 | 0 | 0 | 1 | 0 | 0 | 4 | 0 | 0 | 0 | 3 | 13 | 36.11 |
| 52 | Changyu Du [79] | 2025 | 1 | 1 | 0 | 0 | 3 | 0 | 0 | 0 | 2 | 2 | 0 | 2 | 0 | 2 | 0 | 3 | 16 | 44.44 |
| 53 | Yiheng Du [80] | 2025 | 1 | 1 | 0 | 0 | 3 | 0 | 0 | 0 | 2 | 0 | 0 | 3 | 0 | 0 | 0 | 3 | 13 | 36.11 |
| 55 | Xucheng He [81] | 2025 | 1 | 0 | 0 | 0 | 3 | 0 | 0 | 0 | 2 | 2 | 0 | 3 | 0 | 2 | 0 | 3 | 16 | 44.44 |
| 56 | Mengting Hu [82] | 2025 | 1 | 1 | 0 | 0 | 3 | 0 | 0 | 0 | 2 | 0 | 0 | 2 | 0 | 0 | 0 | 2 | 11 | 30.56 |
| 59 | Jie Yu [83] | 2025 | 1 | 1 | 0 | 0 | 3 | 0 | 0 | 0 | 2 | 0 | 0 | 3 | 0 | 0 | 0 | 3 | 13 | 36.11 |
| 60 | Qi Zhou [84] | 2025 | 1 | 1 | 0 | 0 | 3 | 0 | 0 | 0 | 2 | 2 | 0 | 3 | 0 | 2 | 0 | 3 | 17 | 47.22 |

References：

28. Zhang R, Jia S, Zhai L, Wu F, Zhang S, Li F. Predicting preoperative muscle invasion status for bladder cancer using computed tomography-based radiomics nomogram. BMC Med Imaging. Apr 27, 2024;24(1):98. [doi: 10.1186/s12880-024-01276-7] [Medline: 38678222]

29. Zhang G, Wu Z, Xu L, et al. Deep learning on enhanced CT images can predict the muscular invasiveness of bladder cancer. Front Oncol. 2021;11:654685. [doi: 10.3389/fonc.2021.654685] [Medline: 34178641]

30. Ye Y, Luo Z, Qiu Z, et al. Radiomics prediction of muscle invasion in bladder cancer using semi-automatic lesion segmentation of MRI compared with manual segmentation. Bioengineering (Basel). Nov 25, 2023;10(12):1355. [doi: 10.3390/bioengineering10121355] [Medline: 38135946]

31. Ren J, Gu H, Zhang N, Chen W. Preoperative CT-based radiomics for diagnosing muscle invasion of bladder cancer. Egypt J Radiol Nucl Med. 2023;54(1):131. [doi: 10.1186/s43055-023-01044-7]

32. Qureshi TA, Chen X, Xie Y, et al. MRI/RNA-seq-based radiogenomics and artificial intelligence for more accurate staging of muscle-invasive bladder cancer. Int J Mol Sci. Dec 20, 2023;25(1):88. [doi: 10.3390/ijms25010088] [Medline: 38203254]

33. Özdemir H, Azamat S, Sam Özdemir M. Can only the shape feature in radiomics help machine learning show that bladder cancer has invaded muscles? Cureus. Sep 2023;15(9):e45488. [doi: 10.7759/cureus.45488] [Medline: 37859896]

34. Chen W, Gong M, Zhou D, et al. CT-based deep learning radiomics signature for the preoperative prediction of the muscle-invasive status of bladder cancer. Front Oncol. 2022;12:1019749. [doi: 10.3389/fonc.2022.1019749] [Medline: 36544709]

35. Zheng Z, Gu Z, Xu F, et al. Magnetic resonance imaging-based radiomics signature for preoperative prediction of Ki67 expression in bladder cancer. Cancer Imaging. Dec 4, 2021;21(1):65. [doi: 10.1186/s40644-021-00433-3] [Medline: 34863282]

36. Ye L, Wang Y, Xiang W, Yao J, Liu J, Song B. Radiomic analysis of quantitative T2 mapping and conventional MRI in predicting histologic grade of bladder cancer. J Clin Med. Sep 11, 2023;12(18):5900. [doi: 10.3390/jcm12185900] [Medline: 37762841]

37. Starmans MPA, Ho LS, Smits F, et al. Optimization of preoperative lymph node staging in patients with muscle-invasive bladder cancer using radiomics on computed tomography. J Pers Med. Apr 30, 2022;12(5):726. [doi: 10.3390/jpm12050726] [Medline: 35629148]

38. Sarkar S, Min K, Ikram W, et al. Performing automatic identification and staging of urothelial carcinoma in bladder cancer patients using a hybrid deep-machine learning approach. Cancers (Basel). Mar 8, 2023;15(6):1673. [doi: 10.3390/cancers15061673] [Medline: 36980557]

39. Li L, Zhang J, Zhe X, et al. An MRI-based radiomics nomogram in predicting histologic grade of non-muscle-invasive bladder cancer. Front Oncol. 2023;13:1025972. [doi: 10.3389/fonc.2023.1025972] [Medline: 37007156]

40. Deng Z, Dong W, Xiong S, et al. Machine learning models combining computed tomography semantic features and selected clinical variables for accurate prediction of the pathological grade of bladder cancer. Front Oncol. 2023;13:1166245. [doi: 10.3389/fonc.2023.1166245] [Medline: 37223680]

41. Chen G, Fan X, Wang T, et al. A machine learning model based on MRI for the preoperative prediction of bladder cancer invasion depth. Eur Radiol. Dec 2023;33(12):8821-8832. [doi: 10.1007/s00330-023-09960-y] [Medline: 37470826]

42. Yu J, Cai L, Chen C, et al. A novel predict method for muscular invasion of bladder cancer based on 3D mp-MRI feature fusion. Phys Med Biol. Feb 22, 2024;69(5). [doi: 10.1088/1361-6560/ad25c7] [Medline: 38306973]

43. Xu S, Yao Q, Liu G, et al. Combining DWI radiomics features with transurethral resection promotes the differentiation between muscle-invasive bladder cancer and non-muscle-invasive bladder cancer. Eur Radiol. Mar 2020;30(3):1804-1812. [doi: 10.1007/s00330-019-06484-2] [Medline: 31773297]

44. Zhou Z, Liu L, Xue K, Ma Y, Liu J, Zhang M. Assessment of pathological grading of bladder cancer using texture features from MRI. Presented at: 2019 IEEE International Conference on Mechatronics and Automation (ICMA); Aug 4-7, 2019:1333-1337; Tianjin, China. 2019.URL: <https://ieeexplore.ieee.org/xpl/mostRecentIssue.jsp?punumber=8801712> [doi: 10.1109/ICMA.2019.8816242]

45. Yang Y, Zou X, Wang Y, Ma X. Application of deep learning as a noninvasive tool to differentiate muscle-invasive bladder cancer and non-muscle-invasive bladder cancer with CT. Eur J Radiol. Jun 2021;139:109666. [doi: 10.1016/j.ejrad.2021.109666] [Medline: 33798819]

46. Zheng Z, Xu F, Gu Z, et al. Combining Multiparametric MRI Radiomics Signature With the Vesical Imaging-Reporting and Data System (VI-RADS) Score to Preoperatively Differentiate Muscle Invasion of Bladder Cancer. Front Oncol. 2021;11:619893. [doi: 10.3389/fonc.2021.619893] [Medline: 34055600]

47. Song H, Yang S, Yu B, et al. CT-based deep learning radiomics nomogram for the prediction of pathological grade in bladder cancer: a multicenter study. Cancer Imaging. Sep 18, 2023;23(1):89. [doi: 10.1186/s40644-023-00609-z] [Medline: 37723572]

48. Cui Y, Sun Z, Liu X, Zhang X, Wang X. CT-based radiomics for the preoperative prediction of the muscle-invasive status of bladder cancer and comparison to radiologists’ assessment. Clin Radiol. Jun 2022;77(6):e473-e482. [doi: 10.1016/j.crad.2022.02.019] [Medline: 35367051]

49. Zhang G, Xu L, Zhao L, et al. CT-based radiomics to predict the pathological grade of bladder cancer. Eur Radiol. Dec 2020;30(12):6749-6756. [doi: 10.1007/s00330-020-06893-8] [Medline: 32601949]

50. Wei Z, Liu H, Xv Y, et al. Development and validation of a CT-based deep learning radiomics nomogram to predict muscle invasion in bladder cancer. Heliyon. Jan 30, 2024;10(2):e24878. [doi: 10.1016/j.heliyon.2024.e24878] [Medline: 38304824]

51. Zheng J, Kong J, Wu S, et al. Development of a noninvasive tool to preoperatively evaluate the muscular invasiveness of bladder cancer using a radiomics approach. Cancer. Dec 15, 2019;125(24):4388-4398. [doi: 10.1002/cncr.32490] [Medline: 31469418]

52. Wang H, Xu X, Zhang X, et al. Elaboration of a multisequence MRI-based radiomics signature for the preoperative prediction of the muscle-invasive status of bladder cancer: a double-center study. Eur Radiol. Sep 2020;30(9):4816-4827. [doi: 10.1007/s00330-020-06796-8] [Medline: 32318846]

53. Zheng Z, Xu F, Gu Z, et al. Integrating multiparametric MRI radiomics features and the Vesical Imaging-Reporting and Data System (VI-RADS) for bladder cancer grading. Abdom Radiol (NY). Sep 2021;46(9):4311-4323. [doi: 10.1007/s00261-021-03108-6] [Medline: 33978825]

54. Wang W, Li W, Wang K, et al. Integrating radiomics with the vesical imaging-reporting and data system to predict muscle invasion of bladder cancer. Urol Oncol. Jun 2023;41(6):294. [doi: 10.1016/j.urolonc.2022.10.024] [Medline: 36526525]

55. Yu R, Cai L, Gong Y, et al. MRI-based machine learning radiomics for preoperative assessment of human epidermal growth factor receptor 2 status in urothelial bladder carcinoma. J Magn Reson Imaging. Dec 2024;60(6):2694-2704. [doi: 10.1002/jmri.29342] [Medline: 38456745]

56. Wang W, Wang K, Qiu J, et al. MRI-based radiomics analysis of bladder cancer: prediction of pathological grade and histological variant. Clin Radiol. Nov 2023;78(11):e889-e897. [doi: 10.1016/j.crad.2023.07.020] [Medline: 37633748]

57. Zhang L, Li X, Yang L, et al. Multi‐sequence and multi‐regional MRI ‐based radiomics nomogram for the preoperative assessment of muscle invasion in bladder cancer. J Magn Reson Imaging. Jul 2023;58(1):258-269. URL: <https://onlinelibrary.wiley.com/toc/15222586/58/1> [doi: 10.1002/jmri.28498] [Medline: 36300676]

58. Zou Y, Cai L, Chen C, et al. Multi-task deep learning based on T2-weighted images for predicting muscular-invasive bladder cancer. Comput Biol Med. Dec 2022;151(Pt A):106219. [doi: 10.1016/j.compbiomed.2022.106219] [Medline: 36343408]

59. Zhou X, Yue X, Xu Z, Denoeux T, Chen Y. PENet: Prior evidence deep neural network for bladder cancer staging. Methods. Nov 2022;207:20-28. [doi: 10.1016/j.ymeth.2022.08.010] [Medline: 36031139]

60. Li J, Qiu Z, Cao K, et al. Predicting muscle invasion in bladder cancer based on MRI: a comparison of radiomics, and single-task and multi-task deep learning. Comput Methods Programs Biomed. May 2023;233:107466. [doi: 10.1016/j.cmpb.2023.107466] [Medline: 36907040]

61. Li L, Zhang J, Zhe X, et al. Prediction of histopathologic grades of bladder cancer with radiomics based on MRI: comparison with traditional MRI. Urol Oncol. Jun 2024;42(6):176. [doi: 10.1016/j.urolonc.2024.02.008] [Medline: 38556403]

62. Chen L, Zhang G, Xu L, et al. Preoperative CT features to predict risk stratification of non-muscle invasive bladder cancer. Abdom Radiol (NY). Feb 2023;48(2):659-668. [doi: 10.1007/s00261-022-03730-y] [Medline: 36454277]

63. Xu X, Liu Y, Zhang X, et al. Preoperative prediction of muscular invasiveness of bladder cancer with radiomic features on conventional MRI and its high-order derivative maps. Abdom Radiol (NY). Jul 2017;42(7):1896-1905. [doi: 10.1007/s00261-017-1079-6] [Medline: 28217825]

64. Xu X, Zhang X, Tian Q, et al. Quantitative identification of nonmuscle‐invasive and muscle‐invasive bladder carcinomas: a multiparametric MRI radiomics analysis. J Magn Reson Imaging. May 2019;49(5):1489-1498. URL: <https://onlinelibrary.wiley.com/toc/15222586/49/5> [Accessed 2026-04-26] [doi: 10.1002/jmri.26327] [Medline: 30252978]

65. Gao RZ, Wen R, Wen DY, et al. Radiomics analysis based on ultrasound images to distinguish the tumor stage and pathological grade of bladder cancer. J Ultrasound Med. Dec 2021;40(12):2685-2697. [doi: 10.1002/jum.15659] [Medline: 33615528]

66. Wang H, Hu D, Yao H, et al. Radiomics analysis of multiparametric MRI for the preoperative evaluation of pathological grade in bladder cancer tumors. Eur Radiol. Nov 2019;29(11):6182-6190. [doi: 10.1007/s00330-019-06222-8] [Medline: 31016445]

67. Zhang X, Xu X, Tian Q, et al. Radiomics assessment of bladder cancer grade using texture features from diffusion-weighted imaging. J Magn Reson Imaging. Nov 2017;46(5):1281-1288. URL: <https://onlinelibrary.wiley.com/toc/15222586/46/5> [Accessed 2026-04-26] [doi: 10.1002/jmri.25669] [Medline: 28199039]

68. Gresser E, Woźnicki P, Messmer K, et al. Radiomics signature using manual versus automated segmentation for lymph node staging of bladder cancer. Eur Urol Focus. Jan 2023;9(1):145-153. [doi: 10.1016/j.euf.2022.08.015] [Medline: 36115774]

69. Tong Y, Udupa JK, Wang C, et al. Radiomics-guided therapy for bladder cancer: using an optimal biomarker approach to determine extent of bladder cancer invasion from t2-weighted magnetic resonance images. Adv Radiat Oncol. 2018;3(3):331-338. [doi: 10.1016/j.adro.2018.04.011] [Medline: 30202802]

70. Tao T, Chen Y, Shang Y, He J, Hao J. SMMF: a self-attention-based multi-parametric MRI feature fusion framework for the diagnosis of bladder cancer grading. Front Oncol. 2024;14:1337186. [doi: 10.3389/fonc.2024.1337186] [Medline: 38515574]

71. Liu Y, Xu X, Wang H, et al. The additional value of tri-parametric MRI in identifying muscle-invasive status in bladder cancer. Acad Radiol. Jan 2023;30(1):64-76. [doi: 10.1016/j.acra.2022.04.014] [Medline: 35676179]

72. Liu D, Wang S, Wang J. The effect of CT high-resolution imaging diagnosis based on deep residual network on the pathology of bladder cancer classification and staging. Comput Methods Programs Biomed. Mar 2022;215:106635. [doi: 10.1016/j.cmpb.2022.106635] [Medline: 35063711]

73. Razik A, Das CJ, Sharma R, et al. Utility of first order MRI-Texture analysis parameters in the prediction of histologic grade and muscle invasion in urinary bladder cancer: a preliminary study. Br J Radiol. Jun 1, 2021;94(1122):20201114. [doi: 10.1259/bjr.20201114] [Medline: 33882245]

74. Peng J, Tang Z, Li T, Pan X, Feng L, Long L. Contrast-enhanced computed tomography-based radiomics nomogram for predicting HER2 status in urothelial bladder carcinoma. Front Oncol. 2024;14:1427122. [doi: 10.3389/fonc.2024.1427122] [Medline: 39206159]

75. Xiong S, Fu Z, Deng Z, et al. Machine learning‐based CT radiomics enhances bladder cancer staging predictions: a comparative study of clinical, radiomics, and combined models. Med Phys. Sep 2024;51(9):5965-5977. URL: <https://aapm.onlinelibrary.wiley.com/toc/24734209/51/9> [doi: 10.1002/mp.17288] [Medline: 38977273]

76. Feng S, Zhou D, Li Y, et al. Prediction of Ki-67 expression in bladder cancer based on CT radiomics nomogram. Front Oncol. 2024;14:1276526. [doi: 10.3389/fonc.2024.1276526] [Medline: 38482209]

77. Wei Z, Bai X, Xv Y, et al. A radiomics-based interpretable machine learning model to predict the HER2 status in bladder cancer: a multicenter study. Insights Imaging. Oct 28, 2024;15(1):262. [doi: 10.1186/s13244-024-01840-3] [Medline: 39466475]

78. Cai L, Yang X, Yu J, et al. Deep learning on T2WI to predict the muscle-invasive bladder cancer: a multi-center clinical study. Sci Rep. Mar 22, 2025;15(1):9942. [doi: 10.1038/s41598-024-82909-3] [Medline: 40121216]

79. Du C, Wei W, Hu M, et al. Multi-DECT image-based interpretable model incorporating habitat radiomics and vision transformer deep learning for preoperative prediction of muscle invasion in bladder cancer. Acad Radiol. Dec 2025;32(12):7204-7214. [doi: 10.1016/j.acra.2025.08.018] [Medline: 40887351]

80. Du Y, Li H, Sui Y, et al. Habitat-based radiomic model for predicting muscle invasion in bladder cancer: a multi-center study using enhanced-CT and machine learning. Med Phys. Aug 2025;52(8):e18021. [doi: 10.1002/mp.18021] [Medline: 40781767]

81. He X, Chen Y, Zhou S, et al. A machine learning model based on multi-phase contrast-enhanced CT for the preoperative prediction of the muscle-invasive status of bladder cancer. Curr Med Imaging. 2025;21:e15734056377754. [doi: 10.2174/0115734056377754250304040058] [Medline: 40103464]

82. Hu M, Zhang J, Cheng Q, et al. Multi-DECT image-based intratumoral and peritumoral radiomics for preoperative prediction of muscle invasion in bladder cancer. Acad Radiol. Jan 2025;32(1):287-297. [doi: 10.1016/j.acra.2024.08.010] [Medline: 39168722]

83. Yu J, Cai L, Chen C, et al. Multi-path neural network based on mp-MRI for predicting muscle-invasive bladder cancer. Intell Data Anal. Nov 2025;29(6):1568-1581. [doi: 10.1177/1088467X241313324]

84. Zhou Q, Ma L, Yu Y, et al. Development of a radiomics and clinical feature-based nomogram for preoperative prediction of pathological grade in bladder cancer. Front Oncol. 2025;15:1661979. [doi: 10.3389/fonc.2025.1661979] [Medline: 40936708]
