# Supplementary material for: Application Value of Radiomics-Based Machine Learning for Preoperative Risk Stratification of Bladder Cancer: Systematic Review and Meta-Analysis
Source: J Med Internet Res. 2026 Jun 12;28:e81084. doi: 10.2196/81084 (PMC13263024; doi:10.2196/81084)
Supplement: Multimedia Appendix 3 [file jmir-v28-e81084-s003.docx]

**
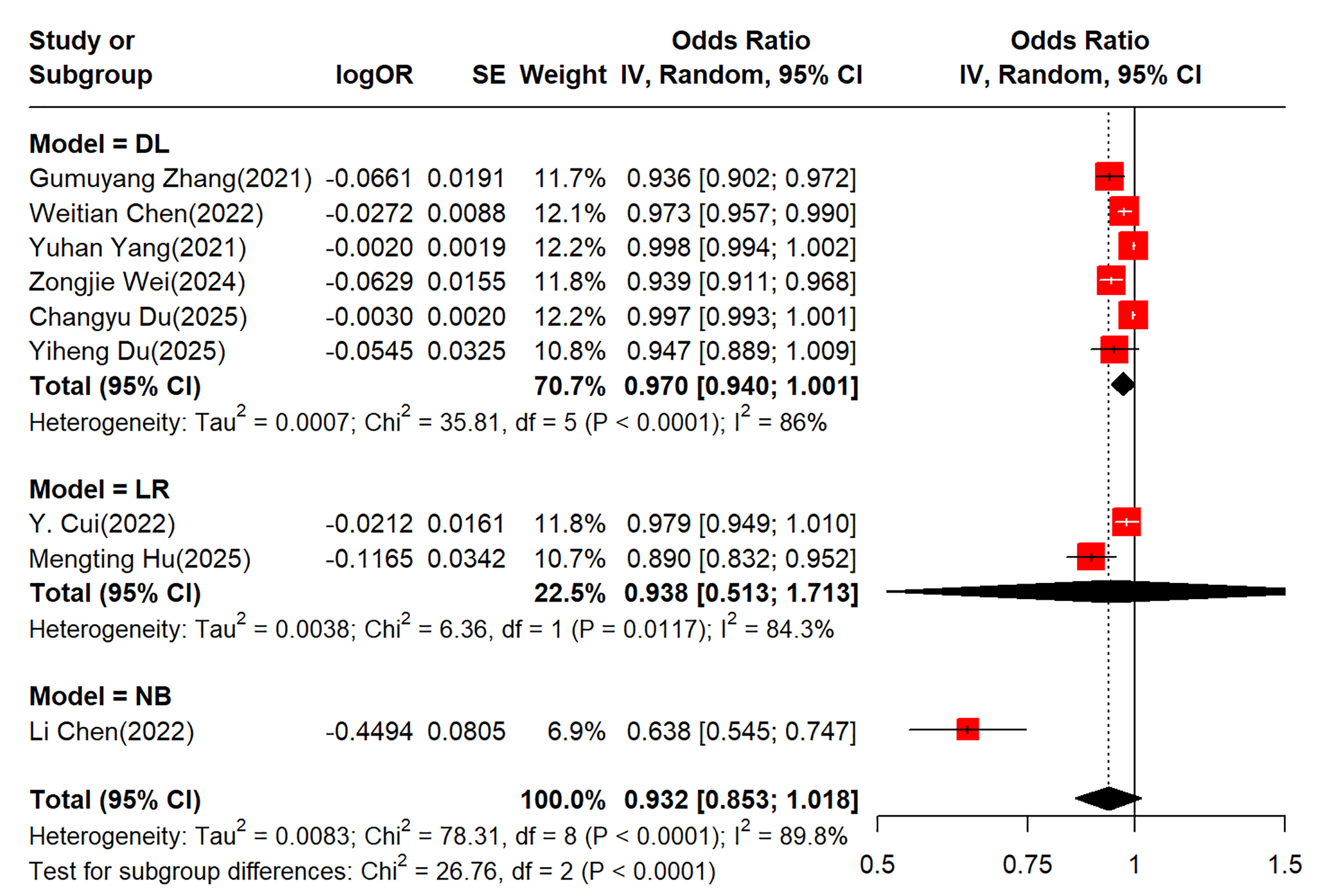
**

**Figure S1** Forest plot of the area under the curve (AUC) for the detection of muscle-invasive bladder cancer using CT radiomics-based machine learning in the training set [29,34,45,48,50,62,79,80,82].

**Reference:**

29. Zhang G, Wu Z, Xu L, et al. Deep learning on enhanced CT images can predict the muscular invasiveness of bladder cancer. Front Oncol. 2021;11:654685. [doi: 10.3389/fonc.2021.654685] [Medline: 34178641]

34. Chen W, Gong M, Zhou D, et al. CT-based deep learning radiomics signature for the preoperative prediction of the muscle-invasive status of bladder cancer. Front Oncol. 2022;12:1019749. [doi: 10.3389/fonc.2022.1019749] [Medline: 36544709]

45. Yang Y, Zou X, Wang Y, Ma X. Application of deep learning as a noninvasive tool to differentiate muscle-invasive bladder cancer and non-muscle-invasive bladder cancer with CT. Eur J Radiol. Jun 2021;139:109666. [doi: 10.1016/j.ejrad.2021.109666] [Medline: 33798819]

48. Cui Y, Sun Z, Liu X, Zhang X, Wang X. CT-based radiomics for the preoperative prediction of the muscle-invasive status of bladder cancer and comparison to radiologists’ assessment. Clin Radiol. Jun 2022;77(6):e473-e482. [doi: 10.1016/j.crad.2022.02.019] [Medline: 35367051]

50. Wei Z, Liu H, Xv Y, et al. Development and validation of a CT-based deep learning radiomics nomogram to predict muscle invasion in bladder cancer. Heliyon. Jan 30, 2024;10(2):e24878. [doi: 10.1016/j.heliyon.2024.e24878] [Medline: 38304824]

62. Chen L, Zhang G, Xu L, et al. Preoperative CT features to predict risk stratification of non-muscle invasive bladder cancer. Abdom Radiol (NY). Feb 2023;48(2):659-668. [doi: 10.1007/s00261-022-03730-y] [Medline: 36454277]

79. Du C, Wei W, Hu M, et al. Multi-DECT image-based interpretable model incorporating habitat radiomics and vision transformer deep learning for preoperative prediction of muscle invasion in bladder cancer. Acad Radiol. Dec 2025;32(12):7204-7214. [doi: 10.1016/j.acra.2025.08.018] [Medline: 40887351]

80. Du Y, Li H, Sui Y, et al. Habitat-based radiomic model for predicting muscle invasion in bladder cancer: a multi-center study using enhanced-CT and machine learning. Med Phys. Aug 2025;52(8):e18021. [doi: 10.1002/mp.18021] [Medline: 40781767]

82. Hu M, Zhang J, Cheng Q, et al. Multi-DECT image-based intratumoral and peritumoral radiomics for preoperative prediction of muscle invasion in bladder cancer. Acad Radiol. Jan 2025;32(1):287-297. [doi: 10.1016/j.acra.2024.08.010] [Medline: 39168722]

**
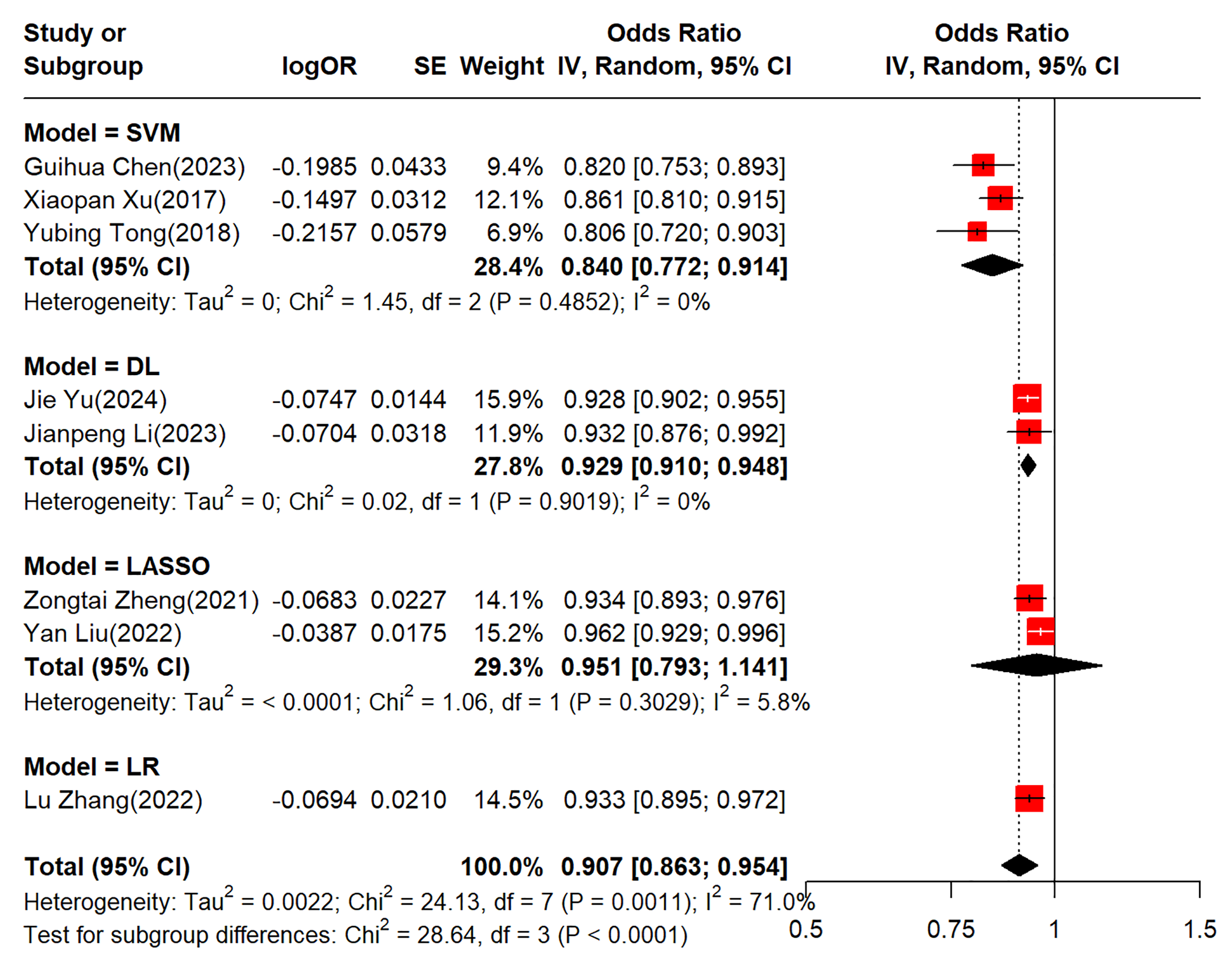
**

**Figure S2** Forest plot of the area under the curve (AUC) for the detection of muscle-invasive bladder cancer using MRI radiomics-based machine learning in the training set [41,42,46,57,60,63,69,71].

**Reference:**

41. Chen G, Fan X, Wang T, et al. A machine learning model based on MRI for the preoperative prediction of bladder cancer invasion depth. Eur Radiol. Dec 2023;33(12):8821-8832. [doi: 10.1007/s00330-023-09960-y] [Medline: 37470826]

42. Yu J, Cai L, Chen C, et al. A novel predict method for muscular invasion of bladder cancer based on 3D mp-MRI feature fusion. Phys Med Biol. Feb 22, 2024;69(5). [doi: 10.1088/1361-6560/ad25c7] [Medline: 38306973]

46. Zheng Z, Xu F, Gu Z, et al. Combining Multiparametric MRI Radiomics Signature With the Vesical Imaging-Reporting and Data System (VI-RADS) Score to Preoperatively Differentiate Muscle Invasion of Bladder Cancer. Front Oncol. 2021;11:619893. [doi: 10.3389/fonc.2021.619893] [Medline: 34055600]

57. Zhang L, Li X, Yang L, et al. Multi‐sequence and multi‐regional MRI ‐based radiomics nomogram for the preoperative assessment of muscle invasion in bladder cancer. J Magn Reson Imaging. Jul 2023;58(1):258-269. URL: <https://onlinelibrary.wiley.com/toc/15222586/58/1> [doi: 10.1002/jmri.28498] [Medline: 36300676]

60. Li J, Qiu Z, Cao K, et al. Predicting muscle invasion in bladder cancer based on MRI: a comparison of radiomics, and single-task and multi-task deep learning. Comput Methods Programs Biomed. May 2023;233:107466. [doi: 10.1016/j.cmpb.2023.107466] [Medline: 36907040]

63. Xu X, Liu Y, Zhang X, et al. Preoperative prediction of muscular invasiveness of bladder cancer with radiomic features on conventional MRI and its high-order derivative maps. Abdom Radiol (NY). Jul 2017;42(7):1896-1905. [doi: 10.1007/s00261-017-1079-6] [Medline: 28217825]

69. Tong Y, Udupa JK, Wang C, et al. Radiomics-guided therapy for bladder cancer: using an optimal biomarker approach to determine extent of bladder cancer invasion from t2-weighted magnetic resonance images. Adv Radiat Oncol. 2018;3(3):331-338. [doi: 10.1016/j.adro.2018.04.011] [Medline: 30202802]

71. Liu Y, Xu X, Wang H, et al. The additional value of tri-parametric MRI in identifying muscle-invasive status in bladder cancer. Acad Radiol. Jan 2023;30(1):64-76. [doi: 10.1016/j.acra.2022.04.014] [Medline: 35676179]

**
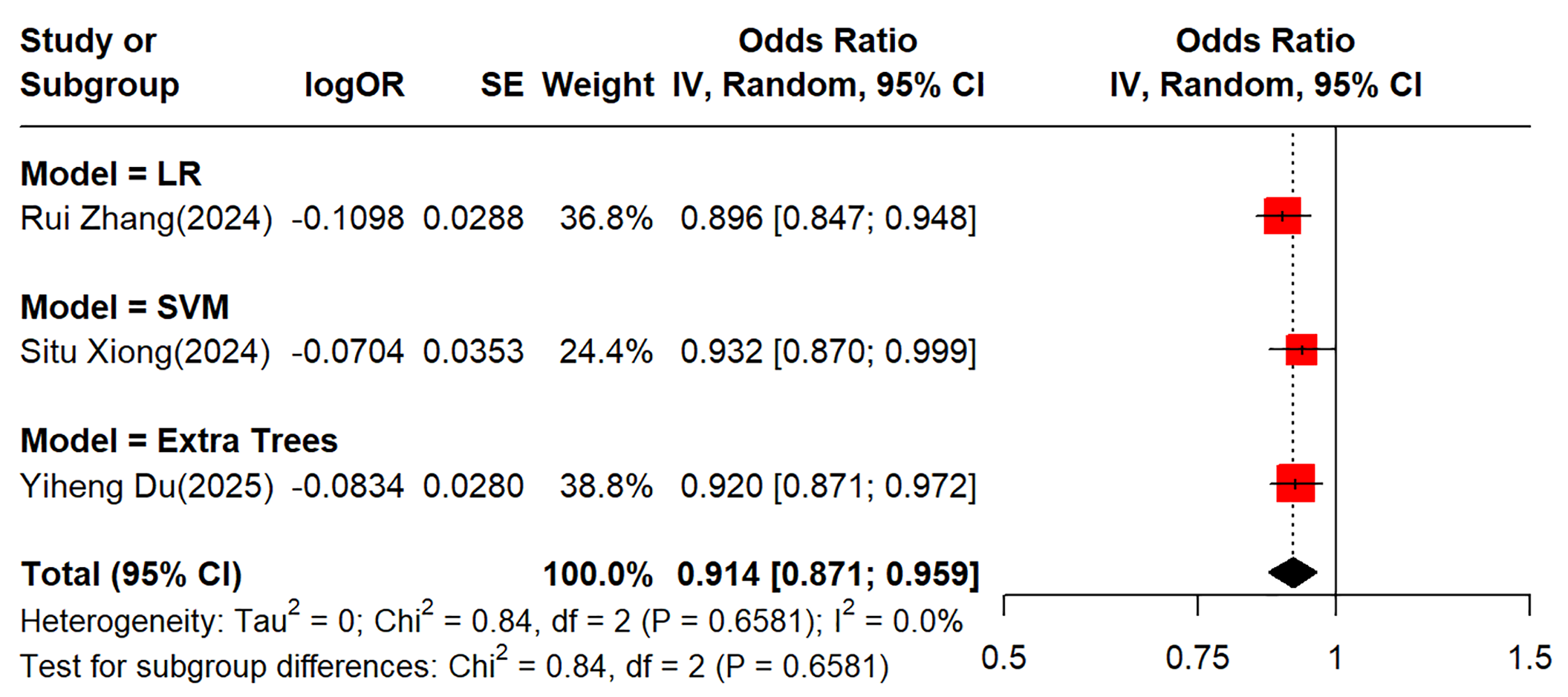
**

**Figure S3** Forest plot of the area under the curve (AUC) for the detection of muscle-invasive bladder cancer using machine learning models based on CT radiomics combined with clinical features in the training set [28,75,80].

**Reference:**

28. Zhang R, Jia S, Zhai L, Wu F, Zhang S, Li F. Predicting preoperative muscle invasion status for bladder cancer using computed tomography-based radiomics nomogram. BMC Med Imaging. Apr 27, 2024;24(1):98. [doi: 10.1186/s12880-024-01276-7] [Medline: 38678222]

75. Xiong S, Fu Z, Deng Z, et al. Machine learning‐based CT radiomics enhances bladder cancer staging predictions: a comparative study of clinical, radiomics, and combined models. Med Phys. Sep 2024;51(9):5965-5977. URL: <https://aapm.onlinelibrary.wiley.com/toc/24734209/51/9> [doi: 10.1002/mp.17288] [Medline: 38977273]

80. Du Y, Li H, Sui Y, et al. Habitat-based radiomic model for predicting muscle invasion in bladder cancer: a multi-center study using enhanced-CT and machine learning. Med Phys. Aug 2025;52(8):e18021. [doi: 10.1002/mp.18021] [Medline: 40781767]

**
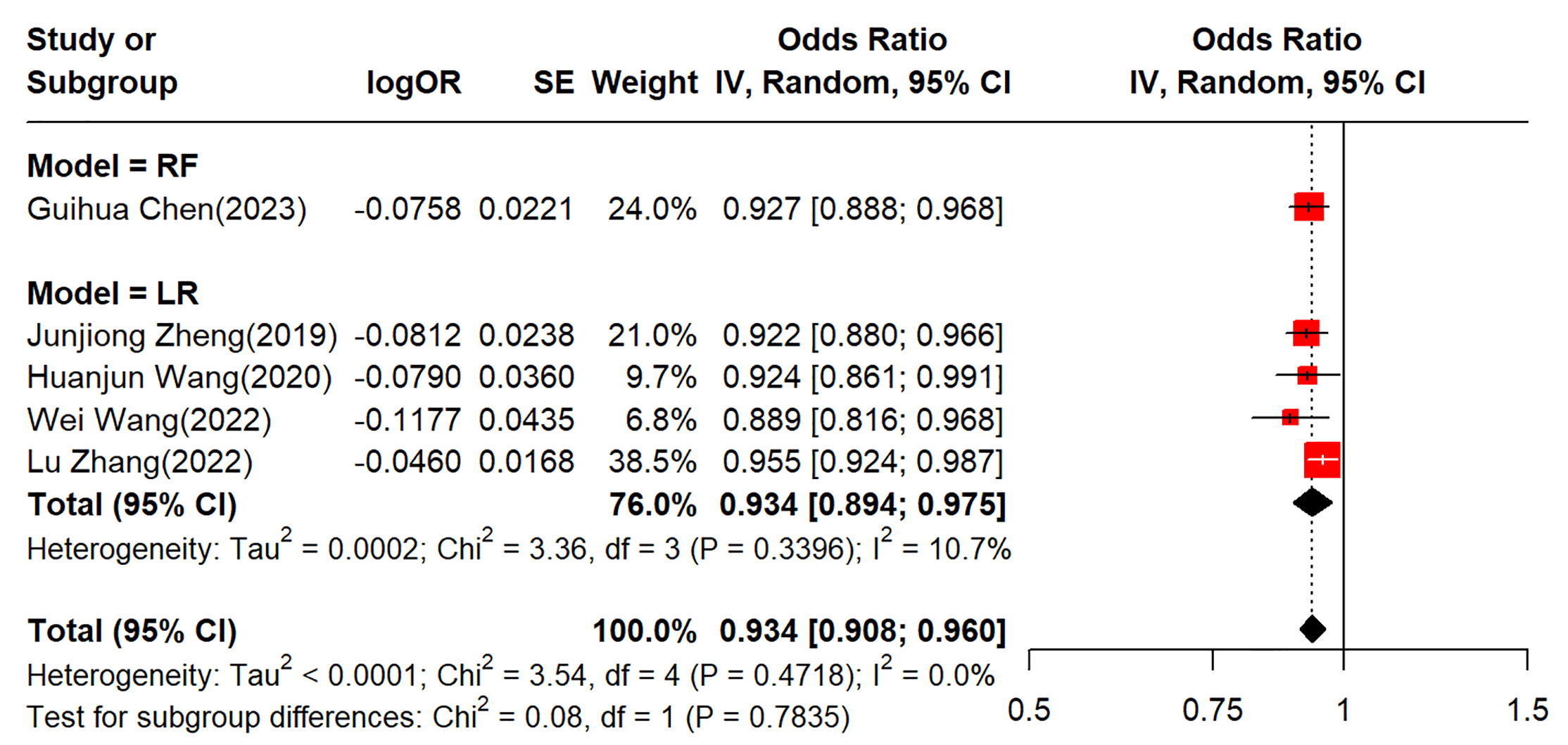
**

**Figure S4** Forest plot of the area under the curve (AUC) for the detection of muscle-invasive bladder cancer using machine learning models based on MRI radiomics combined with clinical features in the training set [41,51,52,54,57].

**Reference:**

41. Chen G, Fan X, Wang T, et al. A machine learning model based on MRI for the preoperative prediction of bladder cancer invasion depth. Eur Radiol. Dec 2023;33(12):8821-8832. [doi: 10.1007/s00330-023-09960-y] [Medline: 37470826]

51. Zheng J, Kong J, Wu S, et al. Development of a noninvasive tool to preoperatively evaluate the muscular invasiveness of bladder cancer using a radiomics approach. Cancer. Dec 15, 2019;125(24):4388-4398. [doi: 10.1002/cncr.32490] [Medline: 31469418]

52. Wang H, Xu X, Zhang X, et al. Elaboration of a multisequence MRI-based radiomics signature for the preoperative prediction of the muscle-invasive status of bladder cancer: a double-center study. Eur Radiol. Sep 2020;30(9):4816-4827. [doi: 10.1007/s00330-020-06796-8] [Medline: 32318846]

54. Wang W, Li W, Wang K, et al. Integrating radiomics with the vesical imaging-reporting and data system to predict muscle invasion of bladder cancer. Urol Oncol. Jun 2023;41(6):294. [doi: 10.1016/j.urolonc.2022.10.024] [Medline: 36526525]

57. Zhang L, Li X, Yang L, et al. Multi‐sequence and multi‐regional MRI ‐based radiomics nomogram for the preoperative assessment of muscle invasion in bladder cancer. J Magn Reson Imaging. Jul 2023;58(1):258-269. URL: <https://onlinelibrary.wiley.com/toc/15222586/58/1> [doi: 10.1002/jmri.28498] [Medline: 36300676]

**
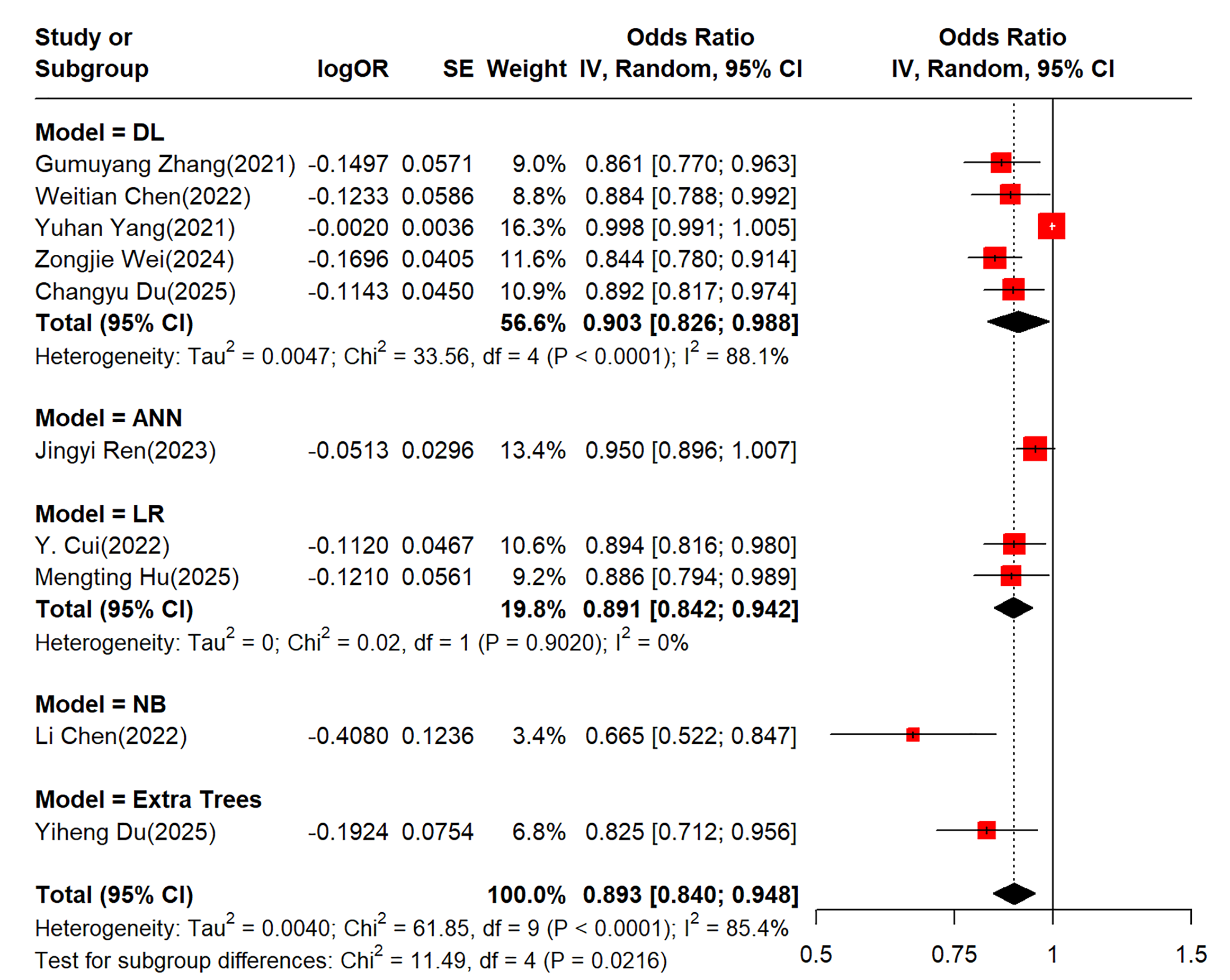
Figure S5** Forest plot of the area under the curve (AUC) for the detection of muscle-invasive bladder cancer using CT radiomics-based machine learning in the validation set [29,31,34,45,48,50,62,79,80,82].

**Reference:**

29. Zhang G, Wu Z, Xu L, et al. Deep learning on enhanced CT images can predict the muscular invasiveness of bladder cancer. Front Oncol. 2021;11:654685. [doi: 10.3389/fonc.2021.654685] [Medline: 34178641]

31. Ren J, Gu H, Zhang N, Chen W. Preoperative CT-based radiomics for diagnosing muscle invasion of bladder cancer. Egypt J Radiol Nucl Med. 2023;54(1):131. [doi: 10.1186/s43055-023-01044-7]

34. Chen W, Gong M, Zhou D, et al. CT-based deep learning radiomics signature for the preoperative prediction of the muscle-invasive status of bladder cancer. Front Oncol. 2022;12:1019749. [doi: 10.3389/fonc.2022.1019749] [Medline: 36544709]

45. Yang Y, Zou X, Wang Y, Ma X. Application of deep learning as a noninvasive tool to differentiate muscle-invasive bladder cancer and non-muscle-invasive bladder cancer with CT. Eur J Radiol. Jun 2021;139:109666. [doi: 10.1016/j.ejrad.2021.109666] [Medline: 33798819]

48. Cui Y, Sun Z, Liu X, Zhang X, Wang X. CT-based radiomics for the preoperative prediction of the muscle-invasive status of bladder cancer and comparison to radiologists’ assessment. Clin Radiol. Jun 2022;77(6):e473-e482. [doi: 10.1016/j.crad.2022.02.019] [Medline: 35367051]

50. Wei Z, Liu H, Xv Y, et al. Development and validation of a CT-based deep learning radiomics nomogram to predict muscle invasion in bladder cancer. Heliyon. Jan 30, 2024;10(2):e24878. [doi: 10.1016/j.heliyon.2024.e24878] [Medline: 38304824]

62. Chen L, Zhang G, Xu L, et al. Preoperative CT features to predict risk stratification of non-muscle invasive bladder cancer. Abdom Radiol (NY). Feb 2023;48(2):659-668. [doi: 10.1007/s00261-022-03730-y] [Medline: 36454277]

79. Du C, Wei W, Hu M, et al. Multi-DECT image-based interpretable model incorporating habitat radiomics and vision transformer deep learning for preoperative prediction of muscle invasion in bladder cancer. Acad Radiol. Dec 2025;32(12):7204-7214. [doi: 10.1016/j.acra.2025.08.018] [Medline: 40887351]

80. Du Y, Li H, Sui Y, et al. Habitat-based radiomic model for predicting muscle invasion in bladder cancer: a multi-center study using enhanced-CT and machine learning. Med Phys. Aug 2025;52(8):e18021. [doi: 10.1002/mp.18021] [Medline: 40781767]

82. Hu M, Zhang J, Cheng Q, et al. Multi-DECT image-based intratumoral and peritumoral radiomics for preoperative prediction of muscle invasion in bladder cancer. Acad Radiol. Jan 2025;32(1):287-297. [doi: 10.1016/j.acra.2024.08.010] [Medline: 39168722]

**
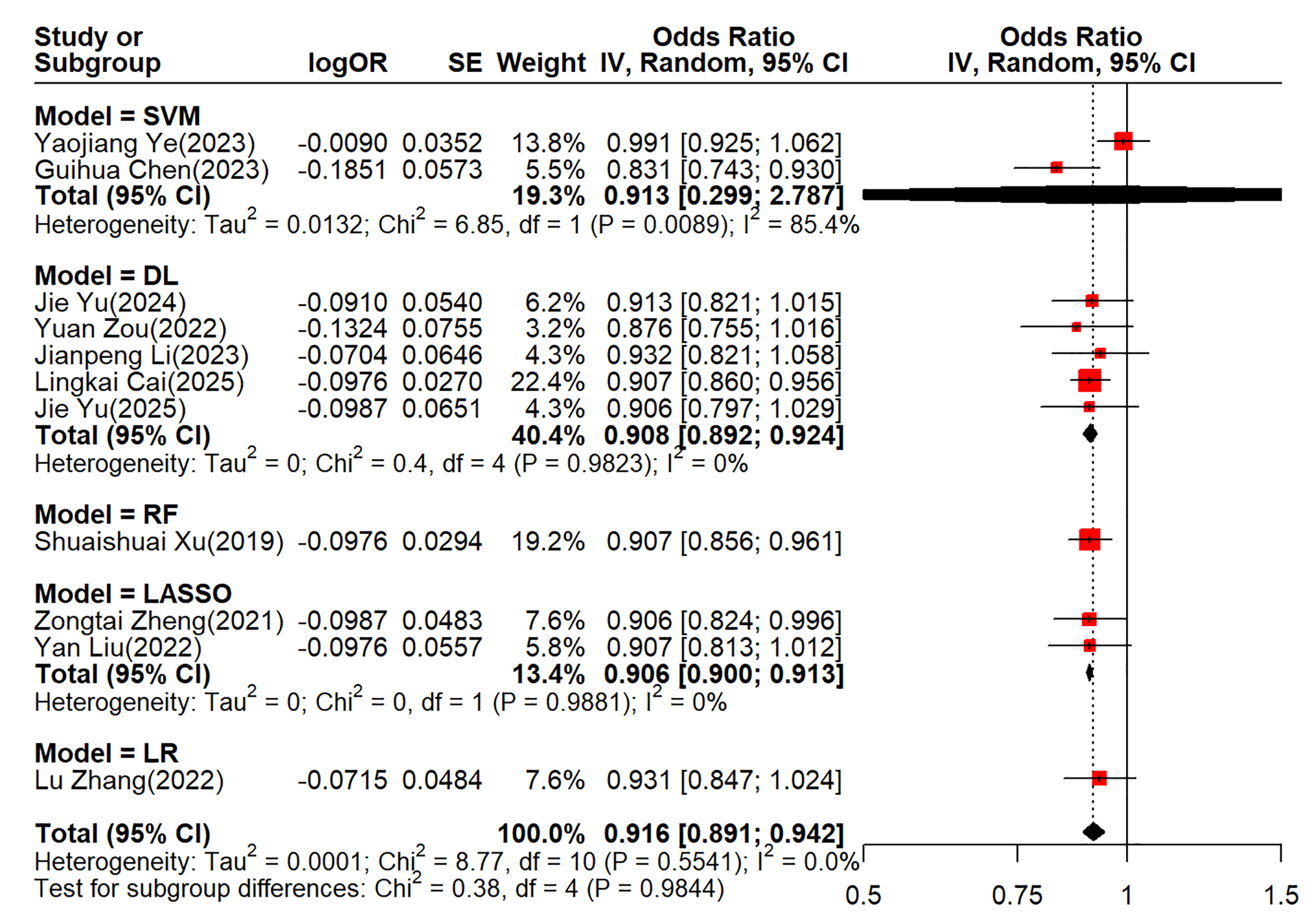
**

**Figure S6** Forest plot of the area under the curve (AUC) for the detection of muscle-invasive bladder cancer using MRI radiomics-based machine learning in the validation set [30,41-43,46,57,58,60,71,78,83].

**Reference:**

30. Ye Y, Luo Z, Qiu Z, et al. Radiomics prediction of muscle invasion in bladder cancer using semi-automatic lesion segmentation of MRI compared with manual segmentation. Bioengineering (Basel). Nov 25, 2023;10(12):1355. [doi: 10.3390/bioengineering10121355] [Medline: 38135946]

41. Chen G, Fan X, Wang T, et al. A machine learning model based on MRI for the preoperative prediction of bladder cancer invasion depth. Eur Radiol. Dec 2023;33(12):8821-8832. [doi: 10.1007/s00330-023-09960-y] [Medline: 37470826]

42. Yu J, Cai L, Chen C, et al. A novel predict method for muscular invasion of bladder cancer based on 3D mp-MRI feature fusion. Phys Med Biol. Feb 22, 2024;69(5). [doi: 10.1088/1361-6560/ad25c7] [Medline: 38306973]

43. Xu S, Yao Q, Liu G, et al. Combining DWI radiomics features with transurethral resection promotes the differentiation between muscle-invasive bladder cancer and non-muscle-invasive bladder cancer. Eur Radiol. Mar 2020;30(3):1804-1812. [doi: 10.1007/s00330-019-06484-2] [Medline: 31773297]

46. Zheng Z, Xu F, Gu Z, et al. Combining Multiparametric MRI Radiomics Signature With the Vesical Imaging-Reporting and Data System (VI-RADS) Score to Preoperatively Differentiate Muscle Invasion of Bladder Cancer. Front Oncol. 2021;11:619893. [doi: 10.3389/fonc.2021.619893] [Medline: 34055600]

57. Zhang L, Li X, Yang L, et al. Multi‐sequence and multi‐regional MRI ‐based radiomics nomogram for the preoperative assessment of muscle invasion in bladder cancer. J Magn Reson Imaging. Jul 2023;58(1):258-269. URL: <https://onlinelibrary.wiley.com/toc/15222586/58/1> [doi: 10.1002/jmri.28498] [Medline: 36300676]

58. Zou Y, Cai L, Chen C, et al. Multi-task deep learning based on T2-weighted images for predicting muscular-invasive bladder cancer. Comput Biol Med. Dec 2022;151(Pt A):106219. [doi: 10.1016/j.compbiomed.2022.106219] [Medline: 36343408]

60. Li J, Qiu Z, Cao K, et al. Predicting muscle invasion in bladder cancer based on MRI: a comparison of radiomics, and single-task and multi-task deep learning. Comput Methods Programs Biomed. May 2023;233:107466. [doi: 10.1016/j.cmpb.2023.107466] [Medline: 36907040]

71. Liu Y, Xu X, Wang H, et al. The additional value of tri-parametric MRI in identifying muscle-invasive status in bladder cancer. Acad Radiol. Jan 2023;30(1):64-76. [doi: 10.1016/j.acra.2022.04.014] [Medline: 35676179]

78. Cai L, Yang X, Yu J, et al. Deep learning on T2WI to predict the muscle-invasive bladder cancer: a multi-center clinical study. Sci Rep. Mar 22, 2025;15(1):9942. [doi: 10.1038/s41598-024-82909-3] [Medline: 40121216]

83. Yu J, Cai L, Chen C, et al. Multi-path neural network based on mp-MRI for predicting muscle-invasive bladder cancer. Intell Data Anal. Nov 2025;29(6):1568-1581. [doi: 10.1177/1088467X241313324]

**
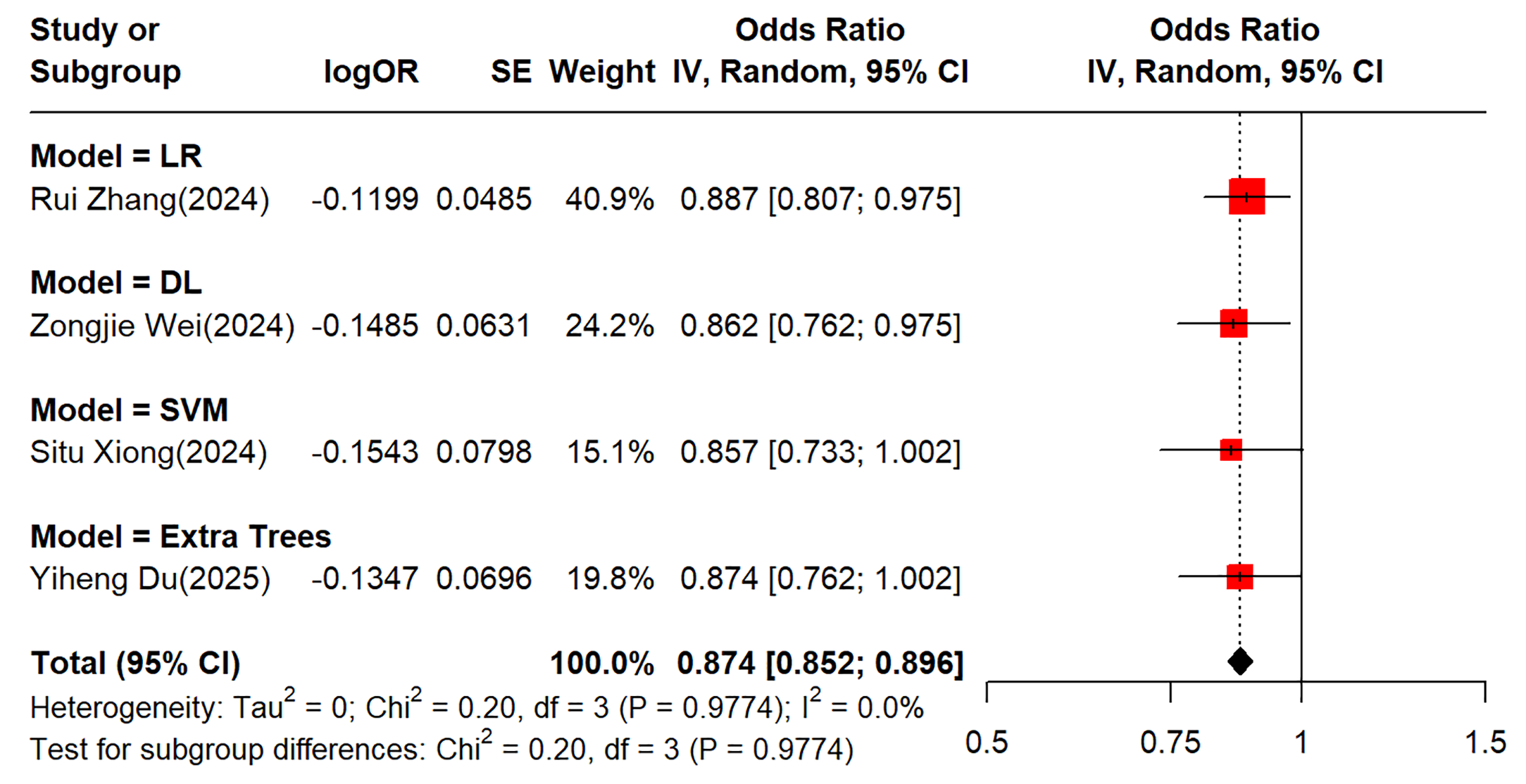
**

**Figure S7** Forest plot of the area under the curve (AUC) for the detection of muscle-invasive bladder cancer using machine learning models based on CT radiomics combined with clinical features in the validation set [28,50,75,80].

**Reference:**

28. Zhang R, Jia S, Zhai L, Wu F, Zhang S, Li F. Predicting preoperative muscle invasion status for bladder cancer using computed tomography-based radiomics nomogram. BMC Med Imaging. Apr 27, 2024;24(1):98. [doi: 10.1186/s12880-024-01276-7] [Medline: 38678222]

50. Wei Z, Liu H, Xv Y, et al. Development and validation of a CT-based deep learning radiomics nomogram to predict muscle invasion in bladder cancer. Heliyon. Jan 30, 2024;10(2):e24878. [doi: 10.1016/j.heliyon.2024.e24878] [Medline: 38304824]

75. Xiong S, Fu Z, Deng Z, et al. Machine learning‐based CT radiomics enhances bladder cancer staging predictions: a comparative study of clinical, radiomics, and combined models. Med Phys. Sep 2024;51(9):5965-5977. URL: <https://aapm.onlinelibrary.wiley.com/toc/24734209/51/9> [doi: 10.1002/mp.17288] [Medline: 38977273]

80. Du Y, Li H, Sui Y, et al. Habitat-based radiomic model for predicting muscle invasion in bladder cancer: a multi-center study using enhanced-CT and machine learning. Med Phys. Aug 2025;52(8):e18021. [doi: 10.1002/mp.18021] [Medline: 40781767]

**
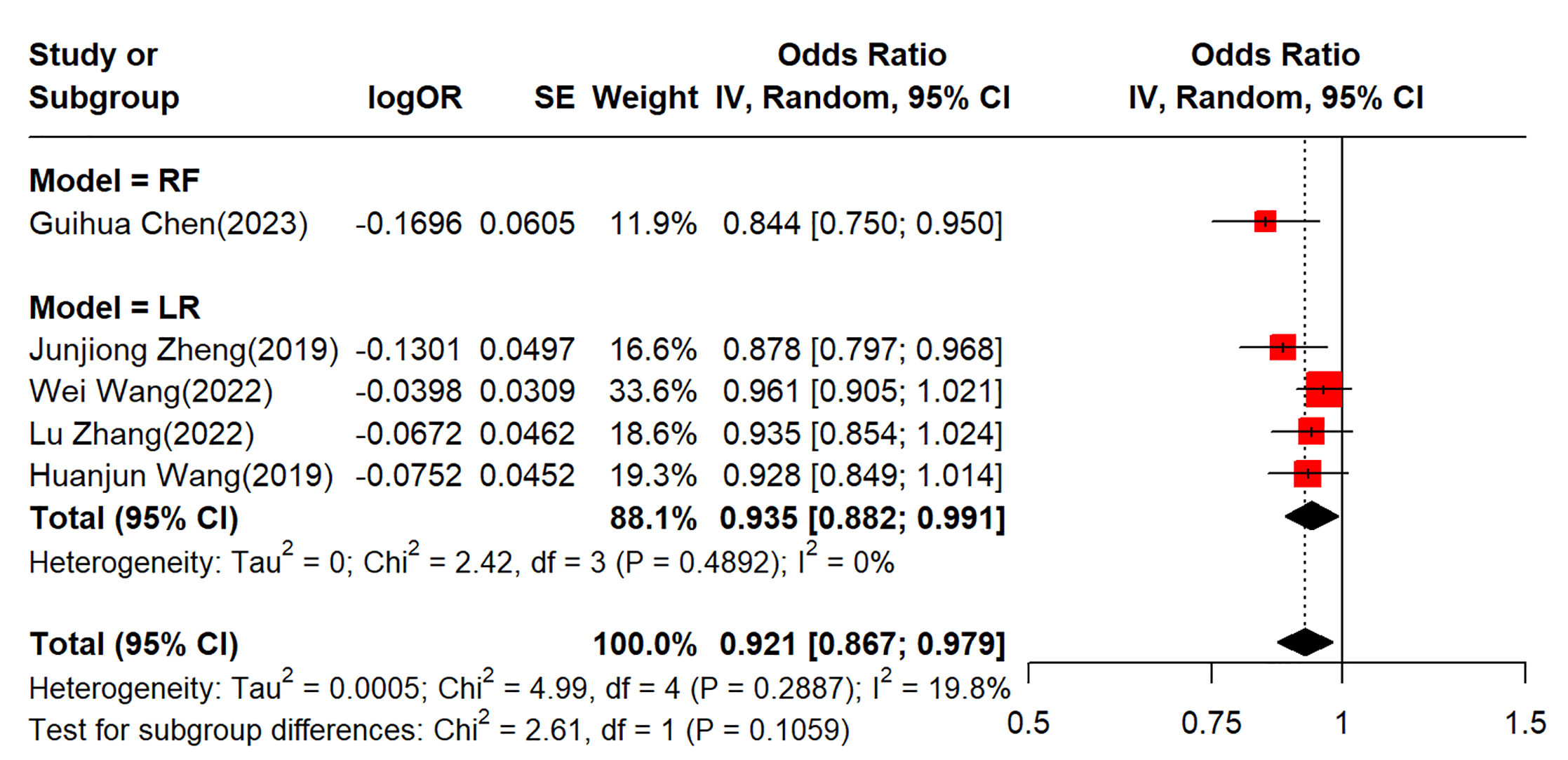
**

**Figure S8** Forest plot of the area under the curve (AUC) for the detection of muscle-invasive bladder cancer using machine learning models based on MRI radiomics combined with clinical features in the validation set [41,51,54,57,66].

**Reference:**

41. Chen G, Fan X, Wang T, et al. A machine learning model based on MRI for the preoperative prediction of bladder cancer invasion depth. Eur Radiol. Dec 2023;33(12):8821-8832. [doi: 10.1007/s00330-023-09960-y] [Medline: 37470826]

51. Zheng J, Kong J, Wu S, et al. Development of a noninvasive tool to preoperatively evaluate the muscular invasiveness of bladder cancer using a radiomics approach. Cancer. Dec 15, 2019;125(24):4388-4398. [doi: 10.1002/cncr.32490] [Medline: 31469418]

54. Wang W, Li W, Wang K, et al. Integrating radiomics with the vesical imaging-reporting and data system to predict muscle invasion of bladder cancer. Urol Oncol. Jun 2023;41(6):294. [doi: 10.1016/j.urolonc.2022.10.024] [Medline: 36526525]

57. Zhang L, Li X, Yang L, et al. Multi‐sequence and multi‐regional MRI ‐based radiomics nomogram for the preoperative assessment of muscle invasion in bladder cancer. J Magn Reson Imaging. Jul 2023;58(1):258-269. URL: <https://onlinelibrary.wiley.com/toc/15222586/58/1> [doi: 10.1002/jmri.28498] [Medline: 36300676]

66. Wang H, Hu D, Yao H, et al. Radiomics analysis of multiparametric MRI for the preoperative evaluation of pathological grade in bladder cancer tumors. Eur Radiol. Nov 2019;29(11):6182-6190. [doi: 10.1007/s00330-019-06222-8] [Medline: 31016445]

**
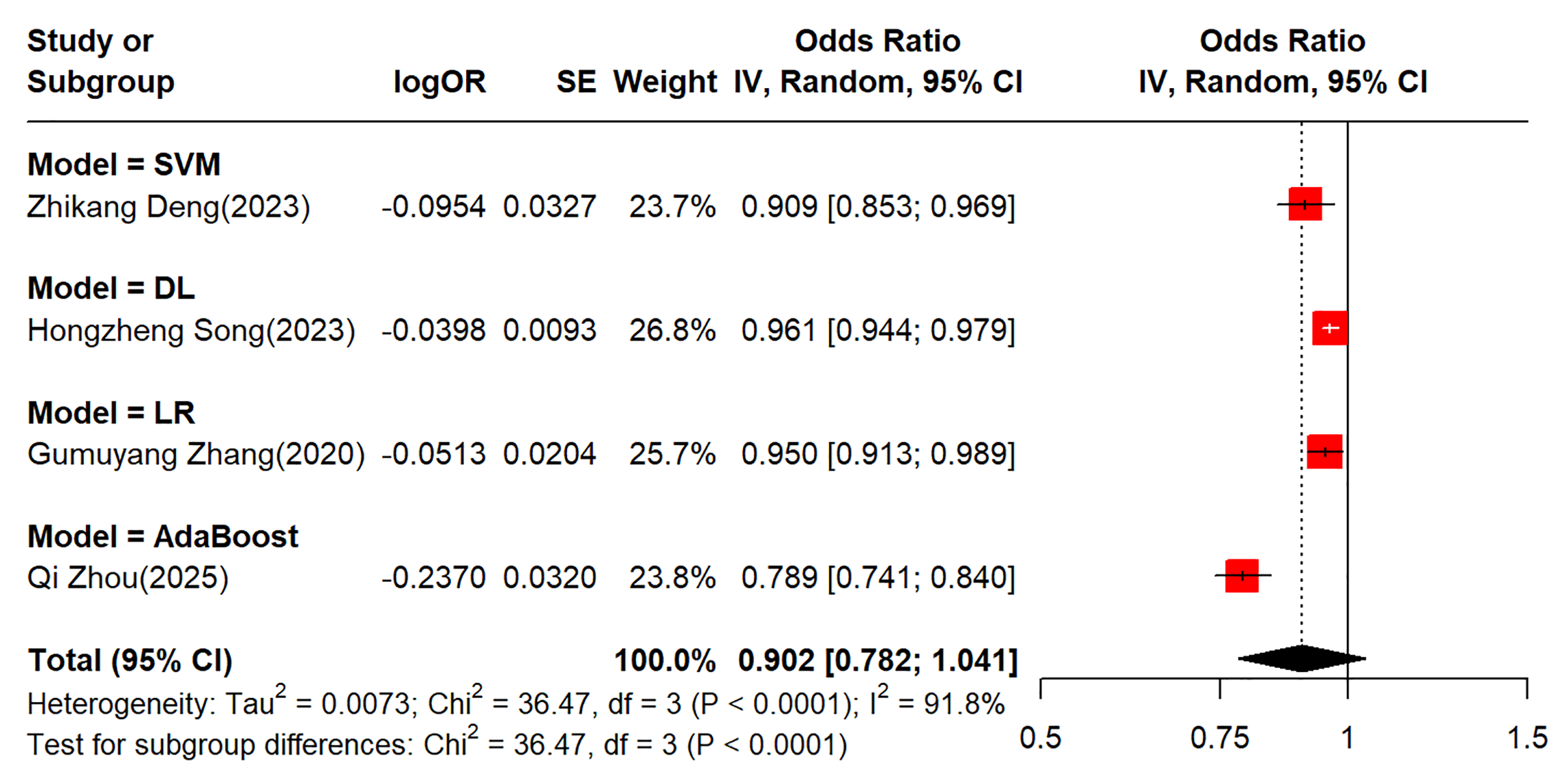
Figure S9** Forest plot of the area under the curve (AUC) for the diagnosis of high-grade tumors using CT radiomics-based machine learning in the training set [40,47,49,84].

**Reference:**

40. Deng Z, Dong W, Xiong S, et al. Machine learning models combining computed tomography semantic features and selected clinical variables for accurate prediction of the pathological grade of bladder cancer. Front Oncol. 2023;13:1166245. [doi: 10.3389/fonc.2023.1166245] [Medline: 37223680]

47. Song H, Yang S, Yu B, et al. CT-based deep learning radiomics nomogram for the prediction of pathological grade in bladder cancer: a multicenter study. Cancer Imaging. Sep 18, 2023;23(1):89. [doi: 10.1186/s40644-023-00609-z] [Medline: 37723572]

49. Zhang G, Xu L, Zhao L, et al. CT-based radiomics to predict the pathological grade of bladder cancer. Eur Radiol. Dec 2020;30(12):6749-6756. [doi: 10.1007/s00330-020-06893-8] [Medline: 32601949]

84. Zhou Q, Ma L, Yu Y, et al. Development of a radiomics and clinical feature-based nomogram for preoperative prediction of pathological grade in bladder cancer. Front Oncol. 2025;15:1661979. [doi: 10.3389/fonc.2025.1661979] [Medline: 40936708]

**
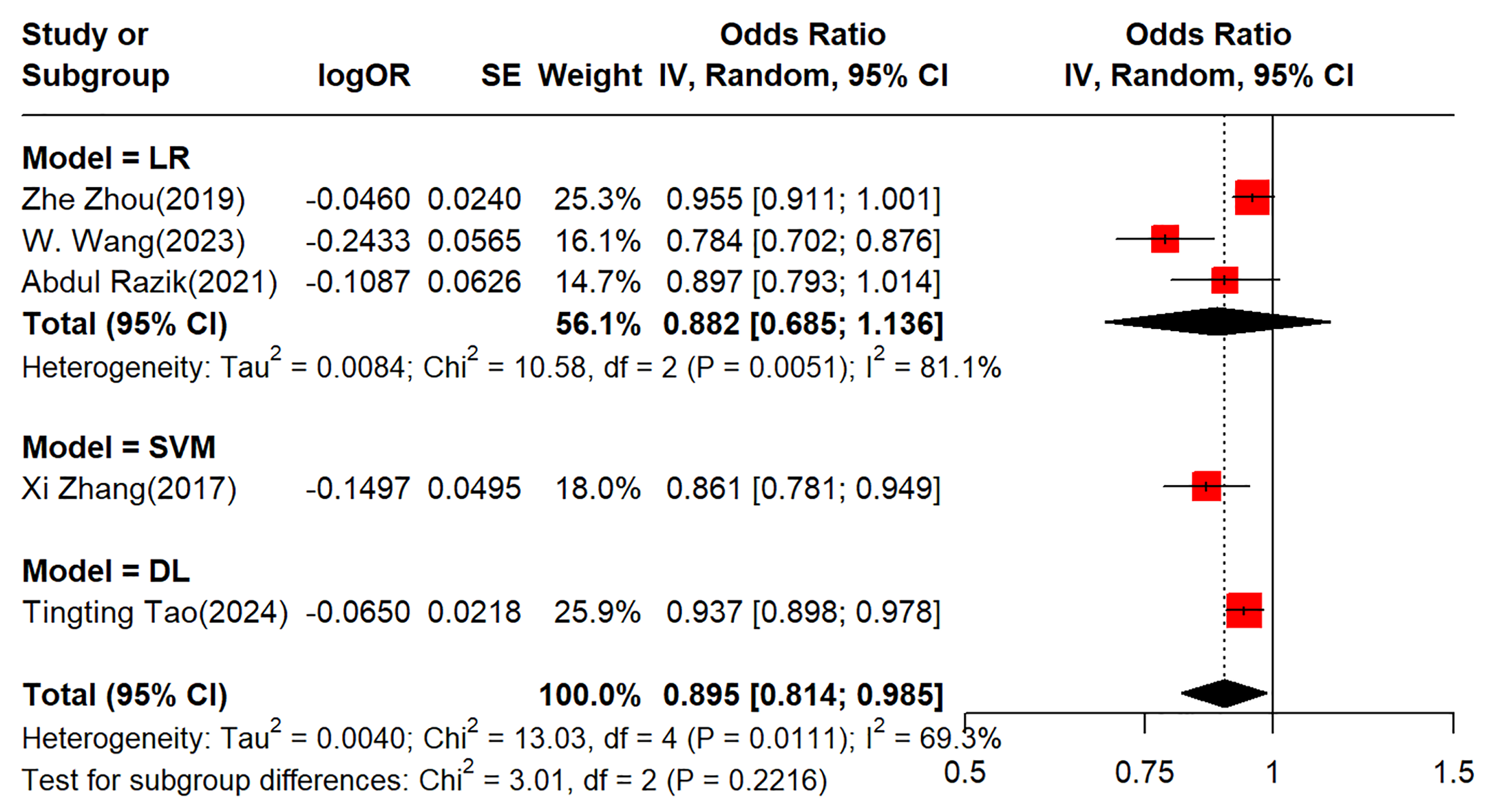
Figure S10** Forest plot of the area under the curve (AUC) for the diagnosis of high-grade tumors using MRI radiomics-based machine learning in the training set [44,56,67,70,73].

**Reference:**

44. Zhou Z, Liu L, Xue K, Ma Y, Liu J, Zhang M. Assessment of pathological grading of bladder cancer using texture features from MRI. Presented at: 2019 IEEE International Conference on Mechatronics and Automation (ICMA); Aug 4-7, 2019:1333-1337; Tianjin, China. 2019.URL: <https://ieeexplore.ieee.org/xpl/mostRecentIssue.jsp?punumber=8801712> [doi: 10.1109/ICMA.2019.8816242]

56. Wang W, Wang K, Qiu J, et al. MRI-based radiomics analysis of bladder cancer: prediction of pathological grade and histological variant. Clin Radiol. Nov 2023;78(11):e889-e897. [doi: 10.1016/j.crad.2023.07.020] [Medline: 37633748]

67. Zhang X, Xu X, Tian Q, et al. Radiomics assessment of bladder cancer grade using texture features from diffusion-weighted imaging. J Magn Reson Imaging. Nov 2017;46(5):1281-1288. URL: <https://onlinelibrary.wiley.com/toc/15222586/46/5> [Accessed 2026-04-26] [doi: 10.1002/jmri.25669] [Medline: 28199039]

70. Tao T, Chen Y, Shang Y, He J, Hao J. SMMF: a self-attention-based multi-parametric MRI feature fusion framework for the diagnosis of bladder cancer grading. Front Oncol. 2024;14:1337186. [doi: 10.3389/fonc.2024.1337186] [Medline: 38515574]

73. Razik A, Das CJ, Sharma R, et al. Utility of first order MRI-Texture analysis parameters in the prediction of histologic grade and muscle invasion in urinary bladder cancer: a preliminary study. Br J Radiol. Jun 1, 2021;94(1122):20201114. [doi: 10.1259/bjr.20201114] [Medline: 33882245]


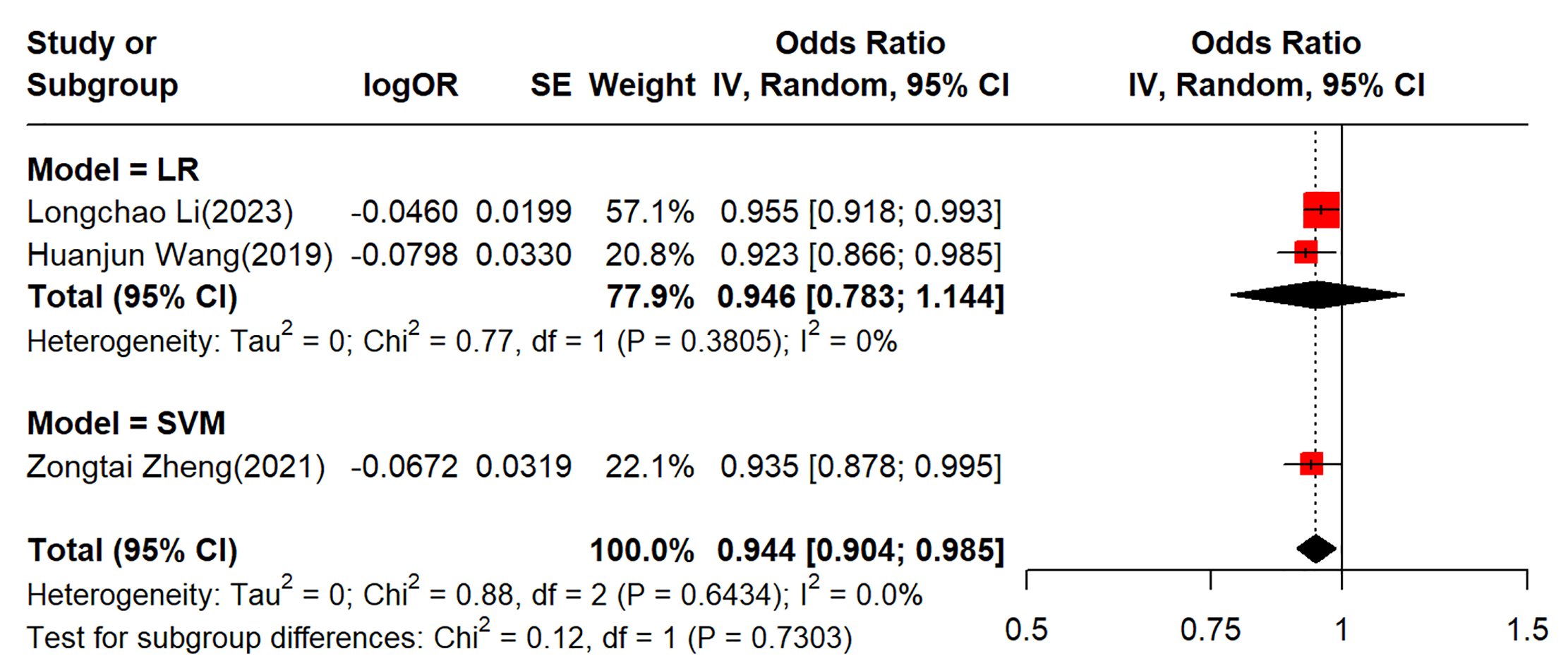


**Figure S11** Forest plot of the area under the curve (AUC) for the diagnosis of high-grade tumors using machine learning models based on MRI radiomics combined with clinical features in the training set [39,53,66].

**Reference:**

39. Li L, Zhang J, Zhe X, et al. An MRI-based radiomics nomogram in predicting histologic grade of non-muscle-invasive bladder cancer. Front Oncol. 2023;13:1025972. [doi: 10.3389/fonc.2023.1025972] [Medline: 37007156]

53. Zheng Z, Xu F, Gu Z, et al. Integrating multiparametric MRI radiomics features and the Vesical Imaging-Reporting and Data System (VI-RADS) for bladder cancer grading. Abdom Radiol (NY). Sep 2021;46(9):4311-4323. [doi: 10.1007/s00261-021-03108-6] [Medline: 33978825]

66. Wang H, Hu D, Yao H, et al. Radiomics analysis of multiparametric MRI for the preoperative evaluation of pathological grade in bladder cancer tumors. Eur Radiol. Nov 2019;29(11):6182-6190. [doi: 10.1007/s00330-019-06222-8] [Medline: 31016445]


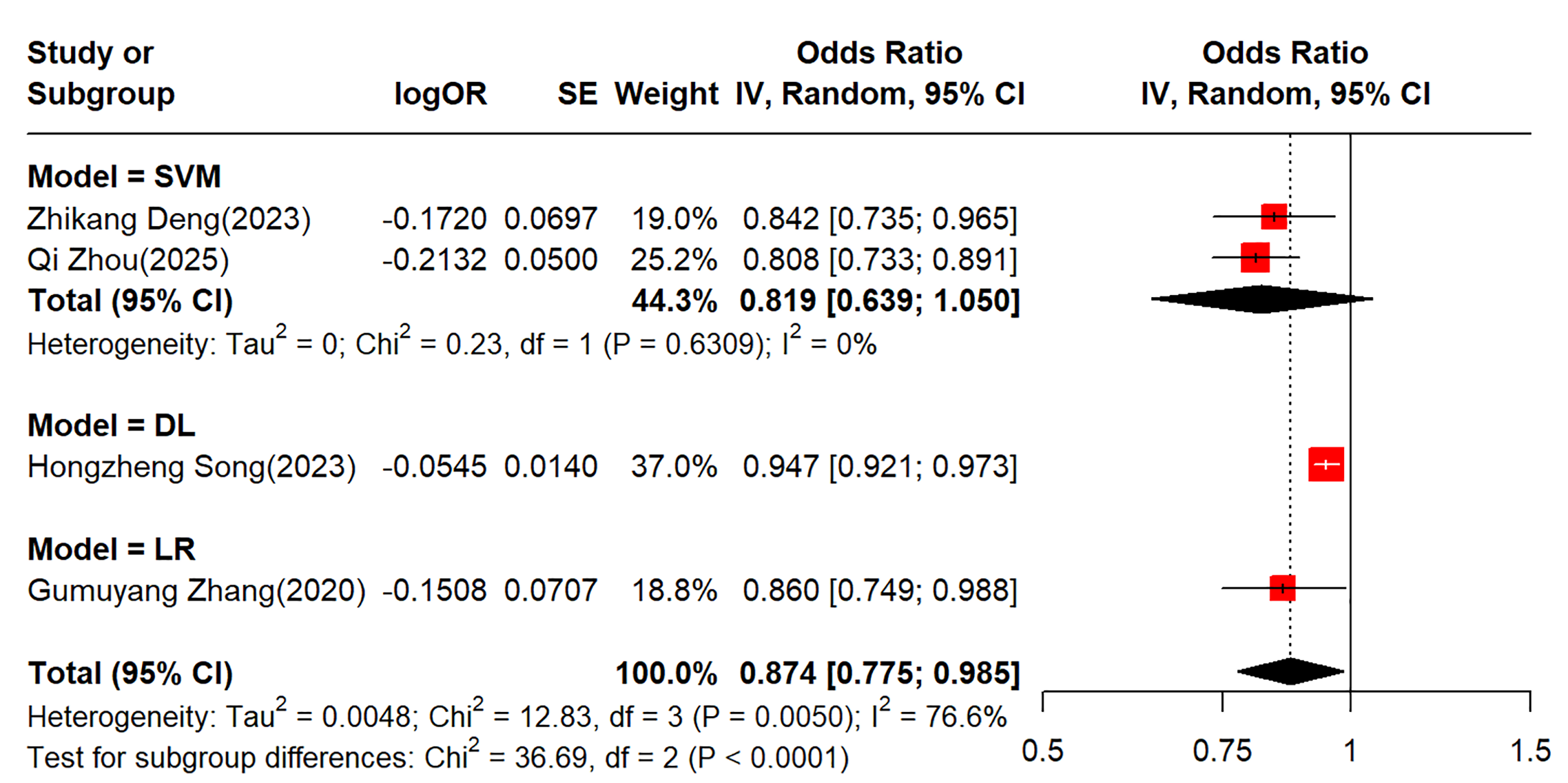


**Figure S12** Forest plot of the area under the curve (AUC) for the diagnosis of high-grade tumors using CT radiomics-based machine learning in the validation set [40,47,49,84].

**Reference:**

40. Deng Z, Dong W, Xiong S, et al. Machine learning models combining computed tomography semantic features and selected clinical variables for accurate prediction of the pathological grade of bladder cancer. Front Oncol. 2023;13:1166245. [doi: 10.3389/fonc.2023.1166245] [Medline: 37223680]

47. Song H, Yang S, Yu B, et al. CT-based deep learning radiomics nomogram for the prediction of pathological grade in bladder cancer: a multicenter study. Cancer Imaging. Sep 18, 2023;23(1):89. [doi: 10.1186/s40644-023-00609-z] [Medline: 37723572]

49. Zhang G, Xu L, Zhao L, et al. CT-based radiomics to predict the pathological grade of bladder cancer. Eur Radiol. Dec 2020;30(12):6749-6756. [doi: 10.1007/s00330-020-06893-8] [Medline: 32601949]

84. Zhou Q, Ma L, Yu Y, et al. Development of a radiomics and clinical feature-based nomogram for preoperative prediction of pathological grade in bladder cancer. Front Oncol. 2025;15:1661979. [doi: 10.3389/fonc.2025.1661979] [Medline: 40936708]

**
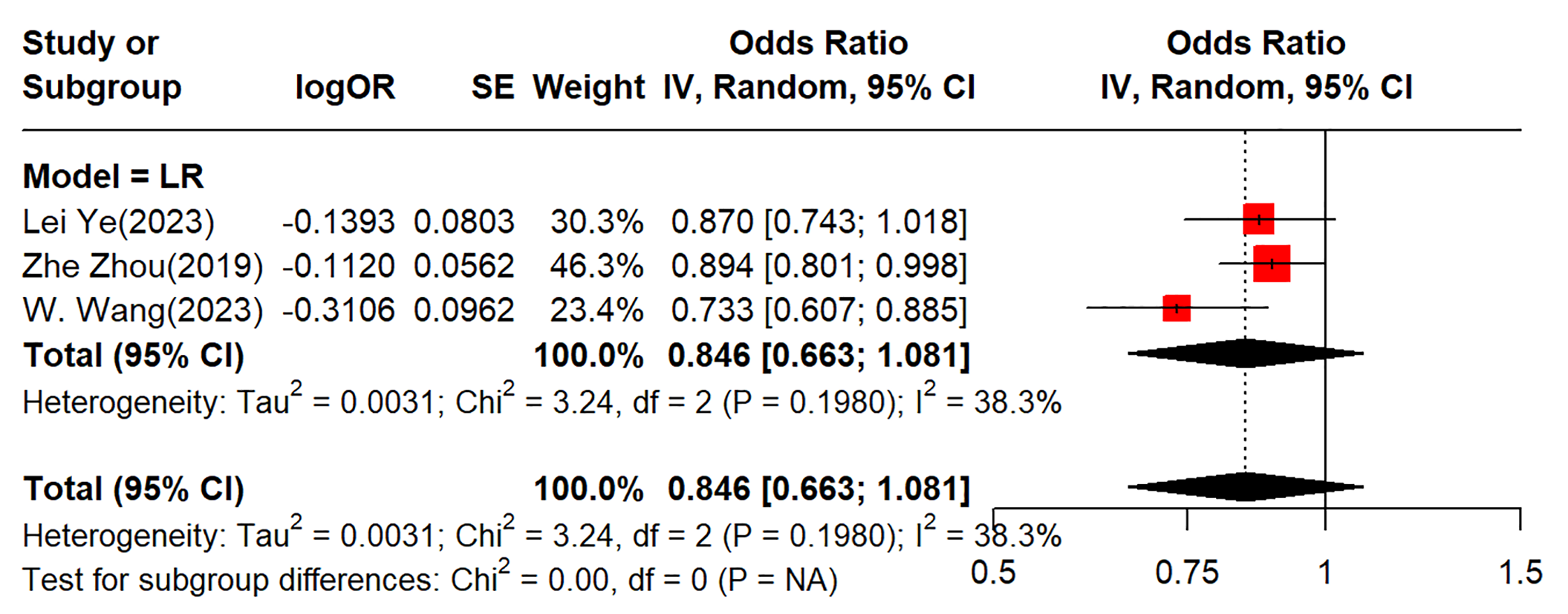
**

**Figure S13** Forest plot of the area under the curve (AUC) for the diagnosis of high-grade tumors using MRI radiomics-based machine learning in the validation set [36,44,56].

**Reference:**

36. Ye L, Wang Y, Xiang W, Yao J, Liu J, Song B. Radiomic analysis of quantitative T2 mapping and conventional MRI in predicting histologic grade of bladder cancer. J Clin Med. Sep 11, 2023;12(18):5900. [doi: 10.3390/jcm12185900] [Medline: 37762841]

44. Zhou Z, Liu L, Xue K, Ma Y, Liu J, Zhang M. Assessment of pathological grading of bladder cancer using texture features from MRI. Presented at: 2019 IEEE International Conference on Mechatronics and Automation (ICMA); Aug 4-7, 2019:1333-1337; Tianjin, China. 2019.URL: <https://ieeexplore.ieee.org/xpl/mostRecentIssue.jsp?punumber=8801712> [doi: 10.1109/ICMA.2019.8816242]

56. Wang W, Wang K, Qiu J, et al. MRI-based radiomics analysis of bladder cancer: prediction of pathological grade and histological variant. Clin Radiol. Nov 2023;78(11):e889-e897. [doi: 10.1016/j.crad.2023.07.020] [Medline: 37633748]

**
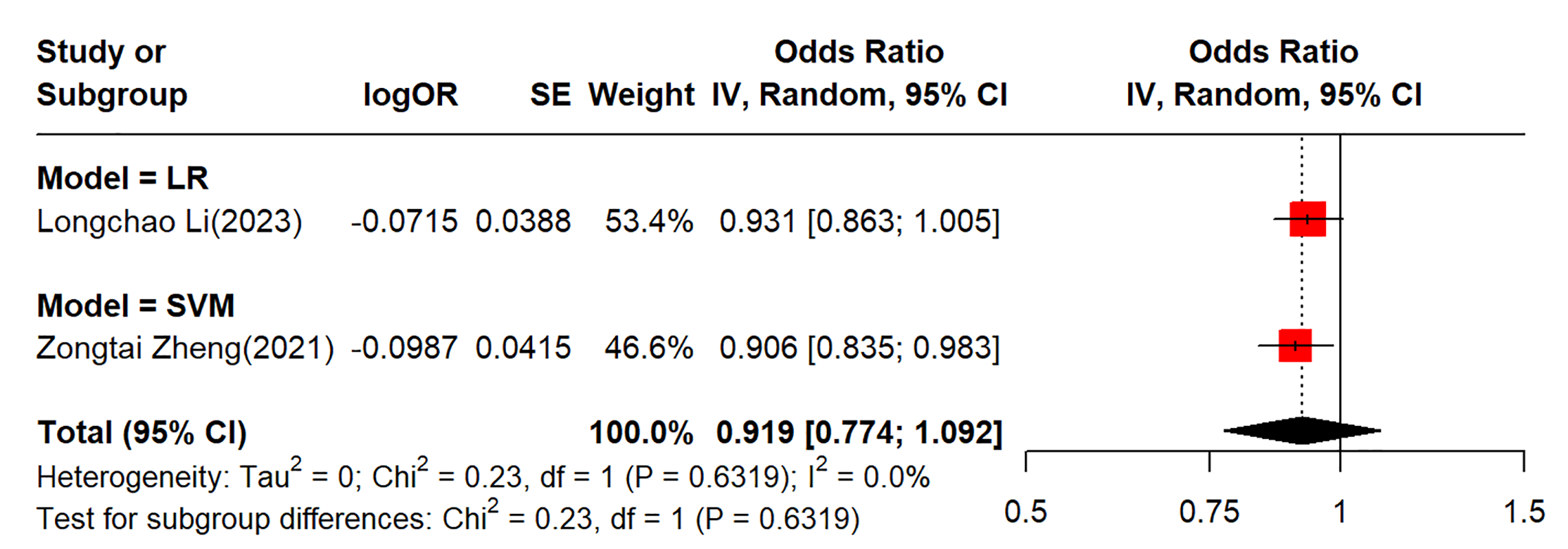
Figure S14** Forest plot of the area under the curve (AUC) for the diagnosis of high-grade tumors using machine learning models based on MRI radiomics combined with clinical features in the validation set [39,53].

**Reference:**

39. Li L, Zhang J, Zhe X, et al. An MRI-based radiomics nomogram in predicting histologic grade of non-muscle-invasive bladder cancer. Front Oncol. 2023;13:1025972. [doi: 10.3389/fonc.2023.1025972] [Medline: 37007156]

53. Zheng Z, Xu F, Gu Z, et al. Integrating multiparametric MRI radiomics features and the Vesical Imaging-Reporting and Data System (VI-RADS) for bladder cancer grading. Abdom Radiol (NY). Sep 2021;46(9):4311-4323. [doi: 10.1007/s00261-021-03108-6] [Medline: 33978825]

**
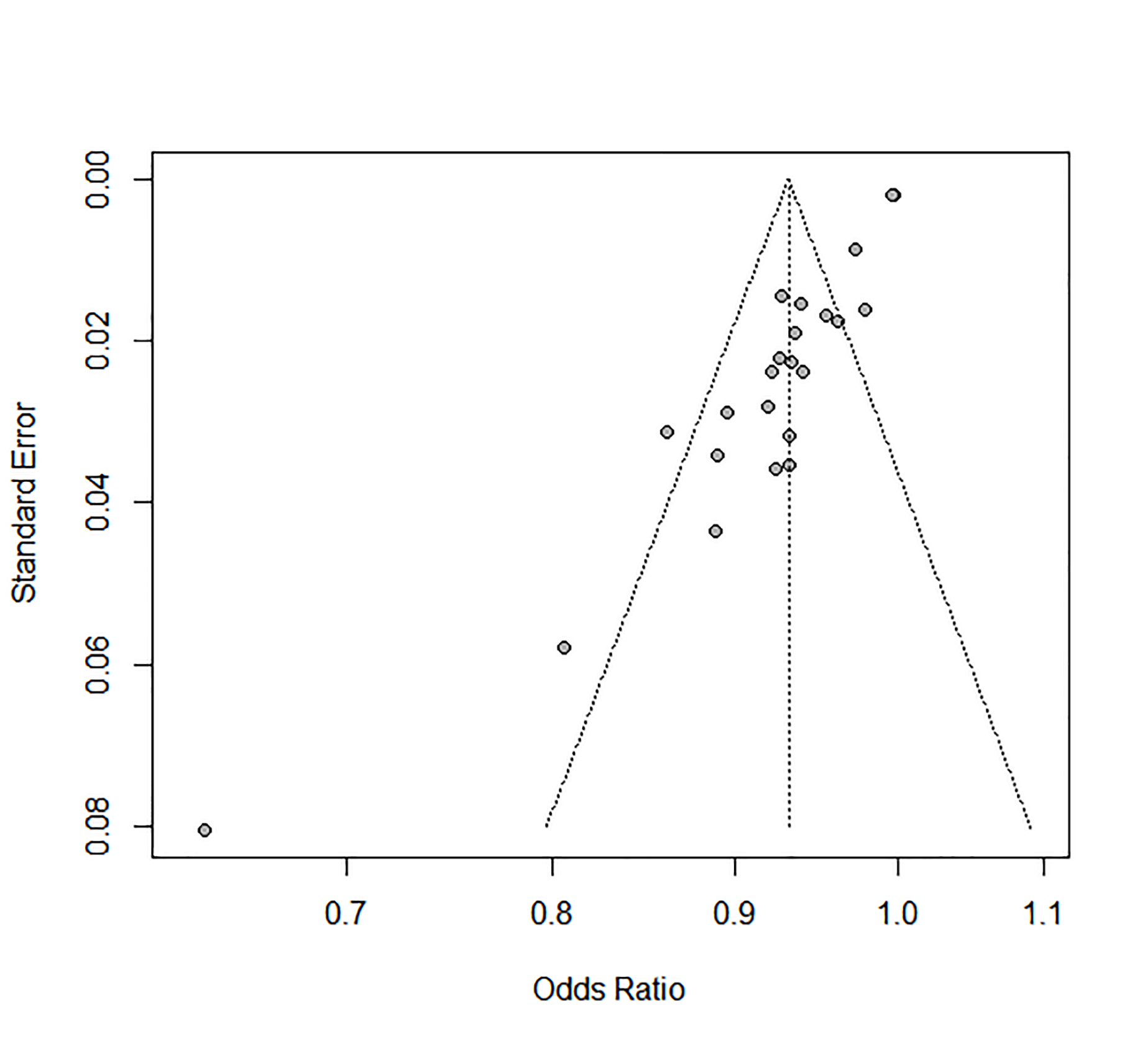
Figure S15** Funnel plot of radiomics-based machine learning for detecting muscle invasion in the training set

**
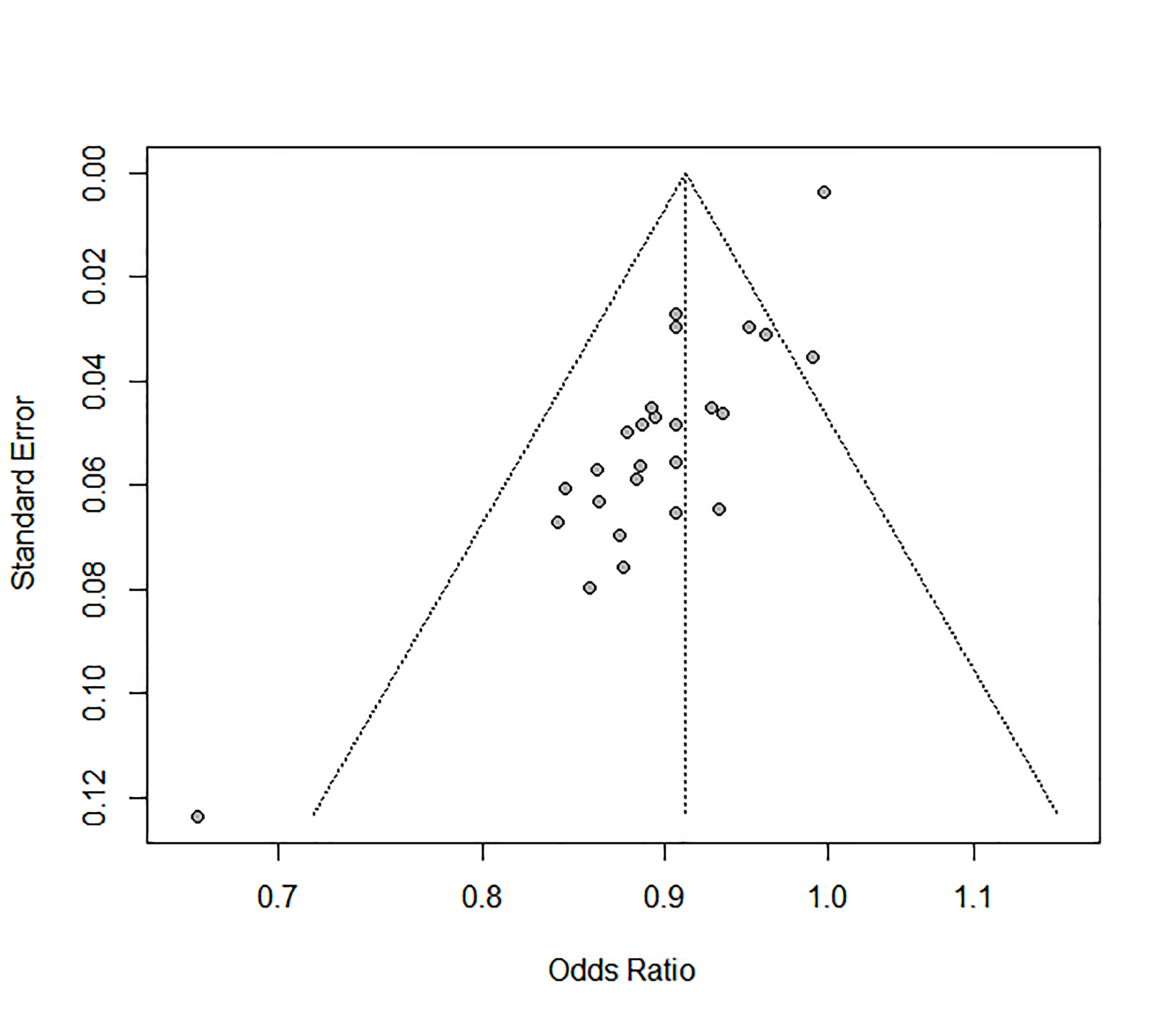
**

Figure S16 Funnel plot of radiomics-based machine learning for detecting muscle invasion in the validation set

**
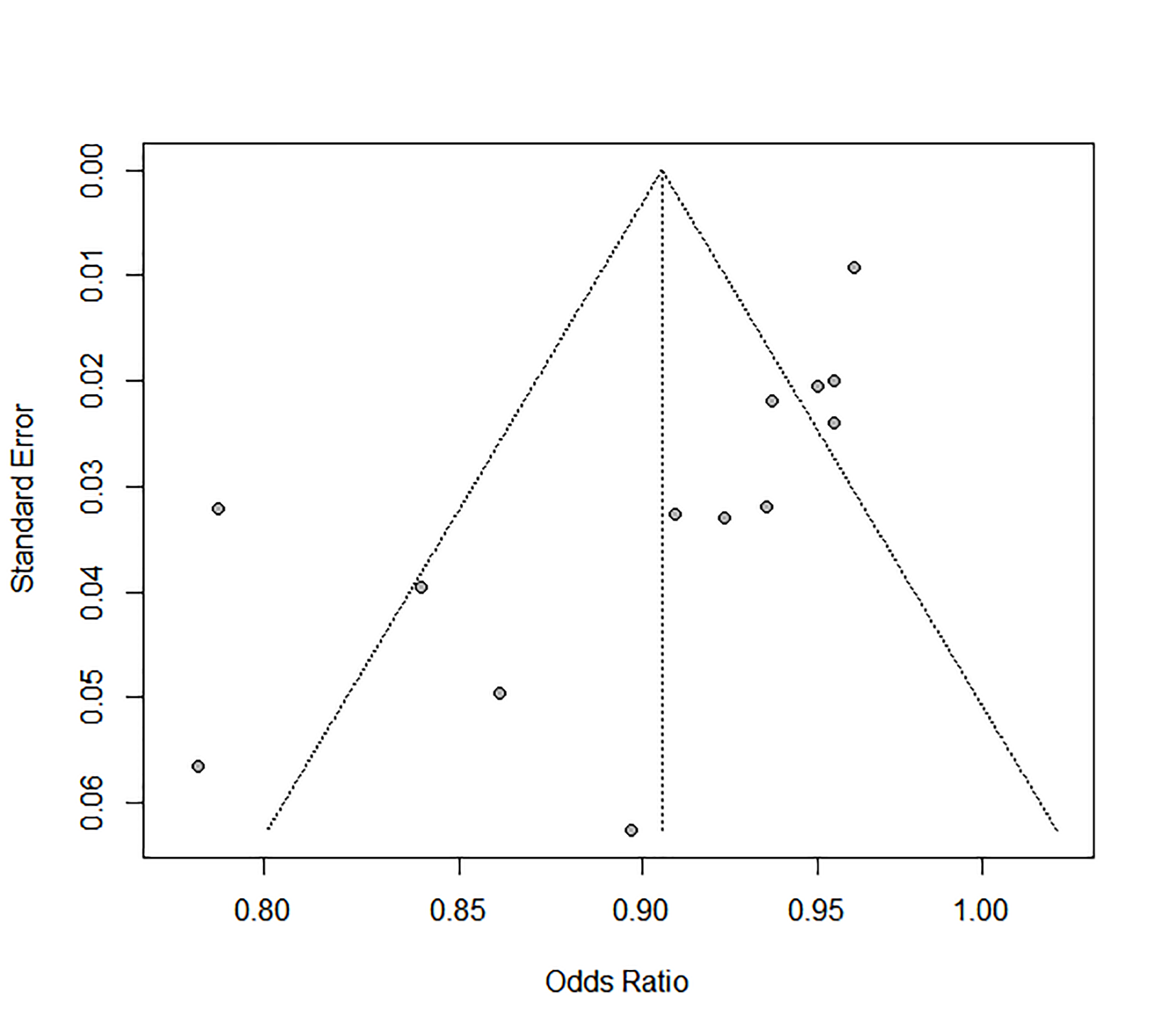
**

**Figure S17** Funnel plot of radiomics-based machine learning for diagnosing high-grade tumors in the training set

**
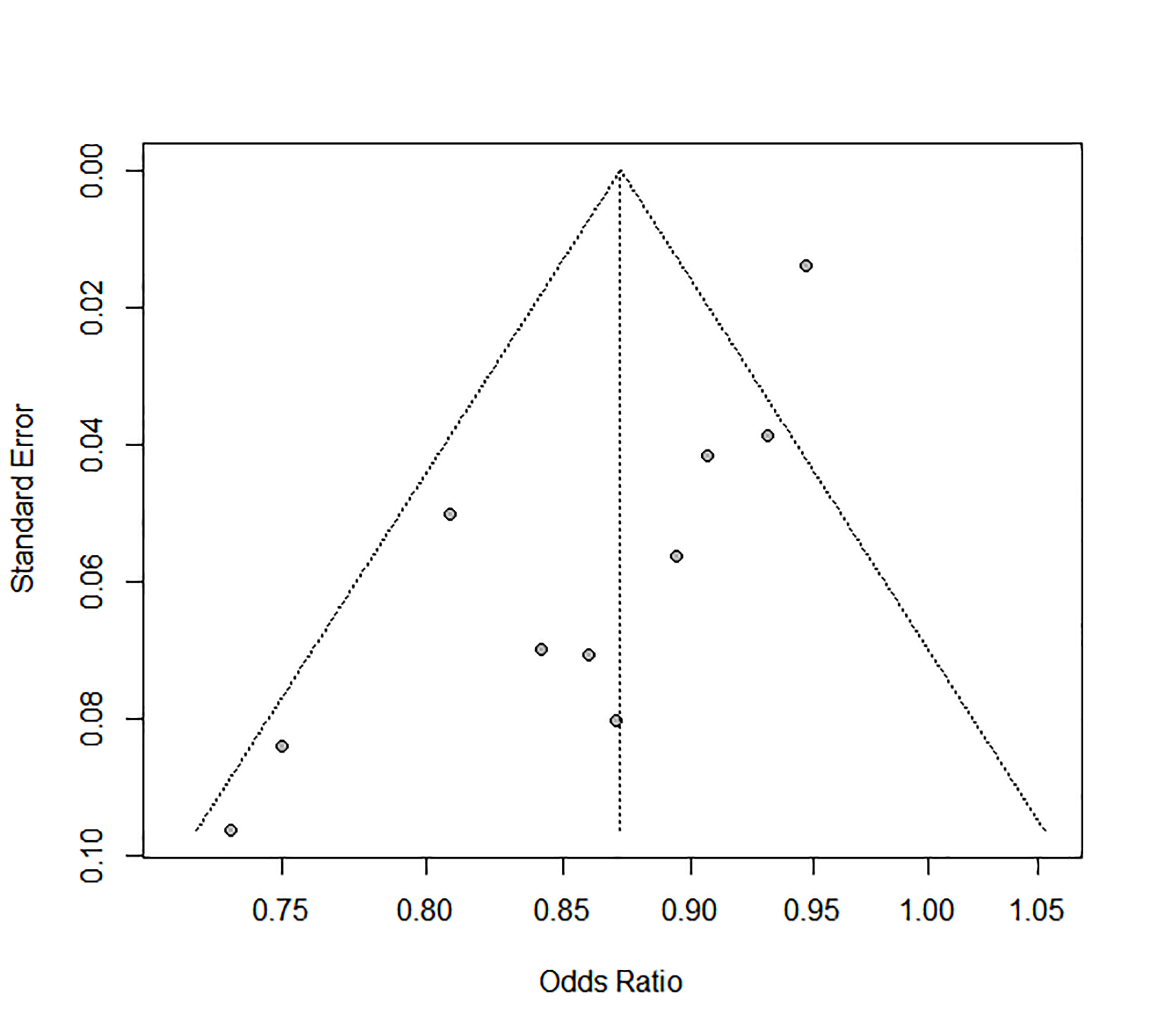
**

**Figure S18** Funnel plot of radiomics-based machine learning for diagnosing high-grade tumors in the validation set
