# Supplementary material for: Application Value of Radiomics-Based Machine Learning for Preoperative Risk Stratification of Bladder Cancer: Systematic Review and Meta-Analysis
Source: J Med Internet Res. 2026 Jun 12;28:e81084. doi: 10.2196/81084 (PMC13263024; doi:10.2196/81084)
Supplement: Multimedia Appendix 4 [file jmir-v28-e81084-s004.docx]

**Table S2** Meta-regression of AUC for machine learning models based on CT and MRI radiomics in detecting muscle invasion (training set)

| Image | Factors | B | se | T | P | 95% CI |
| --- | --- | --- | --- | --- | --- | --- |
| CT |  |  |  |  |  |  |
|  | samplesize | 0.0004 | 0.0006 | 0.696 | 0.506 | -0.0010 - 0.0019 |
|  | Model type |  |  |  |  |  |
|  | LR(Reference) |  |  |  |  |  |
|  | Other ML | -0.0140 | 0.0803 | -0.174 | 0.866 | -0.1990 - 0.1711 |
|  | Variable |  |  |  |  |  |
|  | Radiomics(Reference) |  |  |  |  |  |
|  | Radiomics+Clinical | -0.0076 | 0.0767 | -0.099 | 0.9236 | -0.1845 – 0.1693 |
| MRI |  |  |  |  |  |  |
|  | samplesize | 0.0001 | 0.0001 | 0.5653 | 0.5857 | -0.0002 - 0.0004 |
|  | Model type |  |  |  |  |  |
|  | LR(Reference) |  |  |  |  |  |
|  | Other ML | -0.0206 | 0.0391 | -0.5269 | 0.611 | -0.1090 - 0.0678 |
|  | Variable |  |  |  |  |  |
|  | Radiomics(Reference) |  |  |  |  |  |
|  | Radiomics+Clinical | -0.0082 | 0.0383 | 0.2128 | 0.836 | -0.0786 - 0.0949 |
