# Supplementary material for: Application Value of Radiomics-Based Machine Learning for Preoperative Risk Stratification of Bladder Cancer: Systematic Review and Meta-Analysis
Source: J Med Internet Res. 2026 Jun 12;28:e81084. doi: 10.2196/81084 (PMC13263024; doi:10.2196/81084)
Supplement: Multimedia Appendix 5 [file jmir-v28-e81084-s005.docx]

**Table S3** Meta-regression of AUC for machine learning models based on CT and MRI radiomics in detecting muscle invasion (validation set)

| Image | Factors | B | se | T | P | 95% CI |
| --- | --- | --- | --- | --- | --- | --- |
| CT |  |  |  |  |  |  |
|  | samplesize | -0.0003 | 0.0009 | -0.3517 | 0.732 | -0.0022 - 0.0016 |
|  | Model type |  |  |  |  |  |
|  | LR(Reference) |  |  |  |  |  |
|  | Other ML | -0.0007 | 0.0537 | -0.0137 | 0.989 | -0.1204 - 0.1189 |
|  | Variable |  |  |  |  |  |
|  | Radiomics(Reference) |  |  |  |  |  |
|  | Radiomics+Clinical | -0.0306 | 0.0536 | -0.5714 | 0.580 | -0.1499 – 0.0887 |
| MRI |  |  |  |  |  |  |
|  | samplesize | -0.0004 | 0.0002 | -1.8416 | 0.090 | -0.0009 - 0.0001 |
|  | Model type |  |  |  |  |  |
|  | LR(Reference) |  |  |  |  |  |
|  | Other ML | -0.0233 | 0.0377 | -0.6193 | 0.547 | -0.1055 - 0.0588 |
|  | Variable |  |  |  |  |  |
|  | Radiomics(Reference) |  |  |  |  |  |
|  | Radiomics+Clinical | -0.0221 | 0.0357 | -0.6199 | 0.547 | -0.0999 - 0.0557 |
